# Ultrasmall targeted nanoparticles with engineered antibody fragments for imaging detection of HER2-overexpressing breast cancer

**DOI:** 10.1038/s41467-018-06271-5

**Published:** 2018-10-08

**Authors:** Feng Chen, Kai Ma, Brian Madajewski, Li Zhuang, Li Zhang, Keith Rickert, Marcello Marelli, Barney Yoo, Melik Z. Turker, Michael Overholtzer, Thomas P. Quinn, Mithat Gonen, Pat Zanzonico, Anthony Tuesca, Michael A. Bowen, Larry Norton, J. Anand Subramony, Ulrich Wiesner, Michelle S. Bradbury

**Affiliations:** 1 Department of Radiology, Sloan Kettering Institute for Cancer Research, New York, NY 10065 USA; 2 Department of Materials Science & Engineering, Cornell University, Ithaca, NY 14853 USA; 3 MedImmune, LLC, One MedImmune Way, Gaithersburg, MD 20878 USA; 4 Cell Biology Program, Sloan Kettering Institute for Cancer Research, New York, NY 10065 USA; 5 BCMB Allied Program, Weill Cornell Medical College, New York, NY 10065 USA; 6 Department of Biochemistry, University of Missouri, Columbia, MO 65211 USA; 7 Harry S. Truman Veterans’ Hospital, Columbia, MO 65201 USA; 8 Department of Epidemiology and Biostatistics, Sloan Kettering Institute for Cancer Research, New York, NY 10065 USA; 9 Department of Medical Physics, Sloan Kettering Institute for Cancer Research, New York, NY 10065 USA; 10 Department of Medicine & Office of the President, Memorial Sloan Kettering Cancer Center, New York, NY 10065 USA; 11 Molecular Pharmacology Program, Sloan Kettering Institute for Cancer Research, New York, NY 10065 USA

## Abstract

Controlling the biodistribution of nanoparticles upon intravenous injection is the key to achieving target specificity. One of the impediments in nanoparticle-based tumor targeting is the inability to limit the trafficking of nanoparticles to liver and other organs leading to smaller accumulated amounts in tumor tissues, particularly via passive targeting. Here we overcome both these challenges by designing nanoparticles that combine the specificity of antibodies with favorable particle biodistribution profiles, while not exceeding the threshold for renal filtration as a combined vehicle. To that end, ultrasmall silica nanoparticles are functionalized with anti-human epidermal growth factor receptor 2 (HER2) single-chain variable fragments to exhibit high tumor-targeting efficiency and efficient renal clearance. This ultrasmall targeted nanotheranostics/nanotherapeutic platform has broad utility, both for imaging a variety of tumor tissues by suitably adopting the targeting fragment and as a potentially useful drug delivery vehicle.

## Abstract (web summary)

One of the major obstacles in nanoparticle-based therapy is to achieve tumour targeting, limiting non-specific accumulation of the nanoparticles. Here the authors propose the conjugation of anti-HER2 scFv fragments to the silica nanoparticles, increasing specificity and limiting the final size of the immunoconjugates below the renal clearance threshold.

## Introduction

Breast cancer is the second leading cause of death among women aged 40–59 years in the US, with over 40,000 women expected to succumb to this disease in 2017^1^. Human epidermal growth factor receptor 2 (HER2), a type I transmembrane glycoprotein, plays a critical role in triggering the downstream signaling cascades that control cell proliferation, survival, and apoptosis in breast cancer^2^. It has been known for decades that HER2 is gene-amplified in 20–25% of breast cancer patients, and is associated with a more aggressive phenotype and poorer prognosis^3^, making it an appealing target for both diagnosis and therapy. The advancement of next-generation technologies that can serve as quantitative imaging biomarkers for diagnostic and therapeutic monitoring of HER2-positive (HER2+) disease in breast cancer patients has been widely touted as an effective approach for transforming oncological care^4,5^, although it continues to face critical hurdles. Despite various conventional imaging modalities, such as magnetic resonance imaging, ultrasound, and computerized tomography (CT), showing potential in terms of improving the diagnostic specificity of screening X-ray mammography^6,7^, no single imaging technique, to date, can achieve high enough detection sensitivity and specificity to significantly impact the management of this disease.

HER2-targeted positron emission tomography (PET)/CT imaging has shown significant progress in terms of its ability to sensitively and accurately identify HER2+ primary and metastatic lesions^8^. This technique can obviate the need for repeated biopsies, in addition to enabling non-invasive detection and monitoring of HER2 expression levels in lesions for which biopsies are not possible. Matching the half-lives of positron-emitting radionuclides, such as copper-64 (^64^Cu, *t*_1/2_ = 12.6 h)^9^ and zirconium-89 (^89^Zr, *t*_1/2_ = 78.4 h)^10^, with the biological half-lives (*t*_1/2_ ~100 h) of anti-HER2 monoclonal antibodies (mAbs), such as Trastuzumab, prior to their attachment, has led to the successful imaging of HER2 expression in breast cancer patients^11^. Despite ongoing human trials worldwide, the clinical application of intact antibody-based immuno-PET imaging is hampered by prolonged blood circulation half-lives, inefficient tumor penetration, and pronounced off-target (i.e., liver and, most notably, bone marrow) localization and irradiation, and extended “wait times” (i.e., 4–5 days) before high-contrast imaging of the target site can be achieved. Although smaller-sized anti-HER2 immune fragments, such as F(ab’)_2_^12^, F(ab’)^13^, single-chain variable fragment (scFv)^14^, single domain antibody fragment^15^, and affibody molecules^16^, could enable high-contrast PET imaging at the target site at earlier time (i.e., 2–3 h post-injection (p.i.)), and with better tumor tissue penetration, this is generally precluded due to their typically shorter blood circulation half-lives (minutes to hours) and faster whole-body clearance rates. In fact, these immune fragment-based radioimmunoconjugates often reveal significantly reduced tumor uptake, while high levels of radioactivity accumulate at off-target sites, such as the liver and kidney^17^, leading to excessive normal-organ doses, as well as an inability to visualize metastatic lesions.

Over the past decade, considerable efforts have focused on combining nanodelivery vehicles with immune components, such as mAb or immune fragments, to develop highly versatile HER2-targeting nanocarriers; these vehicles have included nanographene oxide^18^, iron oxide^19^, liposomes^20,21^, quantum dot-conjugated immunoliposomes^22^, carbon nanotubes^23^, and polymeric nanoparticles^24^. Although demonstrating specific in vitro targeting of HER2-expressing cancer cells, these nanocarriers were significantly hampered by their large hydrodynamic diameter (HD) (>20 nm), which exceeded the renal clearance cutoff, leading to rapid hepatic clearance and/or reticuloendothelial system (RES) uptake, severe aggregation, as well as low cumulative tumor uptake. Furthermore, quantitative imaging assessments of pharmacokinetic activity were often precluded due to the absence of a suitable imaging label. Given these technical hurdles, the unmet need of delivering a safe and clinically-promising multifunctional HER2-targeting agent for advancing breast cancer care has become paramount, and necessitates paradigm-shifting designs not yet attained.

Herein, we report on a ^89^Zr-labeled anti-HER2-targeted ultrasmall silica nanoparticle immunoconjugate which is renally-clearable, as well as exhibits distinct biological properties for breast cancer diagnosis and treatment. Fluorescent core–shell silica nanoparticles, termed Cornell dots or C dots^25,26^, with HD below 10 nm constitute a versatile optical materials platform incorporating near-infrared (NIR) dyes in the core and a variety of targeting ligands and contrast-producing radiolabels in the shell, yielding a product that has been successfully translated into the clinic^27,28^. In this study, we focus on next-generation ultrasmall Cornell prime dots (or C’ dots) synthesized in water^29^, with improved size control, high particle brightness, and modular and orthogonal functionalization capabilities of the poly(ethylene glycol) (PEG) shell^30^. Specifically, 6–7 nm-sized Cy5 dye encapsulating and PEGylated C’ dots (i.e., PEG-Cy5-C’ dots) have been adapted with chelators, anti-HER2 scFv fragments, and radiolabels following surface amination. Site-specific engineering of anti-HER2 scFv fragments leads to an efficient click chemistry bioconjugation process. This ultrasmall, particle-based design addresses current challenges in nanomedicine and immune targeting of HER2+ breast cancer, and achieves (1) highly specific HER2-targeted PET imaging with substantially reduced off-target accumulations in the RES or kidney over a 24 h period; (2) bulk renal clearance without excessive kidney irradiation; (3) very high tumor-targeting efficiency and high tumor-to-background ratios not attainable with scFv molecules or scFv-conjugated nanoparticles larger than 10 nm in diameter.

## Results

### Site-specific engineering of anti-HER2 scFv fragments

**Fig. 1:**
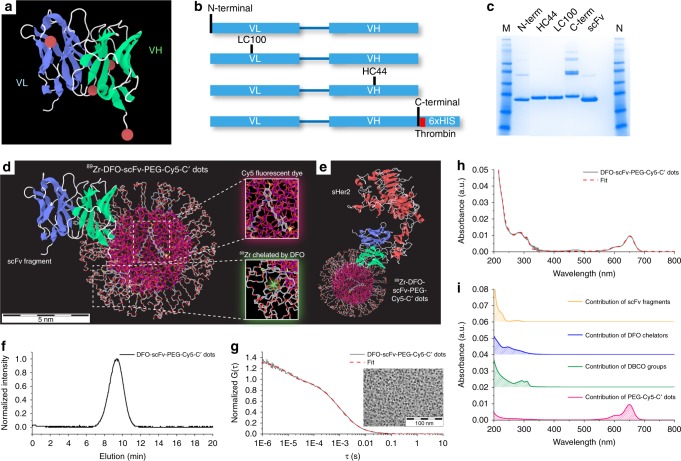
Site-specific engineering of anti-HER scFv fragments and synthesis of DFO-scFv-PEG-Cy5-C’ dots. **a** Three-dimensional (3D) scFv model highlighting sites of conjugation (i.e., red balls). **b** Schematic representation of scFvs directed to HER2/neu extracellular domain with sites of nnAA integration indicated (N-terminal, LC100, HC44, and C-terminal). Sites were rationally designed to be distal to the antigen binding domains (blue ribbon) and surface-exposed to avoid functionally important domains. **c** Affinity purified scFvs containing a nnAA at the indicated positions by SDS-PAGE under non-reducing conditions; HC44 and LC100 constructs generated mostly unique bands. scFv constructs with N-terminal, and in particular with C-terminal, nnAA produced disulfide-linked multimers observable as discrete bands of increasing molar mass. Unmodified scFv was resolved for comparison. **d, e** 3D rendering of ^89^Zr-DFO-scFv-PEG-Cy5-C’ dot (**d**) and ^89^Zr-DFO-scFv-PEG-Cy5-C’ dot bonded to sHER2 (**e**). The atoms of silicon, oxygen, carbon, nitrogen, sulfur, and zirconium in the 3D renderings are colored in purple, red, gray, blue, yellow, and light green, respectively. Hydrogen atoms are not displayed for better visualization. **f**–**i** GPC elugram (**f**), FCS correlation curve with fit (**g**), and UV–vis absorbance spectrum with fit (**h**) of DFO-scFv-PEG-Cy5-C’ dots. **i** Deconvolution of the UV–vis spectrum (**h**) into contributions of individual functional moieties, including PEG-Cy5-C’ dot (red), DBCO (green), DFO (blue), and scFv (orange). A representative TEM image of DFO-scFv-PEG-Cy5-C’ dots is shown in the inset of **g**

An scFv format of the antibody was generated based on Trastuzumab, using a variable light/heavy chain (VL-linker-VH) construct. The roughly 25-kDa scFv format was chosen as a small antibody-based targeting ligand to maintain the size of the resulting particle immunoconjugate below the renal clearance cut-off. However, the small size of the scFv fragment provides only a few viable conjugation sites that do not interfere with antigen-binding function. Thus, site-specific conjugation methods were essential to control the position and number of conjugation sites, while ensuring that each targeting agent contained a single conjugation site to avoid nanoparticle crosslinking. Four candidate sites (Fig. 1a, red balls) were initially identified based upon available crystal structures of model scFv fragments^31^. Each site was predicted to have conjugation positions distal to the antigen-binding domain, and at surface-exposed residues that should enable efficient conjugate formation. These consisted of two heavy chain (HC) positions—C-terminal and an intragenic site, referred to as position HC44—and two light chain (LC) sites, termed N-terminal and LC100 (Fig. 1b). Sites of conjugation were controlled by genetically-encoding a non-natural amino acid (nnAA) using cells expressing an orthogonal Pyl tRNA synthetase and tRNApyl from *Methanosarcinae mazei*^32^. The synthetase has specificity for N6-((2-azidoethoxy)carbonyl)-L-lysine (AzK), a lysine analogue containing an azide group that enables click cycloaddition chemistry, and its cognate amber suppressor tRNApyl, that functions to deliver the nnAA in response to amber stop codons^33^. Four DNA expression constructs encoding anti-HER2 scFvs were assembled, each containing an amber codon at the selected position. Each positional variant was expressed, purified (Fig. 1c), and functionally characterized to identify PEG-modified scFvs that were stable, efficiently conjugated, and the conjugates retained antigen binding (see Supplementary Discussion Sections 2.1-2.2, Supplementary Figs. 1–4 and Supplementary Table 1). On the basis of the foregoing results, the anti-HER2 scFv containing the nnAA at HC44 was selected for subsequent C’ dot bioconjugation studies.

### Synthesis and characterization of ultrasmall DFO-scFv-PEG-Cy5-C’ dots

^89^Zr-DFO-scFv-PEG-Cy5-C’ dots (Fig. 1d, e, DFO: deferoxamine), were synthesized as part of a multi-step procedure (Supplementary Fig. 5) that integrated five functional moieties within a single 6–7 nm silica nanoparticle: encapsulated Cy5 fluorescent dye, PEG stealth layer, DBCO (i.e., dibenzocyclooctyne), ^89^Zr-chelated DFO and scFv fragments. Aminated PEG-NH_2_-Cy5-C’ dots, having a HD of 6.6 nm, were first prepared based on previously reported protocols (Supplementary Fig. 6a, b)^29,30^. The resulting PEG-NH_2_-Cy5-C’ dots were subjected to gel permeation chromatography (GPC), fluorescence correlation spectroscopy (FCS), and UV–vis spectroscopic characterization (Supplementary Fig. 7)^25,29,30^. FCS measurements and quantitative analysis of the FCS correlation curves suggested that PEG-NH_2_-Cy5-C’ dots had an average HD of 6.6 nm and contained about 2.2 dyes in each particle (Supplementary Fig. 7c; Supplementary Table 2). Next, aminated PEG-NH_2_-Cy5-C’ dots were reacted with dibenzocyclooctyne-PEG_4_-N-hydroxysuccinimidyl ester (DBCO-PEG_4_-NHS ester) at pH 7.4, resulting in DBCO-PEG-NH_2_-Cy5-C’ dots with controllable DBCO surface density. Afterwards, p-SCN-Bn-deferoxamine (DFO-NCS) was added and reacted with remaining amine groups to form DFO-DBCO-PEG-Cy5-C’ dots. Separation of unreacted DFO and DBCO moieties was achieved by passing the reaction mixture through a PD-10 column. Subsequently, DFO-DBCO-PEG-Cy5-C’ dots were mixed with pre-synthesized anti-HER2 scFv-azide (position HC44, Fig. 1b) in PBS and reacted overnight, yielding DFO-scFv-PEG-Cy5-C’ dots. Significant efforts were devoted to optimizing the surface density of both DBCO and scFv functional moieties to ensure bulk renal clearance and low RES uptake. (Supplementary Figs. 8–14, Supplementary Discussion, Section 2.3).

As-synthesized DFO-scFv-PEG-Cy5-C’ dots were then evaluated by a combination of techniques, including GPC, FCS, transmission electron microscopy (TEM) and UV–vis spectrophotometry. No impurities were identified by GPC, demonstrating the successful removal of all unreacted reagents from the reaction solution (Fig. 1f). The average HD of the GPC-purified DFO-scFv-PEG-Cy5-C’ dots was determined to be 7.3 nm by fitting the FCS correlation curve (Fig. 1g), consistent with TEM observations (inset in Fig. 1g). As compared to the average size of PEG-NH_2_-Cy5-C’ dots (i.e., 6.6 nm), such an increase in particle size is due to the attachment of additional functional ligands (i.e., DBCO, DFO, and scFv) to the C’ dot surface (Supplementary Discussion, Section 2.4 and Supplementary Fig. 15). The UV–vis absorbance spectrum of DFO-scFv-PEG-Cy5-C’ dots exhibited a strong absorption signal at around 650 nm (Fig. 1h), consistent with the absorption of Cy5 fluorescent dye. To quantify the number of functional ligands per C’ dot, the UV–vis absorbance was fit by a linear combination of the absorption spectra of each of the individual components, including Cy5, DBCO, DFO and scFv (Fig. 1h)^30^. As a result, the UV–vis absorption spectrum of DFO-scFv-PEG-Cy5-C’ dots was successfully deconvoluted (Fig. 1i), and the numbers of Cy5, DBCO, DFO and scFv per C’ dot were estimated to be around 1.9, 22, 6.6, and 1.4, respectively (Supplementary Table 2, Supplementary Discussion, Sections 2.5 and 2.6, Supplementary Figs. 16, 17). Based on these results, DFO-scFv-PEG-Cy5-C’ dots bearing ~1.4 scFv per C’ dot were selected for in-depth immunoconjugate characterization, in vitro HER2 active targeting studies, radiolabeling, and in vivo HER2-targeted PET imaging studies.

### In vitro targeting in HER2-expressing breast cancer cells

**Fig. 2:**
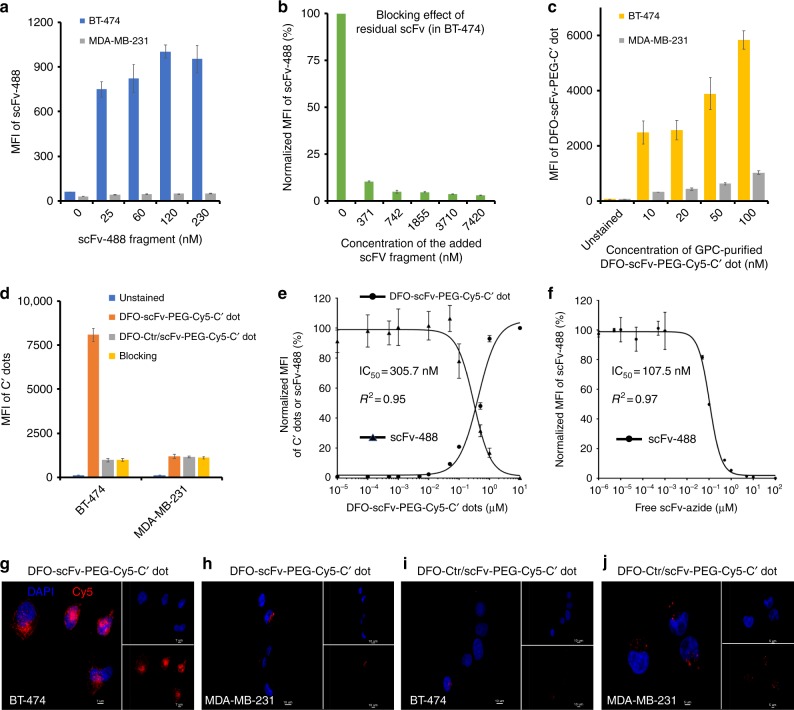
In vitro HER2-specific targeting and half maximal concentrations (IC_50_) of free scFv and DFO-scFv-PEG-Cy5-C’ dots using flow cytometry. **a** In vitro concentration-dependent HER2 targeting of scFv-488 in BT-474 and MDA-MB-231 cell lines. **b** Blocking of scFv-488 binding to HER2 receptors expressed on BT-474 cells as a function of scFv-azide concentration. **c** HER2-targeted uptake of GPC-purified DFO-scFv-PEG-Cy5-C’ dots in BT-474 and MDA-MB-231 cells as a function of particle concentration. All *p* values < 0.001. **d** HER2 targeting specificity of DFO-scFv-PEG-Cy5-C’ dots (100 nM). DFO-Ctr/scFv-PEG-Cy5-C’ dots (100 nM) were used as a particle control. For the blocking group, a 20-fold excess of free scFv-azide fragment was used. All *p* values < 0.001. **e** Concentration-dependent inhibition of scFv-488 binding to BT-474 cells by DFO-scFv-PEG-Cy5-C’ dots. IC_50_ = 305.7 nM. **f** Concentration-dependent inhibition of scFv-488 binding to BT-474 cells by free scFv-azide fragments. IC_50_ = 107.5 nM. Each data point represents the mean ± s.d. of three replicates. Confocal microscopy imaging of DFO-scFv-PEG-Cy5-C’ dots (100 nM) in **g** BT-474 (scale bar: 7 μm) and **h** MDA-MB-231 (scale bar: 10 μm), and DFO-Ctr/scFv-PEG-Cy5-C’ dots (100 nM) in **i** BT-474 (scale bar: 10 μm) and **j** MDA-MB-231 (scale bar: 5 μm). Blue and red colors represent DAPI and Cy5, respectively. Insets in **g**–**j** show the corresponding DAPI and Cy5 channels

Prior to conjugating scFv fragments to DFO-DBCO-PEG-Cy5-C’ dots, HER2-specific targeting capabilities were demonstrated by incubating the dye-conjugated scFv fragments (i.e., scFv-488, Supplementary Fig. 16) with two well-established breast cancer cell lines exhibiting different HER2 expression signatures, as previously reported^34^ and confirmed by our western blots (Supplementary Fig. 18): BT-474 (HER2+, 3.7 × 10^6^ receptors per cell) and MDA-MB-231 (HER2−, 7.0 × 10^4^ receptors per cell). The results presented in Fig. 2a demonstrate a significant dose-dependent increase in scFv-488 median fluorescence intensity (MFI) upon binding BT-474 cells, in contrast to that seen with MDA-MB-231 cells. Maximum MFI enhancement (i.e., 20-fold) was achieved with increasing scFv-488 concentrations up to 120 nM, beyond which no significant changes in the signal intensity were noted, suggesting saturation of available HER2 receptors on cell surfaces. The anti-HER2-specific binding and internalization of scFv-488 were further confirmed using confocal microscopy imaging (Supplementary Fig. 19).

Results of this dose–response study were used to interpret the findings of HER2 competitive binding studies with C’ dot immunoconjugates. In particular, selective HER2 receptor targeting using C’ dots functionalized with scFv fragments (i.e., about 1.4 scFvs per particle) was found to be limited by competitive binding of un-purified free scFv fragments (Supplementary Fig. 20). Estimates indicated that the concentration of free scFv-azide was about 3 times higher than that of DFO-scFv-PEG-Cy5-C’ dots in solution without proper purification of free scFv fragments. The degree of blocking by residual scFv fragments was quantitatively assessed in vitro by co-incubating scFv-488 with scFvs over a range of concentrations. As shown in Fig. 2b, an estimated 90% of the blocking effect was observed when ~400 nM of free scFv was used. This result explains the fluorescence-activated cell sorting (FACS) data (Supplementary Fig. 20), where no appreciable enhanced uptake of DFO-scFv-PEG-Cy5-C’ dots in HER2+ cells was seen relative to that found for HER2− cells. However, by utilizing a GPC particle purification scheme (Supplementary Discussion, Section 2.5), very high HER2-targeting specificity was achieved. Importantly, BT-474 cells showed a significant concentration-dependent increase in MFI—a factor 6–7 times higher than that found for MDA-MB-231 cells (Fig. 2c)—by incubating cells with GPC-purified DFO-scFv-PEG-C’ dots (Fig. 1f). Although non-specific binding of DFO-scFv-PEG-C’ dots to MDA-MB-231 cells was also found to be concentration-dependent, the magnitude of this effect was substantially less under the same incubation conditions.

HER2-specific targeting was also found by incubating cells with isotype control scFv fragments (i.e., Ctr/scFv) bound to C’ dots (i.e., DFO-Ctr/scFv-PEG-Cy5-C’ dots, Supplementary Fig. 21; Supplementary Table 2), as well as receptor blocking studies (Fig. 2d). In the latter case, DFO-scFv-PEG-Cy5-C’ dots were co-incubated with excess free scFv-azide fragments. As shown in Fig. 2d and Supplementary Fig. 22, an 8-fold reduction in targeted uptake was observed for DFO-Ctr/scFv-PEG-Cy5-C’ dots (100 nM), as against DFO-scFv-PEG-Cy5-C’ dots (100 nM). DFO-scFv-PEG-Cy5-C’ dots showed high target specificity after receptor blocking with excess (20-fold) free scFv-azide (Fig. 2d). No significant difference in MDA-MB-231 cell binding was found among the three groups (Fig. 2d).

In addition, relative HER2 receptor binding affinities of DFO-scFv-PEG-Cy5-C’ dots and scFv-azide were determined using BT-474 cells. Utilizing scFv-488 fragments (40 nM) and FACS analysis, IC_50_ values were determined. An increased IC_50_ value of 305.7 nM (Fig. 2e) was found for DFO-scFv-PEG-Cy5-C’ dots, as against that found for the free scFv-azide fragment (IC_50_ = 107.5 nM, Fig. 2f), indicating that conjugation of scFv-azide fragments to C’ dots does not significantly alter binding affinity. One principal consideration explaining the small increase in the IC_50_ value stems from the possibility that not all C’ dots contain 1.4 scFv fragments. Specific HER2-targeted uptake was also shown by confocal microscopy of DFO-(Ctr/)scFv-PEG-Cy5-C’ dots (100 nM) in BT-474 and MDA-MB-231 cells (Fig. 2g–j). No significant alteration in cell survival or proliferation was found to occur for BT-474 and MDA-MB-231 cells exposed to DFO-scFv-PEG-Cy5-C’ dots (Supplementary Fig. 23). These results underscore the importance of establishing a controlled multi-step process for producing stable sub-10-nm high-affinity particle immunoconjugates which, in turn, can potentially maximize anti-HER2-targeted uptake in HER2+ breast cancer models and achieve favorable whole-body distributions.

### In vivo HER2-targeted PET imaging of ^89^Zr-DFO-scFv-PEG-Cy5-C’ dots

**Fig. 3:**
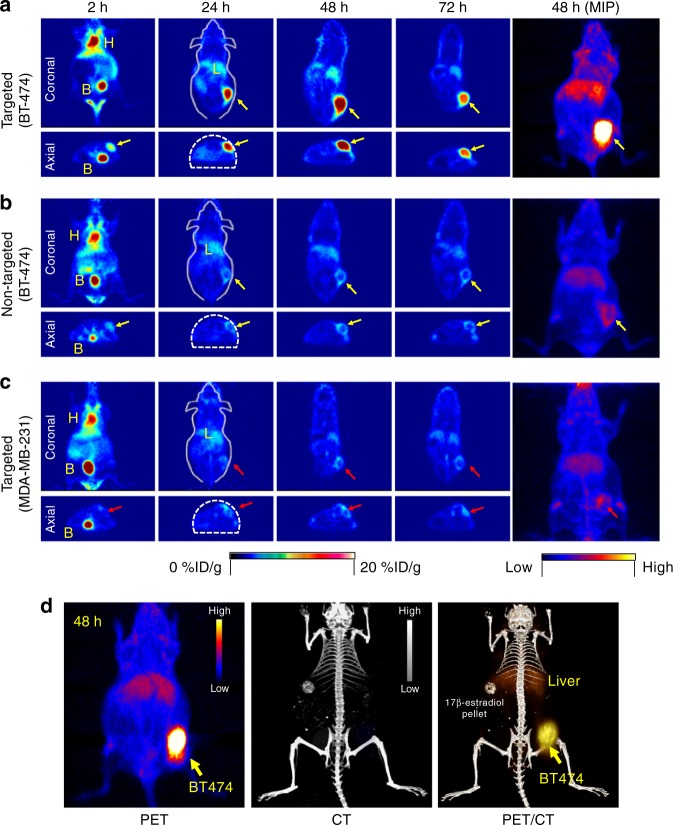
In vivo HER2-targeted PET imaging in xenograft breast cancer models. Serial coronal and axial tomographic PET images acquired at 2, 24, 48, and 72 h post i.v. injection of radiolabeled particle immunoconjugates in groups of tumor-bearing mice (*N* = 5 for each group) as follows—**a** targeted group: ^89^Zr-DFO-scFv-PEG-Cy5-C’ dots in BT-474 mice, **b** non-targeted group: ^89^Zr-DFO-Ctr/scFv-PEG-Cy5-C’ dots in BT-474 mice, and **c** targeted group: ^89^Zr-DFO-scFv-PEG-Cy5-C’ dots in MDA-MB-231 mice. For each group, maximum intensity projection (MIP) images were also acquired at 48 h p.i. H: heart, B: bladder, L: liver. **d** Representative MIP PET, CT, and PET/CT fusion images of ^89^Zr-DFO-scFv-PEG-Cy5-C’ dots in a BT-474 tumor-bearing mouse. All BT-474 tumors were marked with yellow arrows, while all MDA-MB-231 tumors were marked with red arrows

**Fig. 4:**
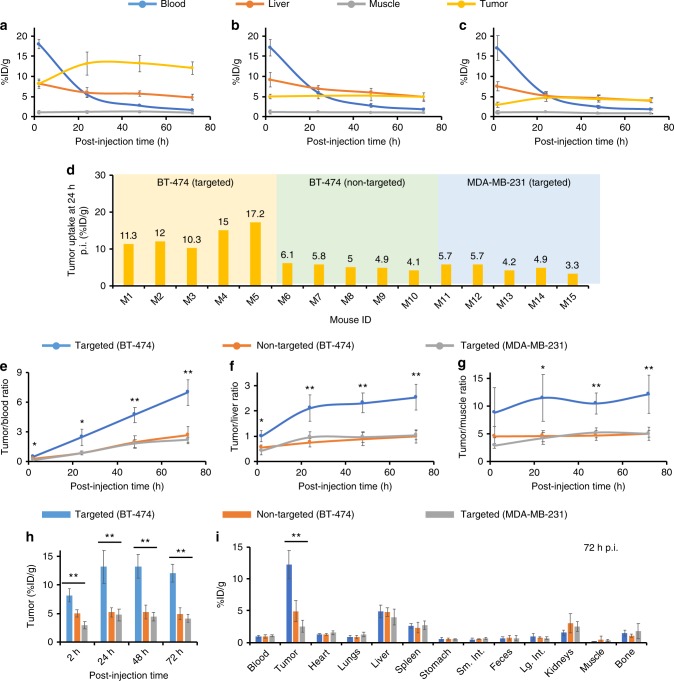
Region-of-interest quantification and biodistribution studies. Time–activity curves showing major organ uptake of ^89^Zr-labeled nanoparticles in mice injected with **a** targeted group: ^89^Zr-DFO-scFv-PEG-Cy5-C’ dots, BT-474 tumor, **b** non-targeted group: ^89^Zr-DFO-Ctr/scFv-PEG-Cy5-C’ dots, BT-474 tumor, and **c** targeted group: ^89^Zr-DFO-scFv-PEG-Cy5-C’ dots, MDA-MB-231 tumor. **d** Variation of tumor uptake (%ID g^−1^) for 3 cohorts of mice. M1–M5: BT-474 tumor-bearing mice injected with ^89^Zr-DFO-scFv-PEG-Cy5-C’ dots (targeted); M6–M10: BT-474 tumor-bearing mice injected with ^89^Zr-DFO-Ctr/scFv-PEG-Cy5-C’ dots (non-targeted); M10–M15: MDA-MB-231 tumor-bearing mice injected with ^89^Zr-DFO-scFv-PEG-Cy5-C’ dots (targeted). Comparisons of **e** tumor-to-blood ratios, **f** tumor-to-liver ratios, **g** tumor-to-muscle ratios, and **h** tumor uptake among three groups. *N* = 5 animals per group. **i** Biodistribution profiles for all animal group at 72 h p.i. *p < 0.01, **p < 0.001. The two degrees-of-freedom *F*-test was followed by pairwise *t*-tests that were adjusted for multiple comparisons using the Holm method. Each data point represents the mean ± s.d. of five replicates

To investigate tumor-targeting efficiency and uptake within HER2+ and HER2− xenograft models (Supplementary Fig. 24), 7.3 nm DFO-scFv-PEG-Cy5-C’ dots were radiolabeled with ^89^Zr and systemically administered to non-tumor-bearing or BT-474 tumor-bearing mice for assessment of biodistribution, radiostability, whole-body clearance (Supplementary Figs. 25-27; Supplementary Table 3; Supplementary Discussion, Section 2.7) and in vivo HER2-targeted uptake. Tomographic PET images (Fig. 3a) showed dominant cardiac activity (18.0 ± 1.1 %ID g^−1^) 2 h p.i. of ^89^Zr-DFO-scFv-PEG-Cy5-C’ dots, indicating that particles were largely confined to the blood pool. Cardiac activity-concentrations gradually decreased over time to 5.5 ± 0.7 %ID g^−1^and 1.7 ± 0.1 %ID g^−1^ at 24 and 72 h p.i., respectively (Fig. 4a). Hepatic uptake was also found to decrease from 8.3 ± 0.8 %ID g^−1^ (2 h p.i.) to 4.9 ± 0.7 %ID g^−1^ (72 h p.i.). These measured murine hepatic uptake values are markedly lower than those previously reported for particle sizes larger than 10 nm^35^. Uptake in muscle remained relatively constant (i.e., ~1 %ID g^−1^) over the imaging period. Similar trends were found for blood, liver, and muscle time–activity profiles derived for two control groups (*n* = 5 per group; Fig. 4b, c): non-targeted (i.e., ^89^Zr-DFO-Ctr/scFv-PEG-Cy5-C’ dots; BT-474 mice) and targeted (i.e., ^89^Zr-DFO-scFv-PEG-Cy5-C’ dots, MDA-MB-231 mice). Dominant bladder uptake was clearly observed in all three groups on both coronal and axial tomographic PET images at 2 h p.i. (Fig. 3a–c). Bladder activity-concentrations ranged from less than 8 to about 30 %ID g^−1^ at 2 h p.i. (Supplementary Fig. 28a), confirming bulk renal excretion for both targeted and non-targeted particles.

Early accumulations of ^89^Zr-DFO-scFv-PEG-Cy5-C’ dots in HER2+ BT-474 tumors could be seen at 2 h p.i. on axial PET images (Fig. 3a, axial view); uptake was estimated to be 8.2 ± 1.1 %ID g^−1^, a value as high as that found for liver (8.3 ± 0.8 %ID g^−1^). Unlike the time–activity data observed for liver (Fig. 4a), however, BT-474 tumor uptake progressively rose over the next 24–48 h, noting an average maximal value of 13.2 ± 2.9 %ID g^−1^ (*n* = 5). The 48-h maximum intensity projection (MIP) PET image (Fig. 3a) clearly showed pronounced BT-474 tumor uptake of ^89^Zr-DFO-scFv-PEG-Cy5-C’ dots. Tumor uptake remained relatively stable over a 72-h period, with values as high as 12.0 ± 1.5 %ID g^−1^ (*n* = 5, Fig. 4a). In the two control groups (Figs. 3b, c, 4b, c), similar particle activity trends were found for major organs/tissues, noting significantly lower tumor uptake values (~5% ID g^−1^) after administering ^89^Zr-DFO-Ctr/scFv-PEG-Cy5-C’ dots in BT-474 tumors (*n* = 5) and ^89^Zr-DFO-scFv-PEG-Cy5-C’ dots in MDA-MB-231 tumors (*n* = 5) (Fig. 4h).

Maximizing target tissue uptake and retention and target-to-background ratios while minimizing non-specific RES uptake, are the principal considerations driving the rational design of targeted nanoprobes used for clinical cancer care^36^. Targeted uptake of ^89^Zr-DFO-scFv-PEG-Cy5-C’ dots in BT-474 xenografted mice ranged from 10.3 %ID g^−1^ to 17.2 %ID g^−1^ (Fig. 4d), likely due to variations in HER2 expression levels; the upper end of this range is among one of the highest reported values for molecularly targeted radiolabeled nanoparticles^36^ (Supplementary Table 4). Statistically significant (*p* < 0.001) specific enhancements were achieved for the BT-474 group (i.e., greater than 3-fold tumor-to-blood (T/B) ratios), as against those for the control group (Fig. 4e). Further, maximum tumor-to-liver (T/L) and tumor-to-muscle (T/M) ratios were 2.5 ± 0.5 and 12.0 ± 3.4, respectively, more than 2-fold higher than those observed for control groups (Fig. 4e–g), thereby representing the probe with the highest T/L ratio in the comparison group (Supplementary Table 4). Representative PET/CT images of BT-474 tumor-bearing mice injected with ^89^Zr-DFO-scFv-PEG-Cy5-C’ dots showed dominant tumor uptake, noting only minor non-specific liver accumulations over 48 h p.i. (Fig. 3d; Supplementary Movie 1). Results were further confirmed by ex vivo histology, which showed low non-specific accumulation in major off-target organs (Fig. 4i). These findings dramatically differ from those observed with silica radioimmunoconjugates having a HD larger than 10 nm^37,38^; in the latter case, liver generally demonstrates the greatest uptake.

**Fig. 5:**
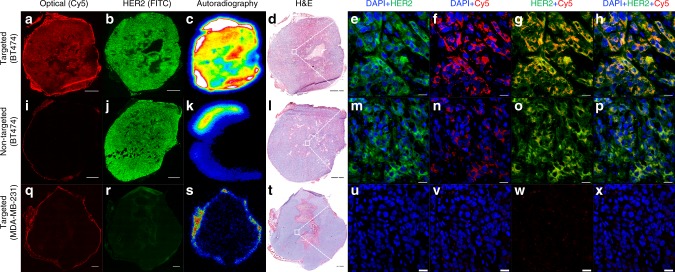
Correlative ex vivo tumor histopathology. From left to right: Cy5-fluorescence microscopy (scale bar: 1 mm), HER2 immunohistochemical staining (scale bar: 1 mm), autoradiography, H&E stained tumor tissue specimens (scale bar: 1 mm) harvested 72 h p.i., and confocal microscopy (scale bar: 20 μm) of random areas (white squares) from corresponding tumor tissue specimens. **a**–**h** Targeted group: ^89^Zr-DFO-scFv-PEG-Cy5-C’ dots, BT-474 tumor, **i**–**p** non-targeted group: ^89^Zr-DFO-Ctr/scFv-PEG-Cy5-C’ dots, BT-474 tumor, and **q**–**x** targeted group: ^89^Zr-DFO-scFv-PEG-Cy5-C’ dots, MDA-MB-231 tumor

We evaluated the penetration and distribution of ^89^Zr-DFO-scFv-PEG-Cy5-C’ dots within ex vivo tumor tissue specimens harvested from all three groups at 72 h p.i. (Fig. 5) by widefield fluorescence microscopy, immunohistochemical staining for HER2 expression, H&E staining, autoradiography, and confocal microscopy of tumor tissue specimens. Both Cy5 widefield microscopy and autoradiographic images confirmed significant tissue penetration and diffusion of targeted particles throughout BT-474 specimens (Fig. 5a–d), suggesting that particles were likely uniformly distributed between the intracellular and interstitial compartments. By contrast, significantly lower optical signal and tracer activity were seen for particle (Fig. 5i–l) and biological (Fig. 5q–t) control specimens. In the latter cases, particles predominantly localized along the tumor periphery and were seen within stromal tissue. High-resolution imaging comparisons among all tumor tissue specimens (Fig. 5e–h, m–p, u–x) further confirmed co-localization of particles and HER2 expression.

Additional in vivo studies were performed in orthotopic BT-474 models, as well as in xenograft models over longer time intervals (i.e., 10 days), to evaluate HER2-specific targeted uptake and PK profile differences (Supplementary Discussion, Sections 2.8-2.9). Notably, similar specific enhancements, such as the T/L ratio, were found between the models at 72 h p.i. Finally, acute toxicity testing was also conducted in an additional cohort of mice by i.v.-injecting “cold” DFO-scFv-PEG-Cy5-C’ dots at doses ~5-fold greater than those used for PET imaging studies. No acute toxicity or tissue-specific pathologic effects were demonstrated (Supplementary Fig. 34, Supplementary Tables 5–9).

## Discussion

The development of cancer-targeted imaging tools as biomarkers for specifically and quantitatively assaying the HER2 status of both primary and metastatic lesions can permit molecular stratification of breast cancers for efficient clinical decision-making and treatment management. Such an imaging-driven stratification would complement genomic drivers to facilitate identification of patients who might benefit from current anti-HER2 therapies^39^, as well as provide non-invasive assessments of treatment response. Immunohistochemical analyses and fluorescence in-situ hybridization procedures, two FDA-approved methodologies for assaying HER2 status of breast cancer biopsy tissue specimens, provide data on HER2 protein expression levels and gene amplification^40^. However, due to sampling bias and tumor heterogeneity, both within and across patient-derived tumor tissue specimens, HER2 expression levels from a limited number of tissue biopsy cores cannot always be accurately characterized and require an invasive procedure that can significantly impair quality of life for these patients^41^. Moreover, prior studies have shown a high discordance in HER2 expression in primary vs metastatic lesions^42^. To address these clinical challenges, PET radionuclides, such as ^68^Ga, ^64^Cu, and ^89^Zr, have been used to label immunoconjugates as investigational new drugs for first-in-human clinical trials (Supplementary Table 10). Collectively, results have been promising in terms of their ability to image HER2 status and/or monitor treatment response in breast cancer patients. However, no single agent has successfully been advanced beyond early-phase clinical trials, which, in part, may stem from unfavorable pharmacokinetic profiles (i.e., extended circulation half-live) and/or physicochemical properties that can lead to dose-limiting toxicities, particularly to the liver, bone marrow, and kidneys. Overcoming these translational obstacles have fueled the further development of advanced, clinically-promising probes that can specifically and quantitatively assay the molecular signatures of breast cancer lesions, markedly reduce off-target uptake, and serve as imaging biomarkers—the current work highlights one such potential platform.

To address limitations associated with current anti-HER2 radioimmunoconjugates incorporating the intact antibody, antibody fragments, or formulated from larger-sized (>10 nm) particle-based probes, we combined site-specific engineered anti-HER2 scFv fragments with a clinically translated and renally clearable aminated ultrasmall silica nanoparticle^28^ via a highly specific click chemistry conjugation strategy. Thiol-maleimide conjugation is a standard method for conjugating antibodies (or fragments) to nanoparticles^37,38^ or generating antibody–drug conjugates (ADCs)^43^. However, undesirable aggregation as a consequence of disulfide bond formation and particle crosslinking have posed significant challenges to formulating the chemistry, manufacturing, and control (CMC) steps, in turn, jeopardizing translational and commercialization potential. To overcome these issues, and unlike our prior surface functionalization strategies with simple peptides^28,44^, we implemented a multi-step surface functionalization strategy to quantitatively conjugate radiometal chelators (i.e., DFO), click chemistry functional groups (i.e., DBCO), and site-specifically engineered anti-HER2 scFv (i.e., scFv-azide) fragments to yield a precisely controlled and scalable next-generation ultrasmall aminated particle. Single azide moieties containing nnAA were genetically encoded into anti-HER2 scFv fragments to enable highly facile and biorthogonal surface conjugation to DBCO-functionalized C’ dots without particle crosslinking^32^. The site-specific nature of the conjugation produced a homogenous conjugate that preserved scFv function and was resistant to uncoupling.

To ensure bulk renal clearance and low non-specific RES accumulation for clinical applications, the surface density of each attached functional group, especially DBCO and scFv, was tuned to yield a final product size of 7.3 nm or less. Importantly, ~7.3 nm C’ dot radioimmunoconjugates showed substantially lower liver uptake (~5 %ID g^−1^ vs >10 %ID g^−1^ for ^89^Zr-DFO-Trastuzumab^45^) and renal uptake (<2 %ID g^−1^ vs >200 %ID g^−1^of ^68^Ga-affibody^46^) than previous constructs, in addition to estimated whole-body excretion values of 70 %ID over a 72-h period. In contrast to the delayed PET imaging acquisition times (i.e., >3 days) required for ^89^Zr-labeled, HER2-targeting antibodies to ensure sufficient probe accumulation at the target site, adequate clearance from the plasma, and high tumor-to-background ratios^47^. These ~7.3 nm HER2-targeting C’ dots demonstrated significantly enhanced tumor-targeted uptake (13.2 ± 2.9 %ID g^−1^, *n* = 5) and T/M ratios (11.4 ± 4.6) in xenografted mice within a much shorter p.i. imaging acquisition window (i.e., 4–24 h). Compared with smaller particle sizes (i.e., 6.4 nm), however, no significant change was seen in overall tumor uptake, liver uptake, or T/L ratios (Supplementary Figs. 31 & 33). Since free ^89^Zr is a bone seeker^48^, shorter imaging acquisition delays also prevent possible false-positive interpretations of late non-tumor-associated deposition of free ^89^Zr in bone. Due to the shorter blood circulation half-life (~15 h), low non-specific RES accumulation, and bulk renal clearance of ^89^Zr-DFO-scFv-PEG-Cy5-C’ dots, the effective whole-body dose (i.e., ~0.2 mSv per MBq) was only about one-third of ^89^Zr-DFO-Trastuzumab^45^. In addition to breast cancer, in vivo HER2 imaging with ^89^Zr-DFO-scFv-PEG-Cy5-C’ dots can be used to assess other HER2-expressing tumors, including ovarian, renal, colon, and non-small cell lung cancers^49^.

C’ dot radioimmunoconjugates offer superior tumor-targeting efficiency, tumor-to-background ratios, and distinctly favorable pharmacokinetic and clearance profiles over previous constructs, overcoming key technical and translational challenges. Compared to the majority of larger-size particle probes which demonstrate substantial non-specific localization, and depend almost entirely on an enhanced permeability and retention [EPR] effect for delivery into leaky tumors^50^ or on targeting tumor neovasculature^51^, active tumor cell targeting, predominantly exhibited by our particle radioimmunoconjugate, relies upon specific cellular binding and receptor-mediated internalization of agents or toxic payloads at sites of disease following their systemic injection, extravasation, and navigation of biological barriers^52^. Tumor cell targeting continues to remain one of the key challenges of nanomedicine^53^. After more than 30 years of development, the majority of tumor cell targeting particles^36^, having a >10 nm HD demonstrate only enhanced in vitro targeting. Rarely do such particle probes exhibit significantly greater uptake in solid tumors than their non-targeting counterparts^21,36,54^. Although there is an incomplete understanding of particle extravasation, diffusion, and internalization within tumors^52^, it is generally accepted that smaller-sized nanoparticles exhibit increased tissue diffusion properties and deeper and more uniform tissue penetration^55^.

In this work, ultrasmall anti-HER2 fluorescent core–shell silica nanoparticles were not only designed for enhanced accumulation and retention at the target site, but also for improved target tissue penetration and diffusion to ultimately improve treatment efficacy after delivering toxic payloads. As shown in Figs. 3, 5, when compared with the two negative control groups, the targeted group showed at least 200–300% enhancement in BT-474 xenografted model, in terms of absolute tumor accumulation, as well as significantly improved tumor tissue penetration and distribution (Fig. 5a, g). Similar results were observed in the BT-474 orthotopic model (Supplementary Fig. 32). These observations directly challenge traditional findings with larger particle probes, namely that their physicochemical properties markedly limit penetration, diffusion, and enhanced accumulation within solid tumors, predominantly driven by the EPR effect^21,56,57^. Taken together, the foregoing data demonstrate that we have successfully overcome critical in vivo barriers to targeted delivery, penetration, specific accumulation, and retention of nanoimmunoconjugates in HER2+ breast cancer xenograft/orthotopic models using a renally-clearable particle imaging platform. The successful design and demonstration of such a clinically-promising, ultrasmall nanoimmunoconjugate for detection of HER2-expressing breast cancers serves as the foundation for staging, risk stratification, treatment planning, and next-stage targeted therapeutic developments.

## Methods

### Construction of anti-HER2 scFv and Ctr/scFv

The scFv directed against HER2/neu was designed based on Trastuzumab in a VL–VH orientation and generated by gene synthesis. A G_4_S_1_ linker was used as a spacer between VH and VL. Amber stop codons were introduced in frame at preselected sites of the LC (N-terminal and LC100) and HC (C-terminal and HC44) to designate the sites for nnAA incorporation. A control scFv was generated from a parental antibody directed against human metapneumovirus^58^. The scFv was constructed in a VL–VH orientation using gene synthesis and incorporating an intragenic amber stop codon at position HC44. The gene was cloned into a proprietary vector useful for stable expression in mammalian cells.

### Expression of scFvs containing AzK

All scFv constructs were cloned into a proprietary vector under control of the human CMV promoter. The vector encodes the gene for a glutamine synthase selectable marker. ScFv-expressing stable pools were generated by Amaxa nucleofection (Lonza, Walkersville, MD) of Chinese hamster ovary (CHO) platform cells, engineered to express the pylRS and tRNApyl^32^. Transfected cells were selected in CD CHO medium (ThermoFisher Scientific) supplemented with methionine sulfoximine (Sigma-Aldrich) in 24-well plates and grown at 37 °C and 6% CO_2_. Surviving cells were screened for scFv expression by fed-batch fermentations in 24 deep well plates in a shaking incubator (ATR Biotech) in the presence of 2 mM AzK and under the following conditions: 37 °C, 6% CO_2_, 140 rpm, and 80% relative humidity. The best producing pools were selected for scale-up and material generation using MedImmune proprietary production medium and feed regimens in the presence of 2 mM AzK. scFv titers were quantified by biolayer interferometry using protein A (N-terminal, HC44, and LC100) or anti-His (C-terminal) functionalized sensors (Pall ForteBio). More details about the purification, PEGylation and binding kinetics of scFvs can be found in the Supplementary Methods Section.

### Synthesis of DFO-scFv-PEG-Cy5-C’ dots

The DFO-scFv-PEG-Cy5-C’ dots (HD: 7.3 nm) were prepared using a multi-step approach. As a first step, aminated C’ dots, referred to as PEG-NH_2_-Cy5-C’ dots, were synthesized^29,30^. More specifically, tetramethyl orthosilicate (TMOS) and silane-functionalized Cy5 fluorescent dye were added to an ammonium hydroxide solution at pH around 8.5 at room temperature (RT) under vigorous stirring (600 rpm). One day later, (3-aminopropyl)trimethoxysilane (APTMS) and monofunctional PEG-silane with molar mass around 500 (6–9 ethylene glycol units) were added into the reaction in sequence at RT under vigorous stirring (600 rpm). The reaction mixture was further left at RT under vigorous stirring (600 rpm) overnight, and then left at 80 °C without stirring overnight. The synthesized PEG-NH_2_-Cy5-C’ dots were collected after the reaction solution cooled down to room temperature. After GPC purification, PEG-NH_2_-Cy5-C’ dots were transferred to DI water via spin filtration, and then subjected to FCS measurements for the determination of particle size and concentration^29,30^.

In the next step, PEG-NH_2_-Cy5-C’ dots were diluted into phosphate-buffered saline (PBS) (pH 7.4) buffer solution. DBCO-PEG_4_-NHS ester (in DMSO) was then added to the reaction mixture, and reacted under shaking (640 rpm) at RT for 1 h. DBCO surface density could be controlled by altering the reaction ratio between PEG-NH_2_-Cy5-C’ dots and DBCO-PEG4-NHS ester^30^. Then, DFO-NCS (in DMSO) was added, and the reaction pH value was adjusted to 8–9 in order to promote surface conjugation of DFO to C’ dots (reaction time was 2 h). A reaction ratio between PEG-NH_2_-Cy5-C’ dots and DFO-NCS of 1:20 was used to conjugate at least 3–4 DFO per C’ dot^30,59^. As-synthesized DFO-DBCO-PEG-Cy5-C’ dots were then purified by passing particles through a PD-10 column with PBS as the mobile phase to remove unreacted DBCO and DFO molecules.

To attach scFv fragments, 2.5 nmol of azide-containing scFv was added into 100 μL PBS solution of DFO-DBCO-PEG-Cy5-C’ dots (5 μM). The number of scFv fragments per particle could be precisely tuned by changing the reaction ratio or the concentration of DFO-DBCO-PEG-Cy5-C’ dots used. The mixture was continuously shaken at RT for 24 h. Free scFv fragments were removed by GPC purification. Purified DFO-scFv-PEG-Cy5-C’ dot immunoconjugates were then suspended in PBS for flow cytometry and ^89^Zr radiolabeling studies.

For the synthesis of DFO-scFv-PEG-Cy5-C’ dots with a HD of 6.4 nm, 5.8 nm sized DBCO-PEG-Cy5-C’ dots were first synthesized using the same protocol as described above, but with modifications to achieve smaller particles as described earlier^30^ (Supplementary Fig. 29), followed by functionalization with DFO and scFv fragments, described above (Supplementary Fig. 30a). More details about the Dynamic Light Scattering of scFv, synthesis and quantification of DFO-DBCO-PEG-Cy5-C’ dots, synthesis, purification and quantification of scFv-488 can be found in the Supplementary Methods Section.

### ^89^Zr radiolabeling of DFO-scFv-PEG-Cy5-C’ dots

For ^89^Zr labeling, 0.75 nmol of DFO-scFv-PEG-Cy5-C’ dots (or DFO-Ctr/scFv-PEG-Cy5-C’ dots) were mixed with 1 mCi of ^89^Zr-oxalate in HEPES buffer (pH 8) at 37 °C for 60 min; final labeling pH was kept at 7–7.5^59^. An EDTA challenge process was introduced to remove any non-specifically bound ^89^Zr by incubating the mixture at 37 °C for 30–60 min. The final ^89^Zr labeling yield was in the range of 70–80% (*n* > 5). As-synthesized ^89^Zr-DFO-scFv-PEG-Cy5-C’ dots (or ^89^Zr-DFO-Ctr/scFv-PEG-Cy5-C’ dots) were then purified by using a PD-10 column. The final radiochemical purity was estimated to be greater than 99% (measured by using Radio-TLC). The specific activity was found to be ~1000 Ci mmol^−1^. More details about the ^89^Zr-oxalate production, in vivo radio-stability studies, and dosimetry can be found in the Supplementary Methods Section.

### Cells and cell culture

Human BT-474 and MDA-MB-231 cell lines were obtained from American Type Culture Collection (ATCC). Cells were maintained in Roswell Park Memorial Institute media (RPMI 1640), supplemented with 10% fetal calf serum and 1% penicillin/streptomycin (Media Preparation Facility, Memorial Sloan Kettering).

### Xenograft and orthotopic breast cancer models

All animal studies were performed in accordance with protocols approved by the MSKCC Institutional Animal Care and Use Committee, and conformed to NIH guidelines for animal welfare. For inoculation of BT-474 xenografts, 6–8-week old female nude mice (Abico) were implanted with a 17β-estradiol pellet (1.7 mg per pellet, 90-day release, Fisher Scientific) in the left flank using a 10-gauge trocar 3 days prior to tumor implantation. For implantation, 1 × 10^7^ viable BT-474 cells were injected subcutaneously into the right flank in 100 μL of a 1:1 mixture of PBS and Matrigel (Corning, NY). For orthotopic tumor generation, 4 × 10^6^ *luc*-transfected BT-474 cells were implanted into the left mammary fat pad (#4) (Supplementary Fig. 32a). Tumor growth was monitored by bioluminescence imaging using an IVIS Spectrum small animal imaging system. MDA-MB-231 xenografts were generated in an analogous manner in the absence of 17β-estradiol pellet implantation.

### In vitro cell and competitive binding studies

HER2 receptor cell binding studies were performed by incubating 1.5 × 10^5^ cells per tube with the respective Alexa Fluor azide 488-labeled scFv fragment (scFv-488) or with DFO-scFv-PEG-Cy5-C’ dots for 1 h at 4 °C. Triplicate samples were centrifuged and washed using flow buffer prior to analysis on a BD LSRFortessa flow cytometer (BD Biosciences, San Jose, CA). Results were displayed as median fluorescence intensities using Prism 7 software (GraphPad).

Competitive binding studies were also performed by co-incubating fixed concentrations of scFv-488 (40 nM) with BT-474 cells and adding increasing concentrations of DFO-scFv-PEG-Cy5-C’ dots. Briefly, triplicate samples (1.5 × 10^5^ cells per tube) were incubated with appropriate concentrations of scFv-488 and DFO-scFv-PEG-Cy5-C’ dots for 1 h at 4 °C. Following incubation, tubes were centrifuged and cells were washed 3 times in cold flow cytometry buffer. Samples were then analyzed using flow cytometry and median fluorescent intensities were collected. MFI values were plotted against the log-transformed DFO-scFv-PEG-Cy5-C’ dot concentrations, and IC_50_ values were calculated using nonlinear regression analysis performed by Prism 7 software. IC_50_ values for the free scFv fragment were computed in a similar manner, however increasing concentrations of scFv fragment were used to compete against the scFv-488 fragment (concentration was kept the same at 40 nM). More details about the confocal microscopy can be found in the Supplementary Methods Section.

### In vivo PET and ex vivo biodistribution studies

For in vivo static PET imaging, xenografted mice (*n* = 5, ~6–7 mm diameter) were i.v.-injected with 200–300 μCi (7.4–11.1 MBq) ^89^Zr-DFO-scFv-PEG-Cy5-C’ dots (or ^89^Zr-DFO-Ctr/scFv-PEG-Cy5-C’ dots). Approximately 5 min prior to PET acquisition, mice were anesthetized by inhalation of 2% isoflurane (Baxter Healthcare, Deerfield, IL)/oxygen gas mixture, and placed on the scanner bed; anesthesia was maintained using 1% isoflurane/gas mixture. Orthotopic BT-474 tumor-bearing mice (~4 mm diameter) were i.v.-injected with 200–300 μCi (7.4–11.1 MBq) of either the ~6.4 nm radiolabeled targeted (*n* = 3) or control probe (*n* = 3).

PET imaging was performed in a small-animal PET scanner (Focus 120 microPET; Concorde Microsystems, or Inveon PET/CT, Siemens) at 2, 24, 48, and 72 h p.i. An energy window of 350–700 keV and a coincidence timing window of 6 ns were used. Data were sorted into 2D histograms by Fourier rebinning, and transverse images were reconstructed by filtered back-projection into a 128 × 128 × 63 (0.72 × 0.72 × 1.3 mm^3^) matrix. The PET imaging data were normalized to correct for non-uniformity of response, dead-time count losses, positron branching ratio, and physical decay to the time of injection; no attenuation, scatter, or partial-volume averaging corrections were applied. The counting rates in the reconstructed images were converted to activity concentrations (percentage injected dose per gram of tissue, %ID g^−1^) by use of a system calibration factor derived from the imaging of a mouse-sized water-equivalent phantom containing ^89^Zr. Region-of-interest (ROI) analyses of the PET data were performed using Inveon Research Workplace (IRW) software, with results presented as %ID g^−1^ values. At 72 h p.i., mice were sacrificed after PET scanning, and tumor and major organs harvested for ex vivo radioassay. Mouse organs were wet-weighted, counted in a Wizard^2^ γ-Counter (PerkinElmer), and converted to %ID g^−1^ (mean ± SD). More details about the fluorescence microscopy, HER2 immunofluorescence staining and digital autoradiography can be found in the Supplementary Methods Section.

### Single-dose toxicity study

Toxicity testing was performed in two groups of athymic nu/nu mice (6–8 weeks old) at doses ~5-fold greater than used for PET imaging studies. The treatment group (*n* = 3 females) received i.v.-injected targeted particles at a dose of ~2 nmol per animal of non-radiolabeled DFO-scFv-PEG-Cy5-C’ dots, while untreated controls (*n* = 3 females) received saline vehicle in a single i.v. injection (200 μL). Mice were observed daily for 3 days p.i. for signs of morbidity/mortality and weight changes. Gross necropsy, histopathology, and blood sampling for hematology and serum chemistry evaluation were performed at 3 days p.i. More details about the cell proliferation and viability of DFO-scFv-PEG-Cy5-C’ dots can be found in the Supplementary Methods Section.

### Statistics

Data were analyzed using analysis of variance for comparison across all three groups. The two degrees-of-freedom *F*-test was followed by pairwise *t*-tests that were adjusted for multiple comparisons using the Holm method; these analyses identified pairs of groups that were significantly different from one another.

## Electronic supplementary material

Supplementary Information

Peer Review File

Supplementary Movie 1

Description of Additional Supplementary Files

## Data Availability

The data that support the findings of this study are available from the corresponding authors upon reasonable request.
