# Supplementary material for: Ultrasmall targeted nanoparticles with engineered antibody fragments for imaging detection of HER2-overexpressing breast cancer
Source: Nat Commun. 2018 Oct 8;9:4141. doi: 10.1038/s41467-018-06271-5 (PMC6175906; doi:10.1038/s41467-018-06271-5)
Supplement: Supplementary file 1 — Supplementary Information [file 41467_2018_6271_MOESM1_ESM.pdf]

Supporting Information

Ultrasmall Targeted Nanoparticles with Engineered Antibody Fragments for Imaging Detection of HER2-overexpressing Breast Cancer

Feng Chen,[1,†] Kai Ma,[2,†] Brian Madajewski,[1] Li Zhuang,[3] Li Zhang,[1] Keith Rickert,[3] Marcello Marelli,[3] Barney Yoo,[1] Melik Z. Turker,[2] Michael Overholtzer,[4,5] Thomas P. Quinn,[6] Mithat Gonen,[7] Pat Zanzonico,[8] Anthony Tuesca,[3] Michael A. Bowen,[3] Larry Norton,[9] J. Anand Subramony,[3,] Ulrich Wiesner,[2]* Michelle S. Bradbury,[1,10,]**

[1]Department of Radiology, Sloan Kettering Institute for Cancer Research, New York, NY 10065, USA
[2]Department of Materials Science & Engineering, Cornell University, Ithaca, NY 14853, USA
[3]MedImmune, LLC, One MedImmune Way, Gaithersburg, Maryland 20878, United States
[4]Cell Biology Program, Sloan Kettering Institute for Cancer Research, New York, NY 10065, USA
[5]BCMB Allied Program, Weill Cornell Medical College, New York, NY 10065, USA
[6]Department of Biochemistry, University of Missouri, Columbia, MO 65211, USA and Harry S Truman Veterans' Hospital, Columbia, MO 65201 USA
[7]Department of Epidemiology and Biostatistics, Sloan Kettering Institute for Cancer Research, New York, NY 10065, USA
[8]Department of Medical Physics, Sloan Kettering Institute for Cancer Research, New York, NY 10065, USA
[9]Department of Medicine & Office of the President, Memorial Sloan Kettering Cancer Center, New York, NY 10065, USA
[10]Molecular Pharmacology Program, Sloan Kettering Institute for Cancer Research, New York, NY 10065, USA
[†]F.C. and K.M. contributed equally to the work. *M.B., U.W. and A. S. contributed equally to the work.

Correspondence and requests for materials should be addressed to:
M.S. Bradbury, email: bradburm@mskcc.org, U. Wiesner, email: ubw1@cornell.edu and A. Subramony, email: subramonya@MedImmune.com

Section 1. Supplementary Methods

Purification of scFvs. Expressed scFvs were purified from culture medium using protein A affinity chromatography. To enable the purification of the scFv fragment containing a nnAA at the C-terminus, a 6xHis tag was encoded downstream of the amber codon; this affinity tag was expressed upon successful nnAA incorporation, and enabled the purification of nnAA-containing scFv via metal affinity resins. The 6xHis tag also contained a thrombin cleavage site to allow for removal of the tag after its purification. Protein A bound scFvs were eluted with 0.1M glycine pH3.1 and rapidly neutralized with 1M Tris pH 7.5. Peak fractions were dialyzed to PBS pH 7.2 overnight at 4 °C. Removal of aggregates was performed using preparatory SEC chromatography. Samples were concentrated to 3-5 mg/mL in PBS.

PEGylation of the anti-HER2 scFvs. 50 μg of azide-containing scFv were incubated overnight with 5-fold molar excess of 20 kDa-PEG-DBCO (Click Chemistry Tools, Scottsdale AZ) or PEG4-DBCO (MilliporeSigma, St.Louis MO), in 20 μl of PBS containing 10% DMSO at room temperature with end over end mixing. At the end of the incubation the reaction mixture was prepared for SDS-PAGE analysis without purification. 2-3 μg of scFv was resolved under non-reducing conditions and proteins stained with Coomassie Blue.

Binding kinetics. Binding kinetics for the anti-HER2 scFvs were measured using surface plasmon resonance. Octet (Forte Bio) NiNTA sensors were incubated with 200 nM HER2-ECD-6xHis for 10 mins to generate Her-ECD-NI-NTA biosensors. After about 1 min equilibration in binding buffer (PBS) the HER2-ECD sensors were incubated with a titration of anti-HER2 scFv or PEGylated anti-HER2 scFv (ranging from 500nM to 7.8nM) for 5 min, followed by dissociation for 20 min. Octet Analysis software (V9.0; Pall) was used for curve fitting, and calculation of KD values.

Dynamic Light scattering of scFv. The hydrodynamic radius of the scFv was examined by size exclusion chromatography and multi-angle light scattering (SEC-MALS). For analysis of the anti-Her2 scFv, 50-100ug sample was injected into an Agilent 1260 HPLC, onto a TSK-gel G2000, 7.8x300 mm, running at 1 mL/minute in a buffer of 0.1 M sodium phosphate and sodium sulfate at pH 6.8. UV280 signal was measured with the Agilent UV detector, while refractive index and light scattering were measured on a Wyatt t-Rex and Dawn Heleos-2 instrument, respectively. Hydrodynamic radius was calculated from the mean of multiple light scattering measurements across the top of the eluted peak on the Dawn Heleos-2. Molar mass analysis was performed within Astra v7 software, using refractive index detector data for the analysis.

Synthesis and quantification of DFO-DBCO-PEG-Cy5-C' dots. C' dots conjugated with DBCO and DFO functional groups were synthesized as follows: NH_2-PEG-C' dots (100 µL, 15 µM, suspended in DI water) were first added to 200 µL of phosphate-buffered saline (PBS) (pH 7.4), mixed with DBCO-PEG4-NHS ester (in DMSO), and reacted under shaking (640 rpm) at room temperature for 1 hour. DBCO surface density could be easily controlled by altering the reaction ratio between NH_2-PEG-C' dots and DBCO-PEG4-NHS ester. For example, approximately 7 DBCO per C' dot could be achieved using a 1:25 molar ratio; the number increased to 11-12 while increasing the ratio to 1:100. Without purification, DFO-NCS (in DMSO) was then added, adjusting the pH value to 8-9 in order to promote surface conjugation of DFO to C' dots (reaction time was 2 h). The reaction ratio between NH_2-PEG-C' dots and DFO-NCS was then maintained at 1:20 in order to conjugate 3-4 DFO per C' dot.[1, 2] As synthesized DFO-DBCO-PEG-Cy5-C' dots were purified by passing the particles through a PD-10 column using PBS as the mobile phase.

To quantify the number of accessible DBCO per C' dot, DFO-DBCO-PEG-Cy5-C' dots (200 µL, 1 µM, in PBS) were reacted with an azide-functionalized green dye, Alexa Fluor azide 488 (i.e., azide-488) using a reaction (molar) ratio of 1:20. As-synthesized DFO-DBCO-PEG-C' dot-azide-488 was subsequently purified using a PD-10 column to remove un-reacted free azide-488. The concentrations of both C' dots and azide-488 from DFO-DBCO-PEG-C' dot-azide-488 could be estimated on the basis of calibration curves. Due to the high specific reaction between DBCO and azide groups, the density of accessible DBCO should be equal to the number of azide-488, and can be calculated using the simple equation below. By quantifying the depletion of accessible DBCO before and after the conjugation of scFv fragments, we could obtain the scFv ligand density.

$$\text{Number of azide 488 per C}'\text{dot} = \frac{\text{Concentration of azide 488 in DFO} - \text{DBCO} - \text{PEG} - \text{Cy5} - \text{C}'\text{dot}}{\text{Concentration of C}'\text{dot in DFO} - \text{DBCO} - \text{PEG} - \text{Cy5} - \text{C}'\text{dot}}$$

The numbers of functional ligands per particle were also quantified using a spectra-based approach.[2] More specifically, the UV-Vis spectrum of DFO-scFv-PEG-Cy5-C' dots was deconvoluted into the individual contributions of each component via a fitting procedure. The equation used for the fit was a linear combination of the absorption intensity profiles of different functional ligands, including Cy5, DBCO, DFO and scFv fragments. The concentrations for each component were then calculated using their extinction coefficients, respectively. The numbers of functional ligands per particle were finally estimated by dividing the ligand concentrations, *i.e.* Cy5, DBCO, DFO and scFv, obtained from UV-Vis absorbance deconvolution, by the particle concentrations, obtained from fluorescence correlation spectroscopy (FCS) measurements, respectively.[3]

Synthesis, purification and quantification of scFv-488. Green dye conjugated scFv fragments were synthesized for (1) testing HER2 targeting capability of scFv fragments before conjugating to C' dots and (2) determining IC_{50} of both the free scFv and DFO-scFv-PEG-Cy5-C' dots. To synthesize these conjugates, 26 µL (or 4 nmol) of scFv-azide (3.71 mg/mL, or 0.15 nmol/µL) was mixed with 50 µL of PBS, then added to 20 nmol (2 µL, 10 nmol/µL) carboxyrhodamine 110 DBCO (or DBCO-488) and shaked (640 rpm) at room temperature overnight. As-synthesized scFv-488 was then purified using a PD-10 column to remove free DBCO-488 (see PD-10 elution profiles of both the scFv-488 and free DBCO-488, **Supplementary Fig. 16c**). Each scFv-488 conjugate was comprised, on average, of 0.6 green dye molecules. The final concentration of scFv-488 was found to be about 2.5 nmol/mL.

Confocal microscopy. BT-474 and MDA-MB-231 cell lines were plated in a 24-well plate at a density of 5,000 cells per well using poly-D-lysine (P-7886, Sigma Aldrich, St. Louis, MO) coated coverslips, and allowed to attach overnight. Cells were subsequently treated with 100 nM particle solutions (Her2-scFv-488, Ctr-scFv-488, Her2-scFv-C' dot, or Ctr-scFv-C' dots) in RPMI 1640 supplemented with 10% FBS for 4 hours at 37°C. Following incubation, cells were washed twice with PBS, and then fixed for 5 min with 1% paraformaldehyde. Cells were washed multiple times with PBS after a 5 min treatment with a 0.05 mg/ml DAPI solution (D1306, ThermoFisher Scientific, Waltham, MA), as well as prior to mounting coverslips using ProLong Gold mounting media (P36930, ThermoFisher Scientific). Image capture was performed using a Leica-SP8 point-scanning confocal microscope (Leica Microsystems, Buffalo Grove, IL). Displayed composite and single channel images were extracted from projections created using Imaris software (Bitplane, South Windsor, CT).

Cell Proliferation and Viability of DFO-scFv-PEG-Cy5-C' dots. MDA-MB-231 and BT-474 cells were first plated at a concentration of 1×10^4 cells per well in an opaque 96-well plate in triplicate. Cells were attached overnight, followed by treatment with DFO-scFv-PEG-Cy5-C' dot particles in RPMI media containing 10% FBS or normal media alone. Viability assays evaluated concentrations of DFO-scFv-PEG-Cy5-C' dot ranging from 25-100 nM at both 24 hr and 48 hr time points. The percentage of viable cells was calculated using the CellTiter-Glo (Promega, Madison, WI) manufacturer's instructions. For proliferation assays, a DFO-scFv-PEG-Cy5-C' dot concentration of 100 nM was evaluated over a 96-hour exposure time. Cell numbers were acquired every 24 hours using the CellTiter-Glo assay mentioned above. To calculate the proliferation ratio, luminescence values at each time point were divided by the initial (0-hour) time point and displayed using Prism 7 software (GraphPad).

^{89}Zr-oxalate production. ^{89}Zr was produced at Memorial Sloan Kettering Cancer Center on a TR19/9 cyclotron (Ebco Industries Inc.) *via* the ^{89}Y(p,n)^{89}Zr reaction and purified to yield ^{89}Zr with a specific

activity of 5.28-13.43 mCi/µg (470-1195 Ci/mmol) of zirconium.[4] Activity measurements were performed using a CRC-15R Dose Calibrator (Capintec). For the quantification of activities, experimental samples were counted on an Automatic Wizard[2] γ-Counter (PerkinElmer). All *in vivo* experiments were performed according to protocols approved by the Memorial Sloan Kettering Institutional Animal Care and Use Committee. A purity of greater than 95% was confirmed using radio-TLC for all of the ^{89}Zr-labeled DFO-scFv-PEG-Cy5-C' dots.

In vivo radio-stability studies. For *in vivo* radio-stability, healthy mice were injected with ~200 µCi (~7.4 MBq) of ^{89}Zr-DFO-scFv-PEG-Cy5-C' dots. Whole blood was collected at 2, 24 and 48 h post-injection, and the plasma fraction was isolated from red blood cells by centrifugation at 8000 rpm for 10 min. The percentage of the intact ^{89}Zr-DFO-scFv-PEG-Cy5-C' dots was then measured by using Radio-TLC with the plates analyzed on a Bioscan AR-2000 radio-TLC plate reader using Winscan Radio-TLC software (Bioscan Inc., Washington, DC).

Dosimetry. Time-activity curves derived for each tissue were analytically integrated, accounting for radioactive decay, to yield the corresponding cumulative activity. Organ absorbed doses were then calculated by multiplying the cumulative activity by the ^{89}Zr equilibrium dose constant for non-penetrating radiations (positrons), assuming complete local absorption of such radiations and ignoring the contribution of penetrating radiations (*i.e.,* γ-rays). Mouse normal organ cumulated activities were converted to human normal organ cumulated activities by taking into account differences in total-body and organ masses between mice and humans (assuming 70-kg standard human). Calculated human normal-organ cumulated activities were entered into the OLINDA dosimetry program to compute standard human organ absorbed doses using formalism of the Medical Internal Dosimetry Committee of the Society of Nuclear Medicine.[5] This human dosimetry model is a "normal" (*i.e.,* tumor-free) anatomic model.

Fluorescence microscopy. Harvested BT-474 and MDA-MB-231 tumors, mounted in Tissue-Tek O.C.T, were sectioned at a thickness of 10 µm on an Avantik Cryostatic Microtome (Avantik Biogroup, Springfield Township, NJ). H&E staining of these tumor specimens was performed. Images were captured on a BX60 fluorescence microscope (Olympus America Inc., Center Valley, PA), equipped with a motorized stage (Prior Scientific Instruments Ltd., Rockland, MA) and CC12 camera (Olympus) at 10X magnification. Whole tumor section images were obtained by acquiring multiple fields that were then stitched together using MicroSuite Biologic Suite (version 2.7, Olympus). Brightfield (H&E) and fluorescence (Cy5) images were acquired using the appropriate filter cube sets. Processing of fluorescence images was carried out in Adobe Photoshop (CS6), as described previously.[6]

HER2 immunofluorescence staining. Frozen sections were air dried for 30 min at room temperature, followed by fixation in 3% paraformaldehyde for 15 min at room temperature in a humidified chamber. Sections were washed twice with buffer (1X TBS/0.1% Tween-20) for 5 min. Endogenous peroxides were blocked using BLOXALL (SP-6000, Vector Labs, Burlingame, CA) for 10 min, and tissue sections were subsequently washed twice with buffer for 5 min, prior to the addition of the blocking solution (5% Normal Goat Serum/wash buffer). Blocking solution was incubated with sections for 1 hour at room temperature, followed by washing and the addition of HER2 (#2165S, Cell Signaling, Danvers, MA) primary antibody (1:400 dilution) in blocking solution. Sections were then incubated overnight at 4°C in a humidified chamber, followed by multiple washes and addition of FITC conjugated anti-rabbit secondary antibody (Cell Signaling, #4412) at a dilution of 1:300 in blocking solution. Tissue sections were incubated at room temperature for 30 min, and washed in triplicate for 5 min. Nuclear counterstaining was performed with DAPI (0.1 mg/ml). Sections were washed twice for 5 min, followed by coverslip application using Prolong Gold Antifade (ThermoFisher, #P10144) mounting media. Images were captured using identical acquisition settings and subjected to synonymous post-processing procedures across all specimens. Image acquisition was performed in an analogous manner to the methods described above.

Digital Autoradiography. 10 µm tumor tissue sections were exposed to a phosphor-imaging plate (Fujifilm BAS-MS2325, Fuji Photo Film, Japan) at -20°C. Following exposure, the phosphor-imaging plate was read at a resolution of 25 µm using a Typhoon 7000 IP (GE Life Sciences, Pittsburgh, PA) plate reader. Images were saved as TIFF and GEL formats for future analysis and processed using ImageJ software (https://imagej.nih.gov/ij/).

Section 2. Supplementary Discussion

2.1. Purification of anti-HER2 scFv fragments demonstrates high monomeric content. All affinity purified scFv fragments, except C-terminal scFv, showed a discrete band by SDS-PAGE and Coomassie staining (**Fig. 1c**). The scFv containing the nnAA at the C-terminus showed several higher molecular mass bands migrating at regular intervals. Resolution of this protein under reducing conditions eliminated these bands, suggesting formation of disulfide-linked multimers in this sample (data not shown). The doublet bands in the C-terminal sample represent 6xHis tagged and truncated versions of the scFv fragments that migrate at slightly different molar masses. Size exclusion chromatography (SEC) analysis of non-reduced products was performed to examine the monomeric state of the samples before the aggregate removal described above (**Supplementary Fig. 1**). Consistent with our observations on SDS-PAGE, N-terminal and HC44 showed high monomeric content with low aggregates detected, while LC100 and C-terminal constructs showed a secondary species (50 kDa) that may have represented scFv dimer. Successful incorporation of azide-containing nnAA into the scFv fragments was further demonstrated by reacting nnAA-expressing scFvs with 20-kDa PEG molecules functionalized with dibenzocyclooctyne (DBCO), and analyzed by using mass spectrometric and antigen binding capability (**Supplementary Figs. 2-4, Supplementary Table 1,** *Supporting Information, Section 2.1*). Based on the foregoing results, anti-HER2 scFvs containing the nnAA at HC44 were selected for subsequent C' dot bioconjugation studies.

2.2. Efficient PEG Conjugation and Product Analysis. To confirm successful incorporation of N6-((2-azidoethoxy)carbonyl)-L-lysine (**AzK**) into the scFv fragments, nnAA-bearing scFvs were conjugated to 20-kDa PEG molecules functionalized with DBCO. The DBCO moiety provides a strained alkyne group that reacts with an azide to form a triazole linkage. The resulting adduct increases the mass of the molecule, resulting in a retardation of gel mobility in SDS-PAGE analyses. As shown in **Supplementary Fig. 2**, scFv fragments and scFv-PEG conjugates were resolved by SDS-PAGE and stained with Coomassie Blue; a clear increase in molecular mass was observed in the PEGylated samples. To further confirm the specificity of conjugate formation, mass spectrometric analysis of modified scFvs was conducted. The scFv fragments of the HC and N-terminal constructs were reacted with a five-fold molar excess of DBCO-PEG_4 increasing the mass of the adducts by 838 Da. Unmodified and PEGylated samples were analyzed by liquid chromatography–mass spectrometry (LC-MS) and the intact ion mass of the proteins measured (**Supplementary Fig. 3**). In both cases, we observed an 838 Da increase in the overall mass of the respective PEGylated scFv fragments. No unreacted material was observed in any of the PEGylated samples, indicating very efficient conjugation.

Another consideration for selecting a conjugation site is whether scFv binding function would be impaired by formation of the adduct. Both free and PEGylated scFv fragments were assessed for antigen-binding capability. While PEG chains cannot reproduce the possible influence a conjugated nanoparticle might have on the binding affinity of scFvs, the addition of a 20-kDa flexible PEG identified sites that might interfere with the antigen binding domains. Kinetic binding analyses were performed to assess on- and off-rates of the scFvs and PEGylated scFvs to the ECD of HER2 (**Supplementary Fig. 4**). The data showed that the N-terminal and HC44 sites resulted in a slight reduction of the equilibrium dissociation constant (K_D) relative to the unmodified scFv (**Supplementary Table 1**). This is likely due to steric inhibition from the long flexible PEG molecule that interferes with the antigen binding domains of the scFv. The dynamics of the PEG conjugate in the context of a nanoparticle will be different due to the constrained nature of the PEG in the C-dots. The data presented indicate that the site of conjugation offers a viable attachment site that preserves the function of the scFv. The C-terminal site scFv, which contains a 6xHis tag, was not measurable in this assay as NiNTA sensors were used for the HER2 receptor capture.

2.3. Surface DBCO and scFv density optimization. Post surface engineering plays a vital role in developing multifunctional nanoplatforms for cancer diagnosis and treatment.[2, 7] Although PEGylation may prevent aggregation of ultrasmall C' dots during surface modification[2, 8] and transport in the bloodstream - a key step to ensure bulk renal clearance in animal models and human subjects[9, 10] - endowing the less than 7 nm sized fluorescent PEG-NH_2-Cy5-C' dots with two additional functions (PET imaging and targeting) via four additional chemical moieties (i.e., DBCO, DFO, scFv and ^{89}Zr) of varying molar mass (*i.e.,* 0.6 kDa to 25 kDa) to improve active tumor targeting while maintaining bulk renal clearance and low RES uptake is challenging.

Different reaction ratios (i.e., 1:25, 1:50 and 1:100) between PEG-NH_2-Cy5-C' dot and DBCO-PEG4-NHS ester were explored resulting in accessible DBCO numbers per particle in the range of 6 to 12, as shown in **Supplementary Fig. 8**. Quantification of number of accessible DBCOs per particle was achieved by reacting DFO-DBCO-PEG-Cy5-C' dots with an excess amount of azide-functionalized green dye, *i.e.*, carboxyrhodamine 110 alkyne (or azide-488) as schematically shown in **Supplementary Fig. 9a**, followed by quantification of the number of azide-488 conjugated to a DFO-DBCO-PEG-Cy5-C' dot. **Supplementary Figs. 9b-e** shows the PD-10 elution profiles of as-synthesized azide-488-DFO-DBCO-PEG-Cy5-C' dot based on the emission intensities of both C' dot (*Ex: 651 nm, Em: 675 nm*) and azide-488 (*Ex: 501 nm, Em: 525 nm*). The number of azide-488 dye per C' dot was then calculated based on estimated concentrations of azide-488 dye and C' dots. The number of accessible DBCO molecules per C' dot should equal that of azide-488 dye per C' dot due to

high yields of the specific strain-promoted azide-alkyne cycloaddition (SPAAC) reaction between DBCO and azide groups. Free unreacted azide-488 was separated from the azide-488-DFO-DBCO-PEG-Cy5-C' dot as demonstrated in **Supplementary Fig. 10**, which shows the two distinguishable elution profiles of free azide-488 dye and PEG-NH_2-Cy5-C' dots. Increasing numbers of azide-488/DBCO per dot were further evidenced by the increased absorbance of azide-488 at 501 nm (**Supplementary Fig. 11**). Preliminary biodistribution studies were performed to check the reticuloendothelial system (RES) uptake of ^{89}Zr-labeled DFO-DBCO-PEG-Cy5-C' dots with varying DBCO densities before attaching the 25-kDa scFv fragments. As shown in **Supplementary Fig. 12**, ^{89}Zr-DFO-DBCO-PEG-Cy5-C' dots with less than 10 accessible DBCO per C' dot (HD: ≤7.0 nm) exhibited very similar biodistribution profiles, noting only minimal liver (<5 %ID/g, 24h p.i.) and spleen (<3 %ID/g, 24h p.i.) uptake. By contrast, C' dots bearing ~12 accessible DBCO (HD: 7.5 nm) demonstrated mildly elevated RES uptake, likely a consequence of their increasing size, thereby making them less suitable for further bioconjugation with scFv fragments.

Next, we studied the controlled bioconjugation of anti-HER2 scFv-azide to DFO-DBCO-PEG-Cy5-C' dots via SPAAC reaction (**Supplementary Fig. 13a**). Our results showed that the number of scFv per C' dot could be controlled from <1 to ~2 by altering the reaction ratio between DFO-DBCO-PEG-Cy5-C' dots and scFv-azide (from 1:1 to 1:20, **Supplementary Fig. 13b**), by changing the reaction concentration of DFO-DBCO-PEG-Cy5-C' dot (from 1 μM to 10 μM, **Supplementary Fig. 13c**) or by using DFO-DBCO-PEG-Cy5-C' dot with varying accessible DBCO surface densities (ranging from 7-12 DBCO molecules per C' dot, **Supplementary Fig. 13d**). A preliminary biodistribution study of DFO-scFv-PEG-Cy5-C' dots with varying numbers of scFv fragments per C' dot was also performed after radiolabeling with ^{89}Zr and i.v. injection into healthy mice at 24 h post injection. For DFO-scFv-PEG-Cy5-C' dots with 2 scFv per C' dot, the data in **Supplementary Fig. 14** shows a significant increase of ^{89}Zr-DFO-scFv-PEG-Cy5-C' dot uptake in the liver and spleen (*i.e.*, close to or higher than 7 %ID/g) due to an ~8 nm HD. Nanoconjugates bearing 0.8 and 1.3 scFv per particle exhibited relatively smaller hydrodynamic diameters (i.e., 6.7 and 7.3 nm, respectively), with only minimal liver (~5 %ID/g) and spleen (<3 %ID/g) uptake. Based on these results, DFO-scFv-PEG-Cy5-C' dots with an HD of ~7.3 were selected for in-depth immunoconjugate characterization, *in vitro* HER2 active targeting studies, radiolabeling, and *in vivo* HER2 targeted PET imaging studies.

2.4. FCS measurement results of dye-conjugated scFv (i.e., scFv-488) and C' dot immunoconjugates. The hydrodynamic size of scFv was measured by FCS (**Supplementary Fig. 15b**), and results compared to DLS measurement results (**Supplementary Fig. 15a**). In order to endow scFv with fluorescence signal for FCS measurements, a green fluorescent dye, i.e. carboxyrhodamine

110 DBCO (or DBCO-488), was first conjugated to the scFv via DBCO-azide reaction. The average hydrodynamic diameter of the dye conjugated scFv-488 was determined to be around 3.8 nm by FCS (**Supplementary Fig. 15b**), and slightly larger than results from DLS measurements without the dye (**Supplementary Fig. 15a**).

In order to rationalize the "apparent" smaller than expected increase of particle size upon fragment attachment, it is important to emphasize that DLS and FCS techniques only provide "effective" particle HDs that correspond to the diffusive properties of an assumed perfectly spherical particle. Considering that the HD of the base aminated particle, onto which the scFvs were attached, was 6.6 nm, a diameter increase of 0.7 nm after scFv attachment corresponds to a more than 30% increase in particle volume. Since the particle molar mass is roughly around 100 kDa,[11] this substantial particle volume increase is consistent with the molar mass of the scFv, even though the particle size increase seems minor. Another way of thinking about this result is to picture the diffusive properties of the particle-fragment conjugate as that of an anisotropic ellipsoidal object (rather than a perfect sphere). Even though this object is elongated in one direction (e.g. the particle-fragment axis), its resistance to flow is most likely governed by it cross-sectional area along the ellipsoidal short axis which essentially still is that of the base particle, consistent with our experimental measurements.

2.5. GPC Purification of DFO-scFv-PEG-Cy5-C' dots. We originally tried to use a PD-10 column to remove unreacted scFv-azidefragments from DFO-scFv-PEG-Cy5-C' dots after the fragment attachment step. However, purification was not successful, maybe due to the fact that both samples were eluted from 2.5 mL to 4.0 mL fractions. To illustrate this result, a green dye conjugated scFv fragment was prepared by reacting scFv-azide with carboxyrhodamine 110 DBCO (or DBCO-488) to form scFv-488 (**Supplementary Figs. 16a-b**). The elution profile, shown in **Supplementary Fig. 16c**, confirmed a clean separation of scFv-488 from the free un-reacted DBCO-488. In contrast, overlapping fractions (i.e., from 2.5 mL to 4.0 mL) were found between free scFv-488 fragment and PEG-NH_2-Cy5-C' dots (**Supplementary Fig. 17**), resulting in inefficient purification of DFO-scFv-PEG-Cy5-C' dots using a PD-10 column.

To ensure clean separation, a GPC column purification process was used.[11] The GPC elugram of unpurified DFO-scFv-PEG-Cy5-C' dots exhibited three peaks (**Supplementary Fig. 6c**). A peak at around 9 mins corresponded to the main particle product (**Supplementary Fig. 6c**), while a peak at about 5 min could be attributed to impurities with large molar mass that can usually be removed by GPC. A peak at around 12 to 13 min elution time could be shown to correspond to free azide-functionalized scFv (**Supplementary Fig. 6c** bottom), revealing unreacted azide-scFv. By collecting

the elution volume between about 8 and 11 min, all impurities could be successfully removed, resulting in a GPC elugram with a single peak (**Supplementary Fig. 6d**) and suggesting high product purity.

2.6. Quantification of surface functional group density by UV-Vis. The numbers of DBCO and scFv per C' dot were further quantified using an indirect approach based on the conjugation efficacy of DBCO groups. As compared to the quantification based on UV-Vis spectrophotometry, which detects all the DBCO moieties on C' dot surface, the conjugation efficacy approach is only sensitive to the DBCO groups that are reactive. As a result, while 22 DBCO groups were identified using the spectrophotometry approach, the number of accessible DBCO moieties per C' dot was estimated to be about 7 using the conjugation efficacy approach (**Supplementary Table 2**). This difference suggests that a considerable fraction of the DBCO groups on the C' dot surface is not accessible, and therefore cannot react with azide-functionalized molecules. This may be due to DBCO groups folding back onto the (microporous) silica surface under the PEG layer. In contrast, measurements of the number of scFv fragments per particle exhibited high consistency between the two qualification methods (**Supplementary Table 2**). This is expected as scFv could only react with accessible DBCO groups, and the quantification of scFv is therefore not affected by the percentage of non-accessible DBCO. Considering that the number of accessible DBCO ligands is more meaningful than the overall ligand number, in the remainder of this paper we will exclusively refer to the former obtained using the conjugation efficacy approach when describing DBCO ligand numbers.

2.7. Radiolabeling strategies and in vivo biodistribution studies. To label DFO-scFv-PEG-Cy5-C' dots with ^{89}Zr for *in vivo* bioditribution studies, DFO-scFv-PEG-Cy5-C' dots were mixed with ^{89}Zr-oxalate at pH 7-8 (37 ºC) to form ^{89}Zr-DFO-scFv-PEG-Cy5-C' dots. As-synthesized ^{89}Zr-DFO-scFv-PEG-Cy5-C' dots were then purified to achieve a final radiochemical purity of >99% by radio-TLC (**Supplementary Fig. 25**). The average specific activity of ^{89}Zr-DFO-scFv-PEG-Cy5-C' dots was estimated to be ~1000 Ci/mmol. In our prior work,[1] chelator-based ^{89}Zr-labeled C' dots demonstrated remarkably high (>99%) in vitro radiostability in human serum over a 120-hour time interval.[1] The non-radioactive cRGDY-PEG-Cy5-C' dots have an extended 2-year shelf-life, revealed by long-term GPC and FCS monitoring.[12] In this study, in vivo radiostability of ^{89}Zr-DFO-scFv-PEG-C' dots was evaluated in non-tumor-bearing nude mice following their intravenous (i.v.)-injection with ~200 μCi (~7.4 MBq) ^{89}Zr-DFO-scFv-PEG-Cy5-C' dots. To test radiochemical purity, whole blood specimens were collected at 2, 24 and 48 hours p.i., and the plasma fraction, containing >98% of the ^{89}Zr-DFO-scFv-PEG-Cy5-C' dots, was separated from whole blood by centrifugation. Non-specific association of ^{89}Zr-labeled C' dots with red blood cells was previously estimated to be less than 2%.[1] The plasma fraction was found to contain >95% of the intact ^{89}Zr-DFO-scFv-PEG-Cy5-C' dots at 48 hours p.i. (i.e., **Supplementary**

Fig. 26a, about three blood circulation half-lives of ^{89}Zr-DFO-scFv-PEG-Cy5-C' dots), which along with FCS analysis of non-radioactive DFO-scFv-PEG-Cy5-C' dots (**Supplementary Fig. 26b**) suggested high *in vivo* and *ex vivo* radiostability, respectively.

The high *in vivo* radiostability was further confirmed on the basis of biodistribution profiles (**Supplementary Fig. 27**) demonstrating low (<1 %ID/g) ^{89}Zr radioactivity in bone specimens harvested at 72 hours p.i. ^{89}Zr-DFO-scFv-PEG-Cy5-C' dots. High ^{89}Zr intraosseous activity usually indicates detachment of ^{89}Zr from the probe, since free $^{89}Zr^{4+}$ is an osteophilic cation, which is readily accreted into bone mineral.[13] Similar to previously reported findings using peptide-functionalized radiolabeled C' dots,[9, 14, 15] particle radioimmunoconjugates, labeled with ^{89}Zr, exhibited high blood activity concentrations at early p.i. time points (**Supplementary Fig. 27)**, as well as significantly lower liver uptake (i.e., ~5 percentage injected dose (%ID)), in comparison with larger particle sizes (>10 nm) that are generally hepatically-cleared (*i.e.,* 30-99 %ID)[16], and <5% ID/g uptake for other major organs and tissues. Although ultrasmall C' dots are excreted by the kidneys,[1, 9, 10, 14, 15] measured renal activities of ^{89}Zr-DFO-scFv-PEG-Cy5-C' dots were only about 2 %ID/g over the study interval, significantly lower (i.e., ~5-10 fold less) than those previously described for other renally clearable ultrasmall particles (e.g., quantum dots[17] or gold nanoparticles[18]). This finding suggests that the particles are not retained by the kidneys. Furthermore, whole-body clearance of ^{89}Zr-DFO-scFv-PEG-Cy5-C' dots was estimated to be about 70 %ID (n>15) over a 72 hour p.i. interval.

To estimate mean organ absorbed doses and the effective dose of ^{89}Zr-DFO-scFv-PEG-C' dots in a 70-kg standard man, dosimetric analyses **(Supplementary Table 3)**[5] were performed on the basis of the biodistribution data shown in **Supplementary Fig. 27.** Overall, favorable dosimetry was found for this radioimmunoconjugate in terms of total-body dose equivalent (~0.2 mSv/MBq) and effective dose (~0.2 mSv/MBq). An dose equivalent of ~0.4 mSv/MBq was estimated for the liver, which was only one-fourth of that reported for ^{89}Zr-DFO-trastuzumab. In the latter case, the liver was found to be the dose-limiting organ, with an uptake of ~12 %ID, and an average estimated absorbed dose of 1.54 mSv/MBq.[19] These clinically promising results led to our subsequent rigorous evaluation of targeted uptake in breast cancer models with ^{89}Zr-DFO-scFv-PEG-Cy5-C' dots.

2.8. Additional *in vivo* studies were performed using newly-developed, smaller-sized targeted particles (*i.e.*, 6.4 nm) having a comparable scFv surface density to assess size-dependent changes in PK profiles and/or HER2-specific targeting and retention in (i) BT-474 xenografted mice (beyond the 72-hour window) and in (ii) an orthotopic BT-474 model. In the former case, prolonged targeted particle retention was observed up to 10 days, with T/M and T/L ratios of >20 and nearly 3, respectively (**Supplementary Fig. 31)**. Enhanced target-specific uptake (i.e., greater than 2-fold) was also observed

in orthotopic tumors using ^{89}Zr-DFO-scFv-PEG-Cy5-C' dots, in comparison to that found for control group (i.e., mice injected with isotype control scFv fragments conjugated C' dots, ^{89}Zr-DFO-Ctr/scFv-PEG-Cy5-C' dots, **Supplementary Fig. 32)**. Importantly, while the use of a smaller diameter particle modestly lowered liver uptake, it also led to alterations in other biological properties, including faster blood clearance and mildly reduced absolute tumor uptake and T/L ratios (**Supplementary Fig. 33)**.

2.8.1. In vivo **HER2-targeting of ^{89}Zr-DFO-scFv-PEG-Cy5-C' dots (HD: 6.4 nm) in xenograft BT-474 breast cancer model over a 10-day period.** Particles of smaller HD (6.4 nm) were developed for studies in *Section 2.8*. To synthesize smaller DFO-scFv-PEG-Cy5-C' dots, 5.8 nm DBCO-NH_2-PEG-Cy5-C' dots were generated using previously published methods[2] (**Supplementary Fig. 29**), followed by attachment of DFO and scFv fragments, described above (**Supplementary Fig. 30a**). As-synthesized DBCO-NH_2-PEG-Cy5-C' dots were functionalized with DFO chelator by reacting with DFO-NCS (molar ratio: 1:20) at pH 8-9 to form DFO-DBCO-PEG-Cy5-C' dots bearing 3-4 DFO moieties per C' dot. Subsequently, DFO-DBCO-PEG-Cy5-C' dots were mixed with pre-synthesized anti-HER2 scFv-azide in PBS (pH 7.4) and reacted overnight to form DFO-scFv-PEG-Cy5-C' dots. Unreacted scFv fragment was removed *via* GPC purification. The number of scFv per C' dot was estimated to be 1.5, close to that of larger sized counterparts (HD: 7.3 nm; 1.4 scFv/C' dot). For ^{89}Zr radiolabeling, DFO-scFv-PEG-Cy5-C' dots were mixed with ^{89}Zr-oxalate at pH 7-8 (37 ºC) to form ^{89}Zr-DFO-scFv-PEG-Cy5-C' dots (HD: 6.4 nm).

For extended PET imaging over a 10-day period, ^{89}Zr-DFO-scFv-PEG-Cy5-C' dots (HD: 6.4 nm) were i.v.-injected into BT-474 xenografted mice (n=3) followed by serial coronal and axial tomographic PET imaging at/on 1 h, 4 h, Day 1, Day 2, Day 3, Day 5, Day 7 and Day 10 (**Supplementary Figs. 31a-b**). Findings clearly demonstrated targeted tumor retention of the particle probe, with tumor uptake reaching a plateau at around 10 %ID/g (n=3, **Supplementary Fig. 31b**). Blood, liver, and muscle activity-concentrations decreased over time, from 17.3±2.0 %ID/g (at 1 h p.i.) to 0.7±0.1 %ID/g (on Day 10) for blood (**Supplementary Fig. 31c**), 8.4±0.3 %ID/g (at 1 h p.i.) to 3.7±0.7 %ID/g (on Day 10) for liver (**Supplementary Fig. 31d**) and 1.0±0.1 %ID/g (at 1 h p.i.) to 0.5±0.1 %ID/g (on Day 10) for muscle (**Supplementary Fig. 31d**). Due to high tumor retention of the particle probe, with concomitant clearance from other major organs/tissues, progressively increasing tumor-to-liver (0.8±0.1 to 2.8±0.4, **Supplementary Fig. 31e**), tumor-to-blood (0.4±0.1 to 14.0±1.9, **Supplementary Fig. 31f**), and tumor-to-muscle (from 6.7±0.9 to 20.5±4.4, **Supplementary Fig. 31g**) ratios were observed.

2.8.2. *In vivo* HER2-targeting of ^{89}Zr-DFO-scFv-PEG-Cy5-C' dots (HD: 6.4 nm) in orthotopic BT-474 breast cancer model. An orthotopic BT-474 breast cancer model was generated by implanting

4×10^6 Luc-transfected BT-474 cells into the left mammary fat pad (#4) (**Supplementary Fig. 32a**). Growth of the tumor was monitored by bioluminescence imaging (BLI) using an IVIS Spectrum small animal imaging system. As shown in **Supplementary Figs. 32b-c**, increasing bioluminescence signal was observed from Day 7 to Day 19 post-implantation. Average tumor size was measured by calipers to be ~4 mm on Day 19. For the targeted group (n=3), mice were i.v.-injected with 6.4 nm ^{89}Zr-DFO-scFv-PEG-Cy5-C' dots, while non-targeted group animals (n=3) were i.v.-injected with similarly sized ^{89}Zr-DFO-Ctr/scFv-PEG-Cy5-C' dots. All mice were imaged using PET over a 3-day period. **Supplementary Fig. 32d** shows coronal and axial tomographic PET imaging of mice from both groups at 48 h p.i., with >2-fold enhancement in tumor uptake for mice in the targeted group (6.4±1.1 %ID/g), as against the non-targeted group (3.0±0.6 %ID/g). A comparison of tumor uptake (**Supplementary Fig. 32e**) and tumor-to-organ ratios (**Supplementary Figs. 32f-h**) at all p.i. time points confirmed that the targeted group significantly outperformed the non-targeted group. Ex vivo biodistribution profiles at 72 h p.i. showed dominant tumor uptake for the targeted group (6.6±1.3 %ID/g), while remaining major organs demonstrated mild non-specific uptake (<5 %ID/g) (**Supplementary Fig. 32i**). The non-targeted group shared a similar normal organ uptake profile, except that for tumor (3.2±1.1 %ID/g). A strong correlation of imaging findings with ex vivo histology (i.e., H&E, fluorescence microscopy, autoradiography) of tumor specimens harvested 72 hours p.i. was noted, further confirming enhanced tumor-specific uptake for the targeted group (**Supplementary Figs. 32j-k**). Reduced tumor uptake was found for the smaller sized ^{89}Zr-DFO-scFv-PEG-Cy5-C' dots (controls), as against that seen with the larger-sized particles (6.4±1.1 %ID/g vs 13.2±2.9 %ID/g). However, orthotopic BT-474 tumor sizes were also seen to be smaller, on average, for the former group (i.e., ~4 mm), as against those measured for the latter group (i.e., ~6-7 mm). As such, reduced tumor uptake may be attributed to less neovascularization of the relatively smaller orthotopic BT-474 tumors or, perhaps reflects the use of a smaller-size particle probe that clears more rapidly (**Supplementary Fig. 33a**) and demonstrates a shorter blood circulation half-life.

2.9. Impact of smaller sized probe on *in vivo* PK. It has been generally accepted that, for renally clearable nanoparticles (e.g. quantum dots[20]), a decrease in particle size usually leads to a shorter blood circulation half-life and faster renal clearance rate. In this section, we compared differences in tumor and liver uptake for probes having two different hydrodynamic diameters. As expected, and as shown in **Supplementary Fig. 33**, decreasing the HD of ^{89}Zr-DFO-Ctr/scFv-PEG-Cy5-C' dots by about 1 nm could lead to lower blood retention times, lower liver uptake, lower tumor uptake, as well as a lower tumor-to-liver ratio. For example, the blood activity-concentration of 6.4 nm ^{89}Zr-DFO-Ctr/scFv-PEG-Cy5-C' dots was on average 25-30% less than that of the 7.3 nm sized probes. The reduction in

non-specific liver uptake was not as obvious as that seen for the blood. Larger sized ^{89}Zr-DFO-Ctr/scFv-PEG-Cy5-C' dots showed 10-20% higher BT-474 uptake and tumor-to-liver ratios. **Taken together**, our results suggested that the current design of the "target or clear" ~7.3 nm ^{89}Zr-DFO-scFv-PEG-Cy5-C' dots, bearing about 1.4 scFv fragments per particle, achieves the best balance among the biological properties measured, that is, it yields the highest HER2-specific targeting efficacy, while maintaining dominant renal clearance and low non-specific RES accumulations.

Section 3. Supplementary Figures

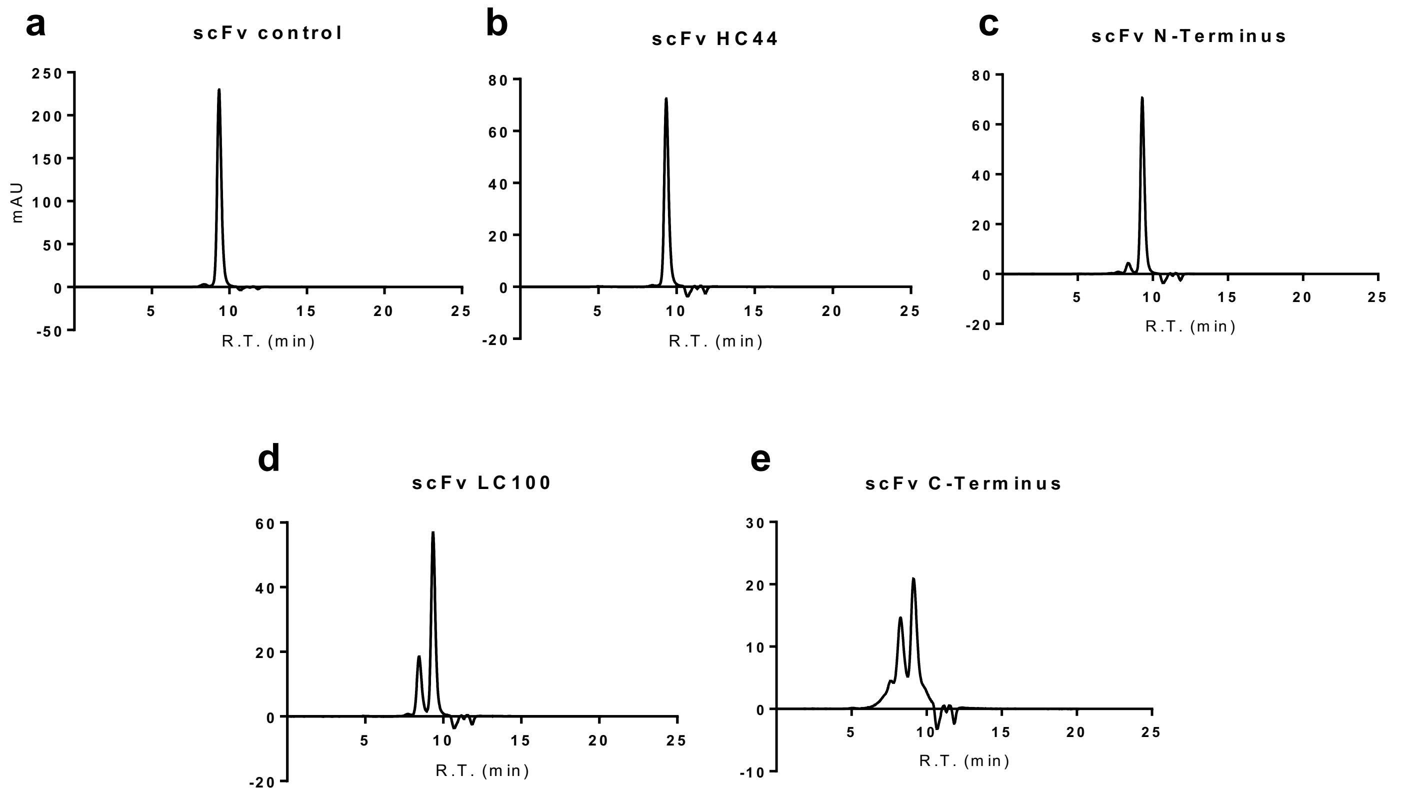

Supplementary Figure 1. SEC analysis of affinity-purified scFvs. Little aggregation was observed for (**a**) scFv control, and scFvs containing nnAA at positions (**b**) HC44 and (**c**) N-terminal. Aggregate peaks were observed in (**d**) L100 and (**e**) C-term samples. These aggregates may represent scFv dimers.

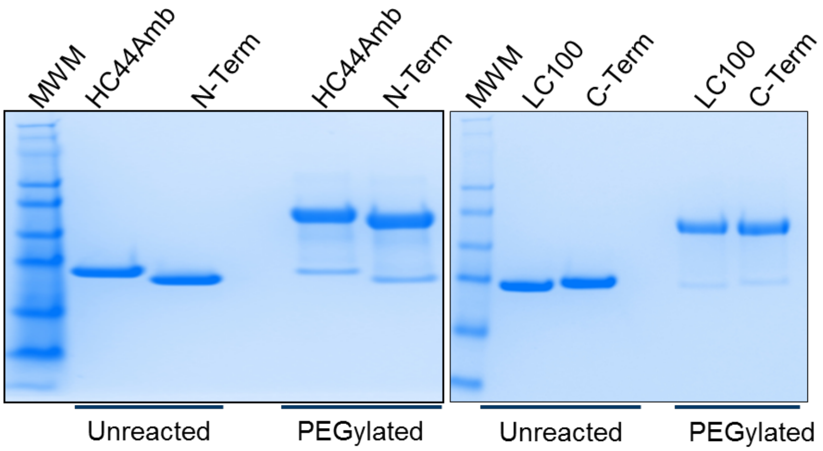

Supplementary Figure 2. Expressed scFvs are efficiently modified. scFvs with nnAA introduced at one of four different sites enables efficient conjugate formation. Expressed and purified scFvs were incubated with a 5-fold molar excess of 20 kDa-PEG-DBCO for 16 h at RT. Reactants were resolved by SDS-PAGE without purification along with their unreacted counterparts. The addition of PEG results in an increase in molar mass that is readily observed. >99% efficiency of conjugation was observed.

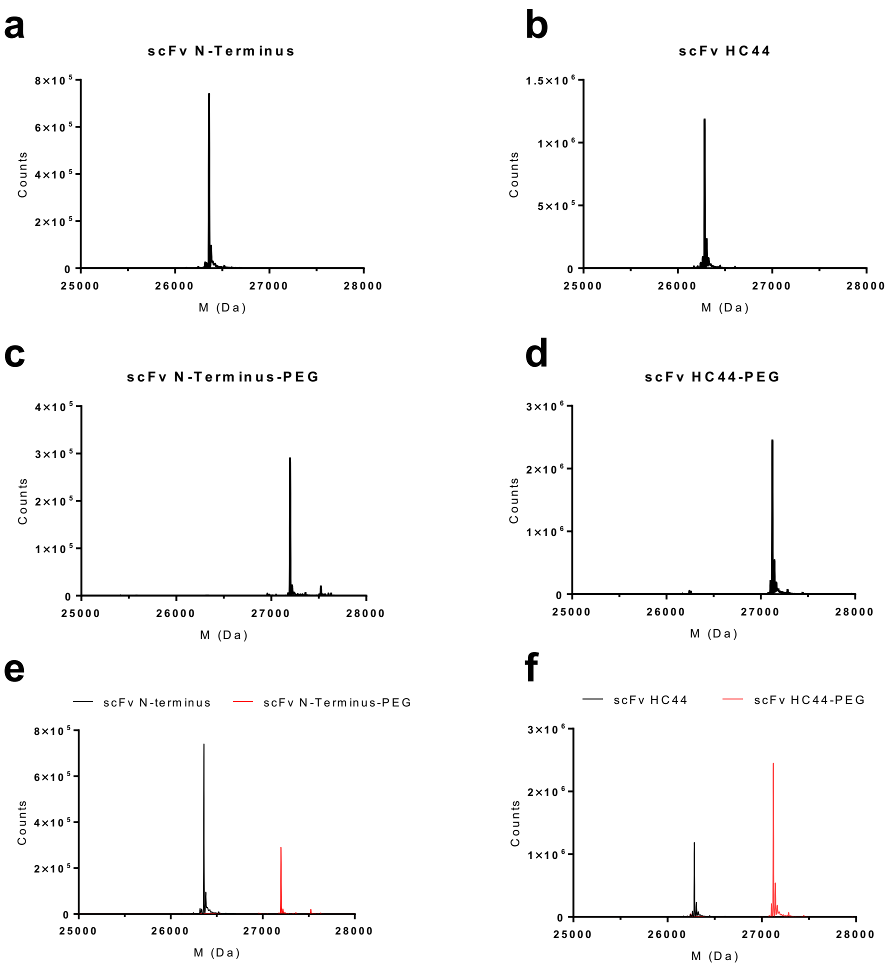

Supplementary Figure 3. Mass analysis of free scFvs (**a-b**) and PEGylated scFvs (**c-d**). Successful PEGylation of HC and N-terminal scFvs was observed by an 838 Da increase in the scFv mass (**e-f**).

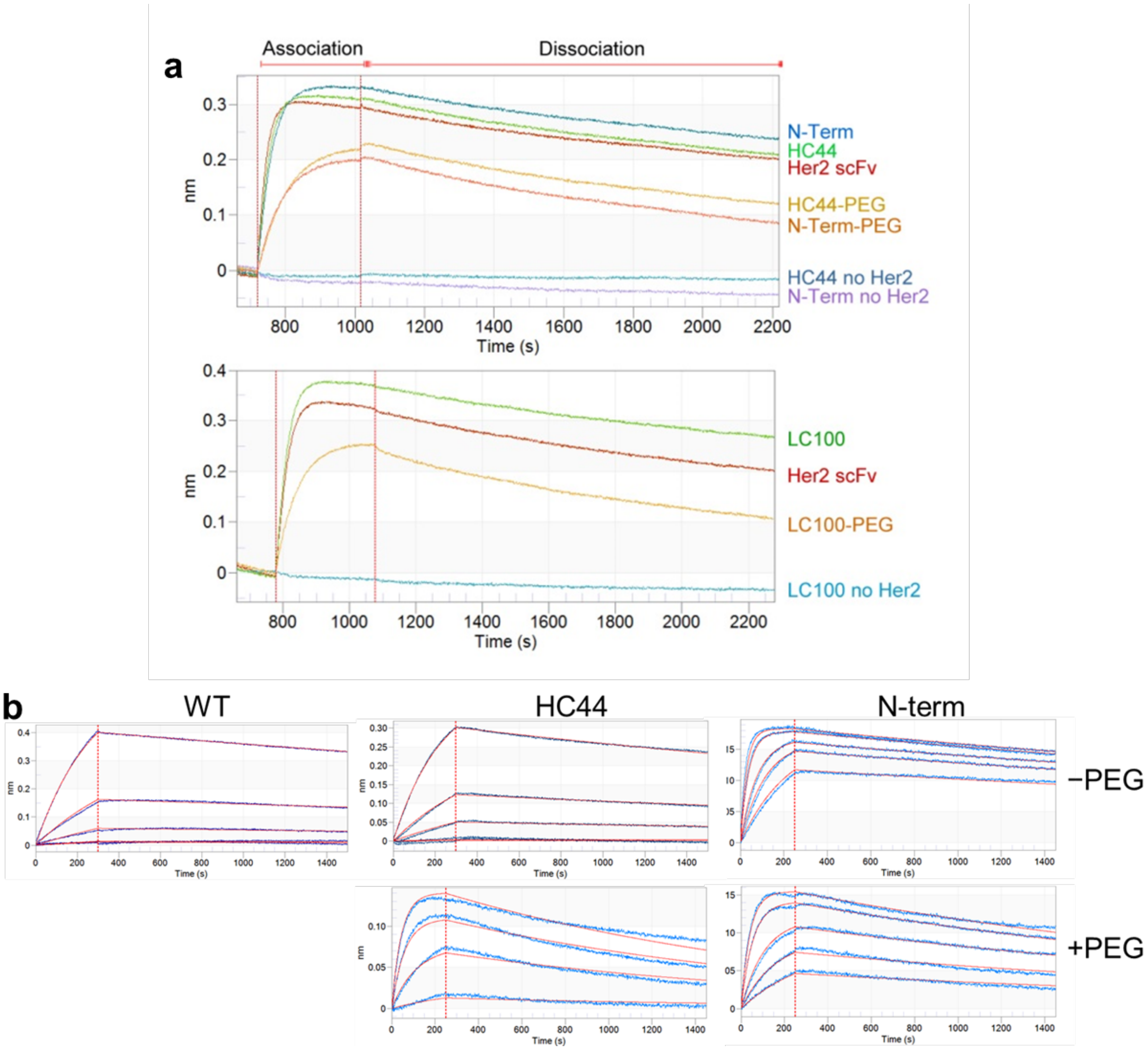

Supplementary Figure 4. Kinetic binding of scFvs to HER2 ECD. (**a**) Relative association and dissociation curves were examined for four positional variants, either unconjugated or complexed with 20kDa PEG chains. Traces were normalized after HER2 loading for comparing scFv binding kinetics. Upper plot shows binding and dissociation kinetics for N-terminal and HC44 variants alone or conjugated to PEGs. The lower plot shows binding kinetics of LC100 alone or conjugated to PEGs. No scFv binding to sensors lacking HER2 (no HER2 controls) was noted. Wildtype scFvs directed to HER2 (HER2 scFv) was examined in both studies. (**b**) Full kinetic characterization of anti-Her2 scFvs (WT, HC44 and N-term) using titrations of unmodified and PEGylated scFVs. Data analysis by a global fitting algorithm (Forte Biotech software) was used to determine association and dissociation rates (see **Supplementary Table 1**).

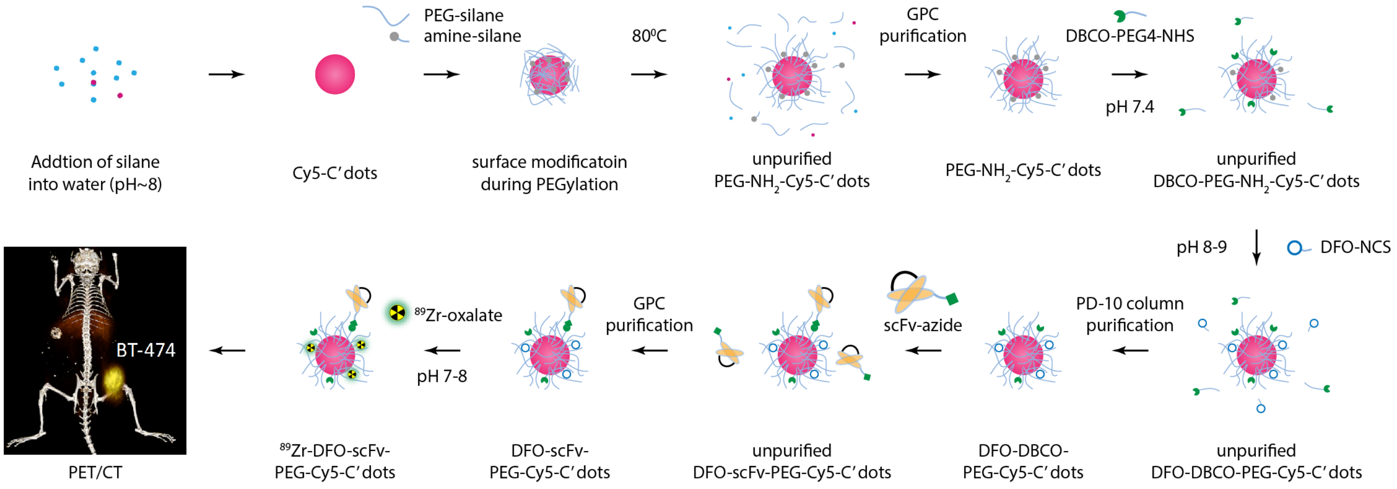

Supplementary Figure 5. Schematic illustration of the synthesis of ^{89}Zr-DFO-scFv-PEG-Cy5-C' dots. Aminated PEG-Cy5-C' dots were synthesized via co-condensing amine-silane and PEG-silane onto the Cy5-C' dot surface during PEGylation. The as-synthesized PEG-NH_2-Cy5-C' dots were GPC purified, and then reacted with DBCO-PEG4-NHS ester at pH 7.4 (molar ratio: 1 to 25) to attach DBCO to the C' dot surface via amine-NHS ester reaction, yielding DBCO-PEG-NH_2-Cy5-C' dots. Without further purification, DFO-NCS was added to the solution at pH 8-9 reacting with the remaining silica surface bound amine groups, forming DFO-DBCO-PEG-Cy5-C' dots. Unreacted DFO and DBCO were removed from the reaction mixture by passing the solution through a PD-10 column. Subsequently, DFO-DBCO-PEG-Cy5-C' dots were mixed with pre-synthesized anti-HER2 scFv-azide in PBS, and reacted overnight, forming DFO-scFv-PEG-Cy5-C' dots. Unreacted scFv fragment was removed via GPC purification. For ^{89}Zr radiolabeling, DFO-scFv-PEG-Cy5-C' dots were mixed with ^{89}Zr-oxalate at pH 7-8 (37 ºC) to form ^{89}Zr-DFO-scFv-PEG-Cy5-C' dots. Free ^{89}Zr was removed using a PD-10 column. The final ^{89}Zr-DFO-scFv-PEG-Cy5-C' dots were i.v. injected into BT-474 (HER2 positive) and MDA-MB-231 (HER2 negative) tumor-bearing mice, respectively, to assess tumor-targeted uptake and pharmacokinetic profiles of radiolabeled C' dot immunoconjugates with serial PET imaging.

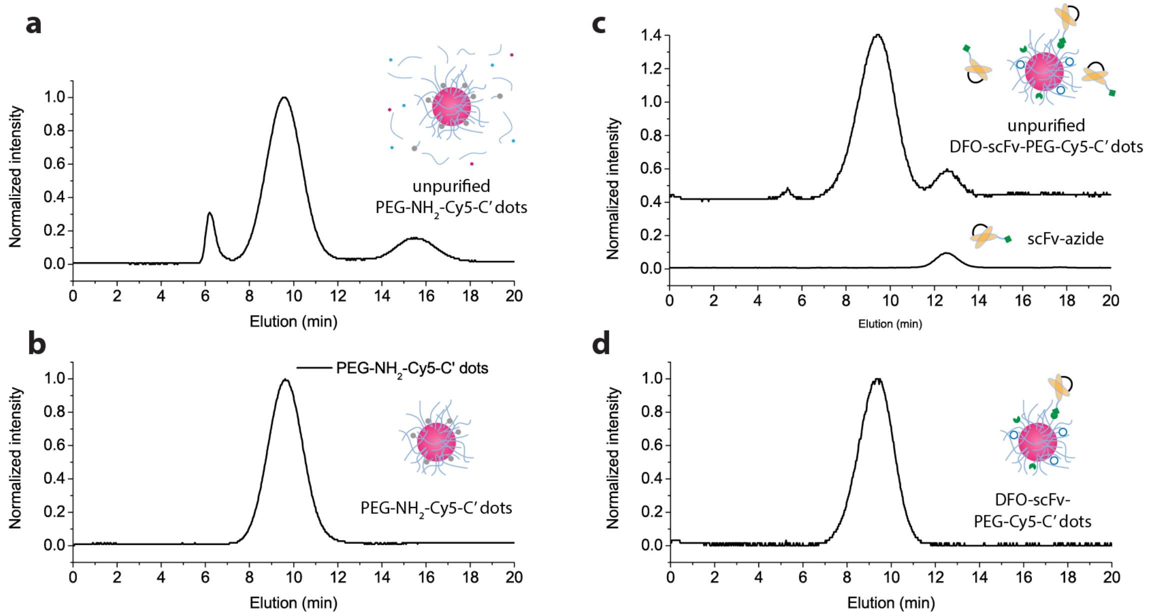

Supplementary Figure 6. GPC purifications at various steps in the synthesis of DFO-scFv-PEG-Cy5-C' dots. (**a-b**) GPC elugrams of aminated PEG-NH_2-Cy5-C' dots before (**a**) and after (**b**) GPC purification. (**c-d**) GPC elugrams of DFO-scFv-PEG-Cy5-C' dots before (**c**) and after (**d**) GPC purification. On the bottom of (c) a GPC elugram of free azide-functionalized scFv fragment is shown. Comparison of data in (c) and (d) demonstrates the effective separation of C' dots and scFv fragments using GPC.

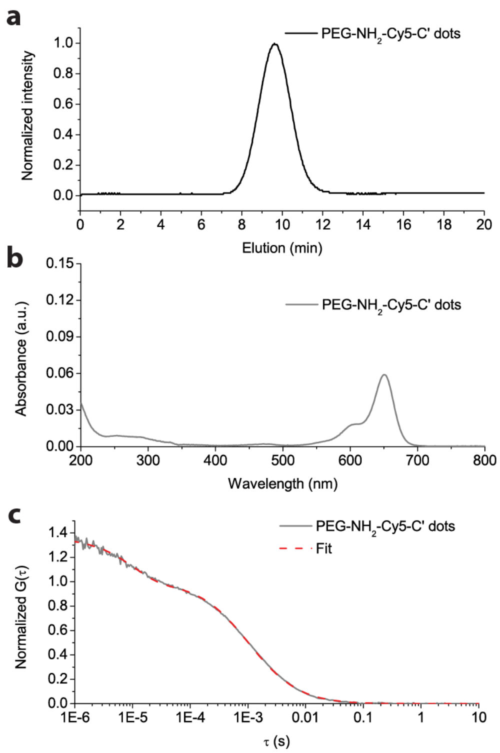

Supplementary Figure 7. Characterization of purified PEG-NH_2-Cy5-C' dots. (a) GPC elugram. (b) UV-Vis absorbance spectrum. (c) FCS correlation curve with fit. GPC runs indicated high purity of the final PEG-NH_2-Cy5-C' dot product (**7a**). The UV-Vis absorption spectrum of purified PEG-NH_2-Cy5-C' dots exhibited strong signal around 650 nm, consistent with successful Cy5 dye encapsulation (**7b**). A weak absorption signal around 480 nm suggested limited degradation of Cy5 dye caused by the amination step.

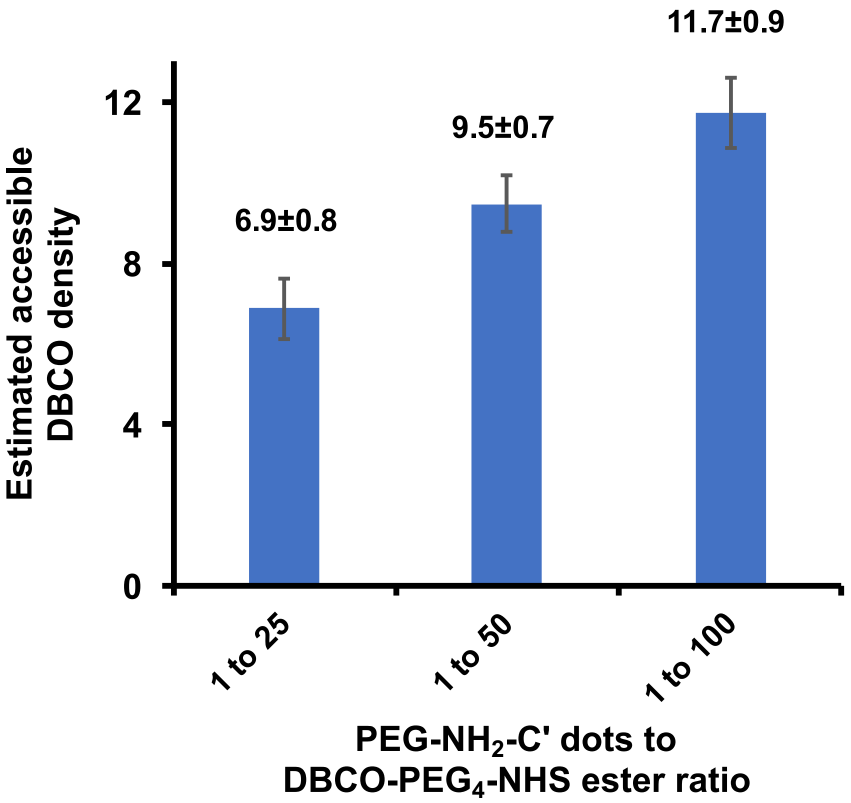

Supplementary Figure 8. Estimated number of DBCO per DFO-DBCO-PEG-Cy5-C’ dots synthesized by varying the reaction ratio (i.e., 1:25, 1:50 and 1:100) of PEG-NH_2-Cy5-C’ dots and DBCO-PEG4-NHS ester.

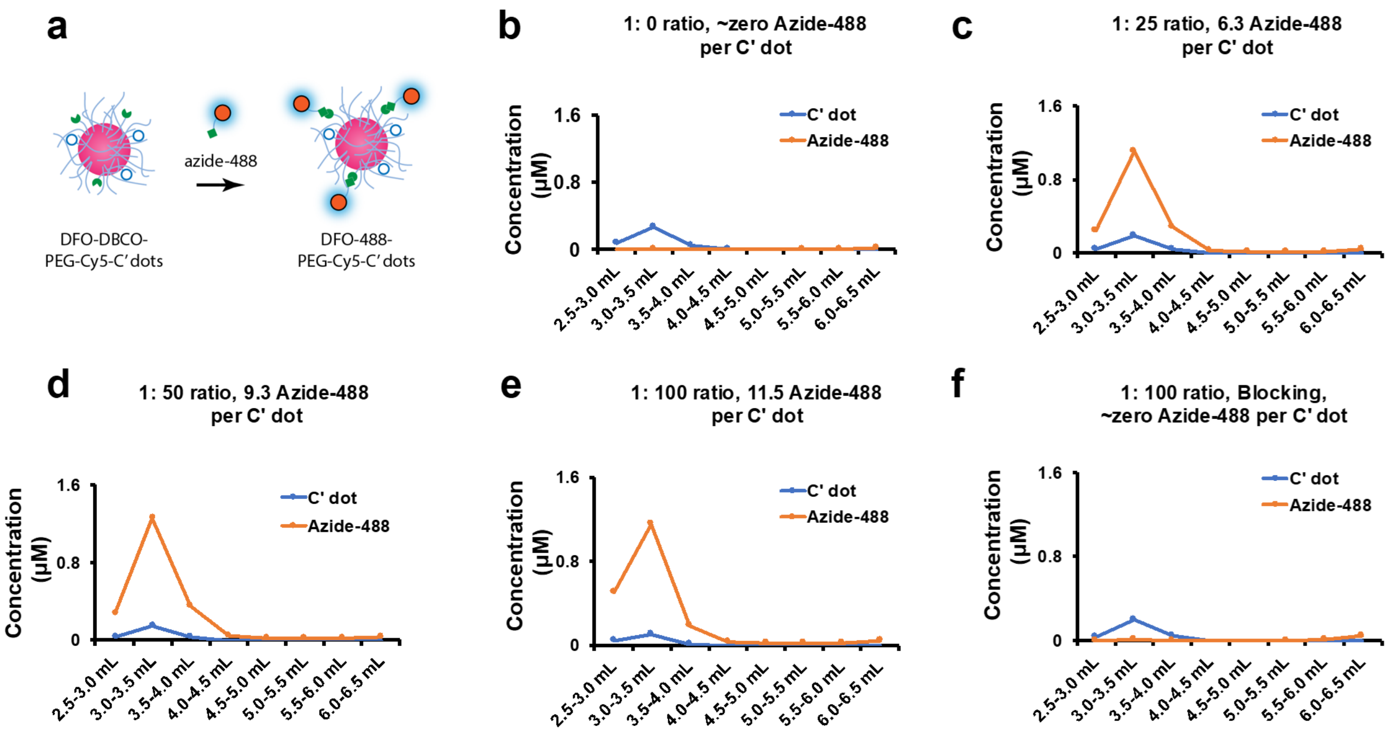

Supplementary Figure 9. Quantification of the accessible number of DBCOs per DFO-DBCO-PEG-Cy5-C' dot. (**a**) Reaction scheme between the DFO-DBCO-PEG-Cy5-C' dots and carboxyrhodamine 110 alkyne (or azide-488). The DFO-DBCO-PEG-Cy5-C' dots were synthesized using a range of reaction ratios (**b**) 1:0, (**c**) 1:25, (**d**) 1:50, and (**e**) 1:100 between PEG-NH_2-Cy5-C' dot and DBCO-PEG_4-NHS ester. Panels (**b**) to (**e**) show representative PD-10 elution profiles of different DFO-DBCO-PEG-Cy5-C' dot-azide-488 conjugates, synthesized by reacting DFO-DBCO-PEG-Cy5-C' dots with a 10-fold excess of green dye azide-488. The same elution profile was quantified using the fluorescence intensity of both the azide-488 (*Ex: 501 nm, Em: 525 nm*, orange line) and the PEG-NH_2-Cy5-C' dot (*Ex: 651 nm, Em: 675 nm*, blue line). DFO-DBCO-PEG-Cy5-C' dot-azide-488 was eluted from fraction 2.5 mL to 4.0 mL. After quantifying C' dot and conjugated azide-488 concentrations, the number of azide-488 dye per C' dot was calculated. The number of accessible DBCO per C' dot should equal that of azide-488 per C' dot as a result of high yields for the high specific strain-promoted azide-alkyne cycloaddition reaction between DBCO and azide groups. Free azide-488 can be well-separated from the azide-488-DFO-PEG-Cy5-C' dot (see elution profiles of free azide-488 and PEG-NH_2-Cy5-C' dot, **Supplementary Fig. 10**). (**f**) elution profile of DFO-DBCO-PEG-Cy5-C' dot-azide-488 after reacting DFO-DBCO-PEG-Cy5-C' dots with a 10-fold excess of azide-PEG_3 (no green dye) in order to conjugate all accessible DBCO moieties prior to reacting with azide-488 (green dye). The number of azide-488 was negligible, clearly indicating successful blocking of the reaction between DFO-DBCO-PEG-Cy5-C' dot and azide-488.

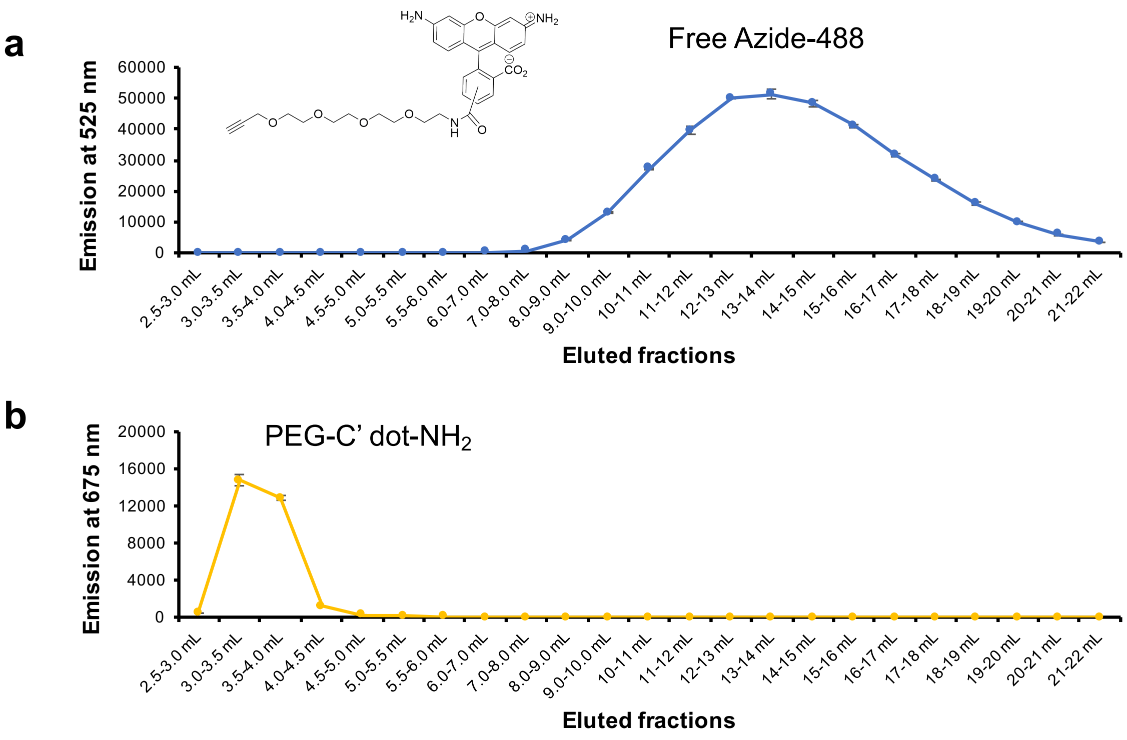

Supplementary Figure 10. PD-10 elution profiles of (**a**) free carboxyrhodamine 110 alkyne (or azide-488) and (**b**) PEG-NH_2-C’ dot. Inset in (**a**) shows the chemical structure of azide-488.

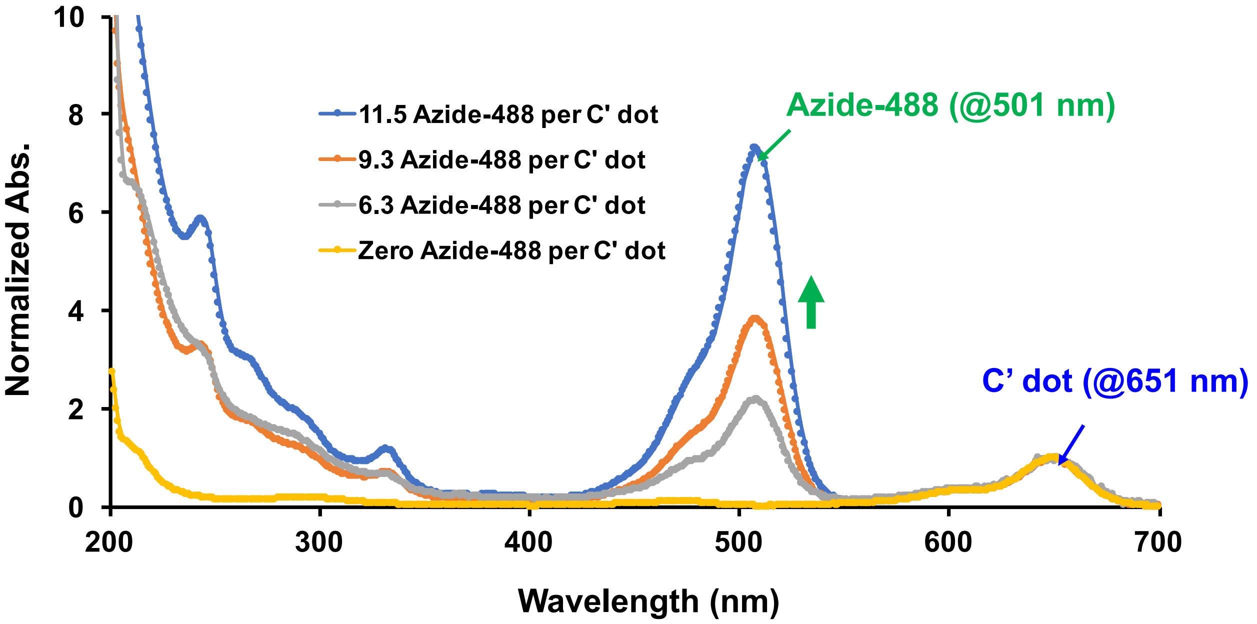

Supplementary Figure 11. UV-vis absorbance spectra of azide-488-DFO-DBCO-PEG-Cy5-C’ dots with varying numbers of azide-488, ranging from zero to 11.5, normalized to the absorbance of C’ dots at 651 nm. Clear increases in the azide-488 absorbance around 501 nm were observed (green arrow) with increasing numbers of azide-488 molecules per C’ dot.

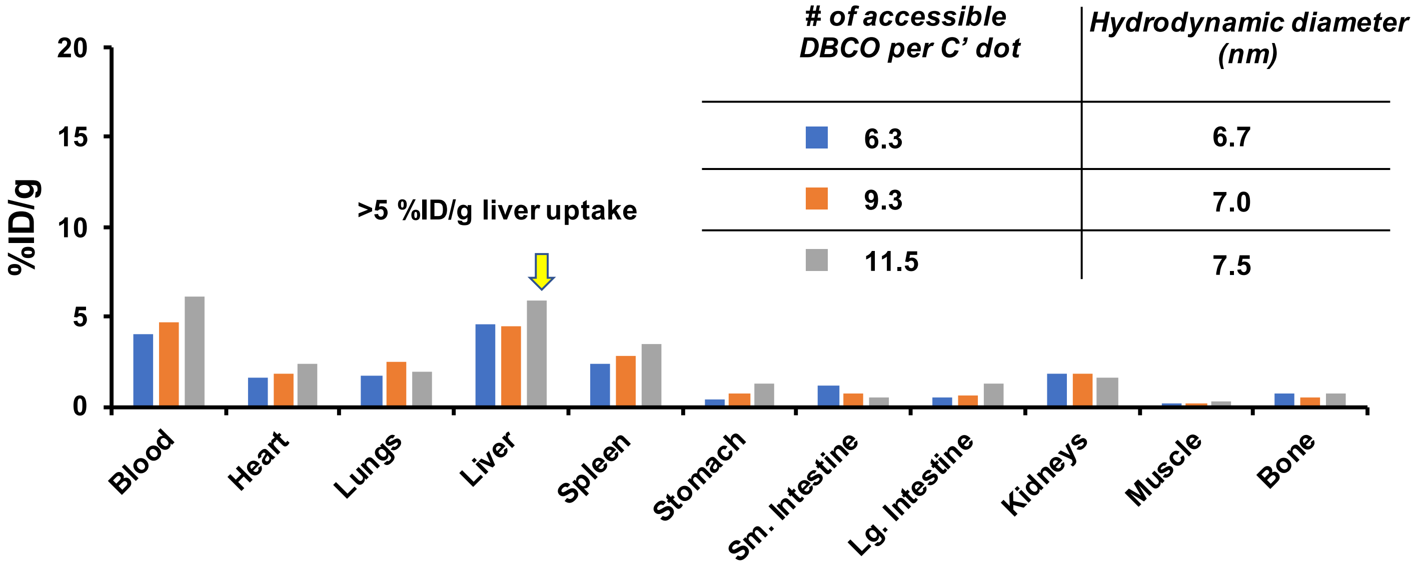

Supplementary Figure 12. Biodistributions for ^{89}Zr-DFO-DBCO-PEG-Cy5-C' dots with varying accessible DBCO numbers per dot at 24 h post injection time point (n=1). For the highest accessible DBCO number per dot of ~12 (^{89}Zr-DFO-DBCO(11.5)-PEG-Cy5-C' dots), analysis suggested a mild progressive increase in liver (>5 %ID/g) and spleen uptake with larger HDs, precluding its further use for conjugation. Inset shows the corresponding hydrodynamic diameters of the tested samples.

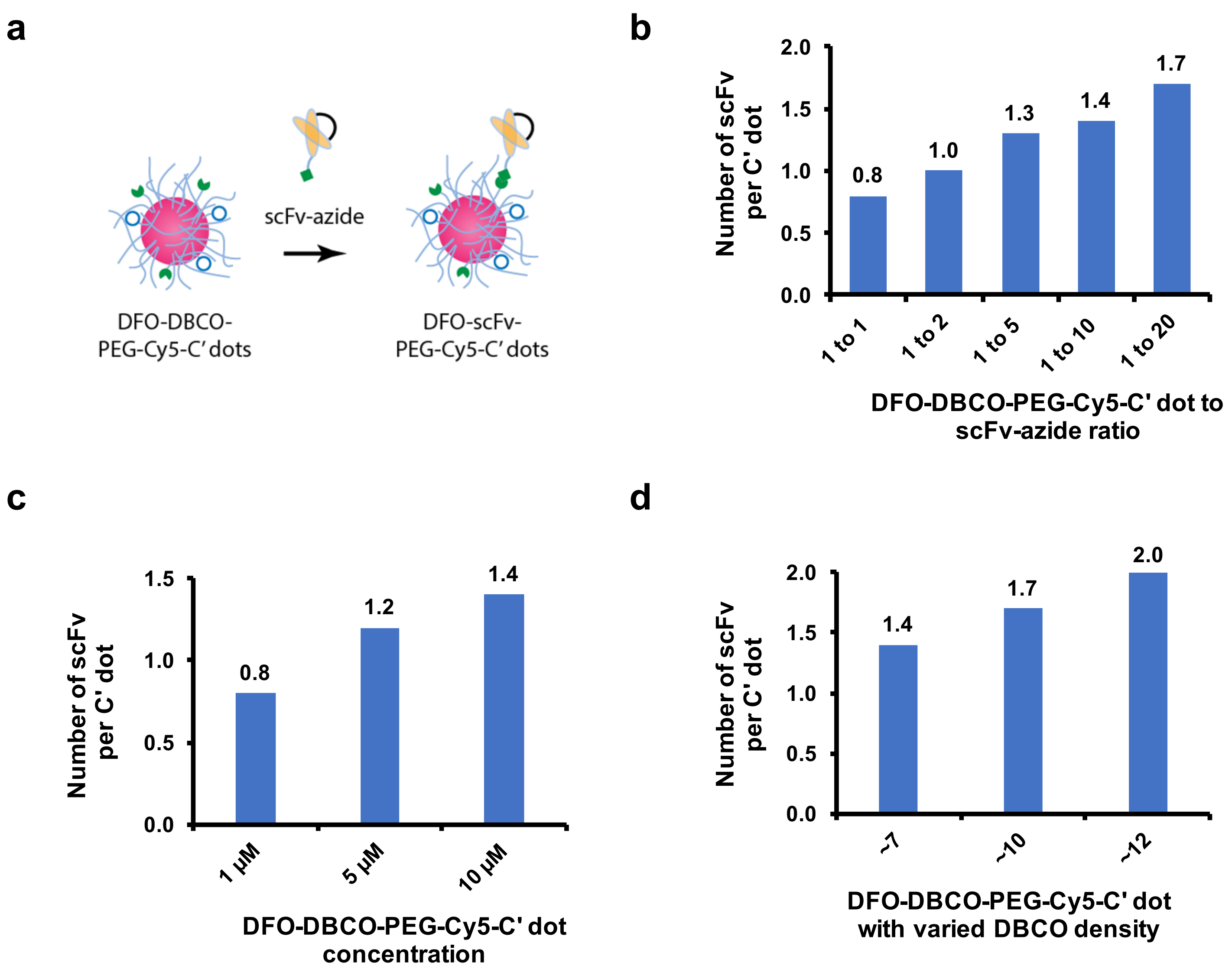

Supplementary Figure 13. Results of different DFO-scFv-PEG-Cy5-C’ dot syntheses with scFv-azide. (**a**) Schematic of functionalization of DFO-DBCO-PEG-Cy5-C’ dots with scFv-azide. (**b**) Precise control of the number of scFv fragments per C’ dot (i.e., from ~0.8 to ~1.7) by maintaining a constant DFO-DBCO-PEG-Cy5-C’ dot concentration (5 µM, 6-7 DBCO per C’ dots) while altering the molar reaction ratio between DFO-DBCO-PEG-Cy5-C’ dots and scFv-azide from 1:1 to 1:20. (**c**) Increasing DFO-DBCO-PEG-Cy5-C’ dot concentration (1 µM - 10 µM; 6-7 DBCO per C’ dot) while maintaining the same molar reaction ratio (i.e., 1:5) between DFO-DBCO-PEG-Cy5-C’ dots and scFv-azide, led to an increased number of scFv fragments per C’ dot (i.e., from ~0.8 to ~1.4). (**d**) The number of scFv fragments per C’ dot could additionally be tuned by reacting DFO-DBCO-PEG-Cy5-C’ dots with varying DBCO surface densities (i.e., 7-12 accessible DBCO molecules per dot) and scFv-azide. For instance, ~2 scFv fragments per C’ dot could be synthesized by reacting DFO-DBCO-PEG-Cy5-C’ dots (5 µM, ~12 DBCO/ C’ dot) with scFv-azide at a molar ratio of 1:5.

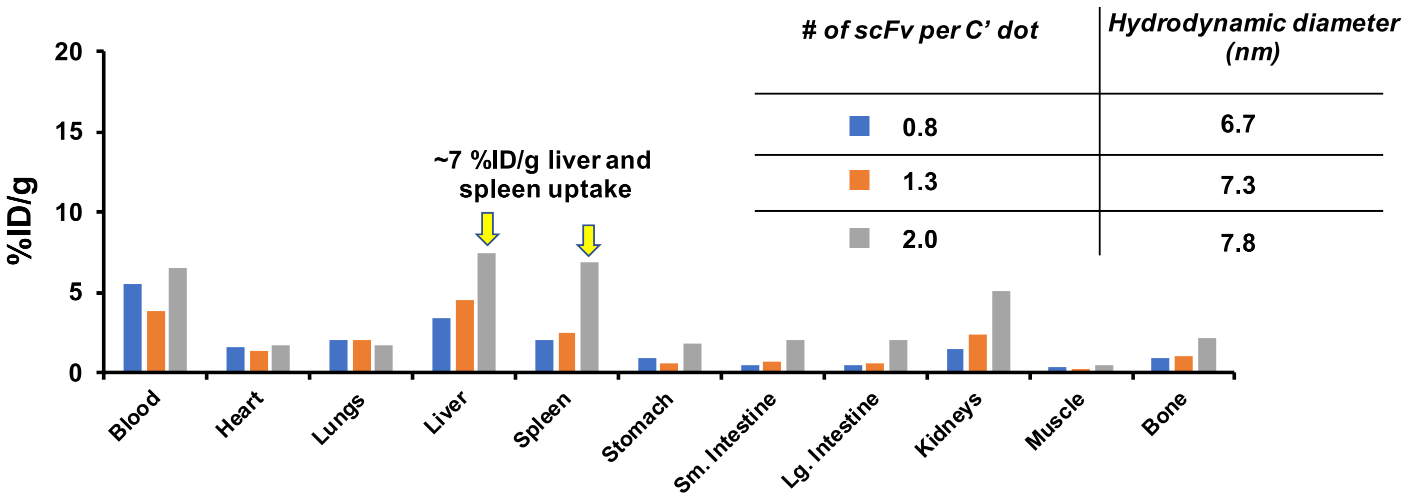

Supplementary Figure 14. Biodistribution study of ^{89}Zr-DFO-scFv-PEG-Cy5-C' dots with varying scFv ligand numbers per dot (i.e., 0.8, 1.3 and 2.0) in healthy mice at a 24 h post injection time point (n=1). While liver and spleen uptake values were found to be less than 5 %ID/g for ^{89}Zr-DFO-scFv-PEG-Cy5-C' dots with scFv ligand numbers per dot equal to or less than 1.3, significantly reduced renal clearance rates, accompanied by increased liver and spleen uptake values, were observed for nanoconjugates bearing 2 scFv fragments per particle due to an increased HD of ~8 nm. All ^{89}Zr-DFO-scFv-PEG-Cy5-C' dots were synthesized using DFO-DBCO-PEG-Cy5-C'dots bearing 6-7 accessible DBCO per C' dot. Inset shows the corresponding hydrodynamic diameters of the tested samples.

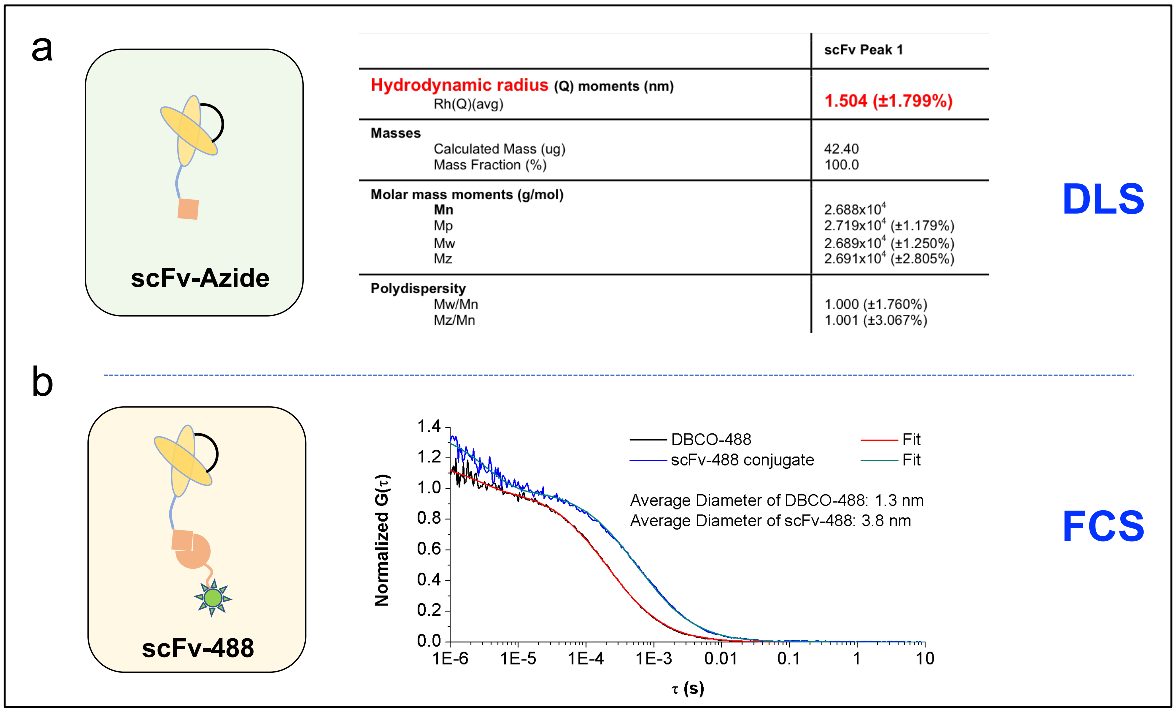

Supplementary Figure 15. (**a**) Left: schematic of a free scFv-azide fragment. Right: DLS measurements of free scFv-azide fragments. SEC-MALS analysis shows a monodisperse peak with a derived MW of 27 kDa, and a hydrodynamic radius of 1.5 nm (or hydrodynamic diameter of 3.0 nm). (**b**) Left: schematic of green-dye- (i.e., DBCO-488) conjugated scFv-azide fragments (i.e., scFv-488). Right: FCS measurements of free DBCO-488 and scFv-488. The hydrodynamic diameters of free DBCO-488 and scFv-488 were estimated to be ~1.3 and 3.8 nm, respectively.

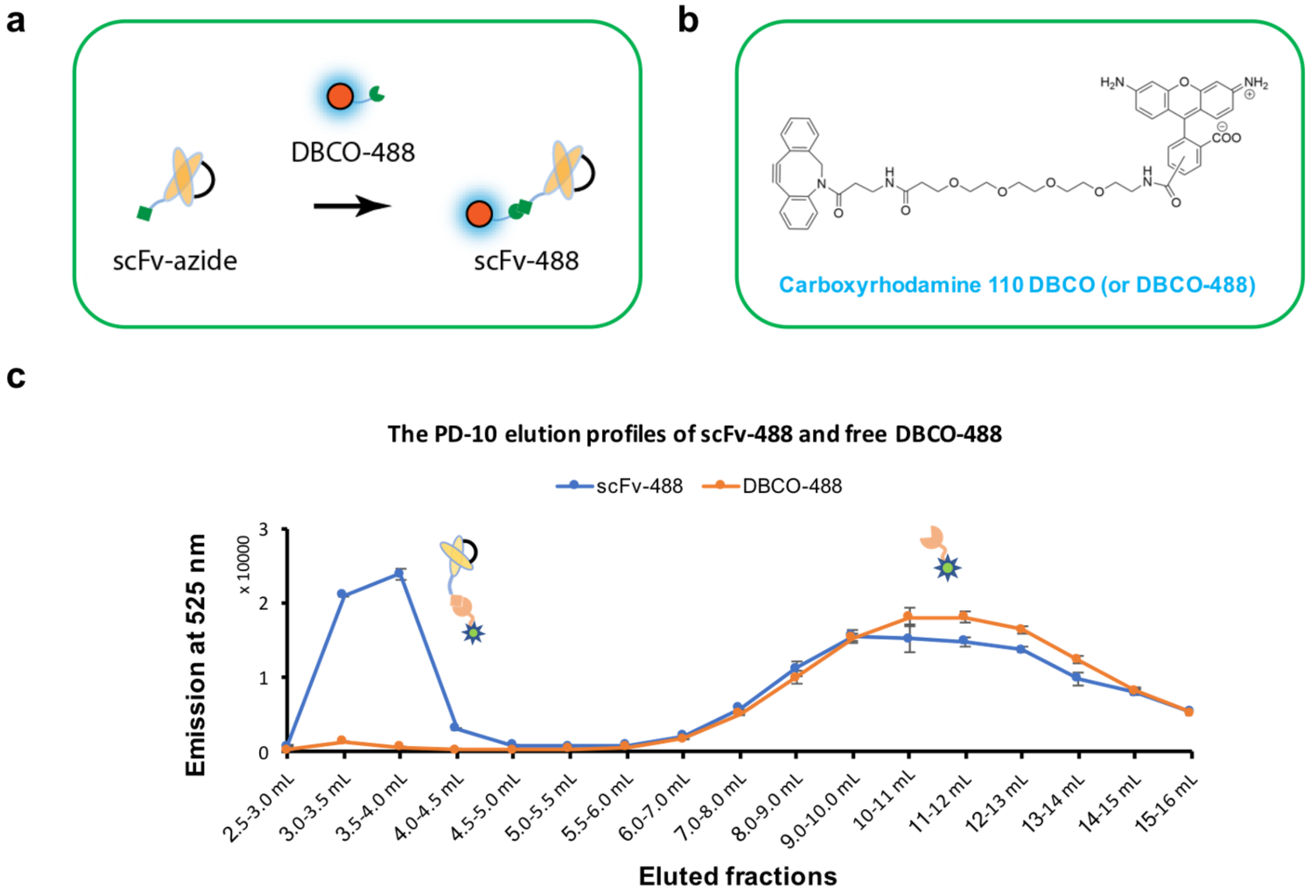

Supplementary Figure 16. (**a**) Schematic illustration showing the reaction of scFv-azide with DBCO-488 (a green dye) to form scFv-488. (**b**) Chemical structure of DBCO-488 (also termed carboxyrhodamine 110 DBCO). (**c**) PD-10 elution profile illustrating the separation between free DBCO-488 and scFv-488. scFv-488 was collected between 2.5 and 4.0 mL fractions, while the free un-reacted DBCO-488 was found to elute after the 6.0 mL fraction.

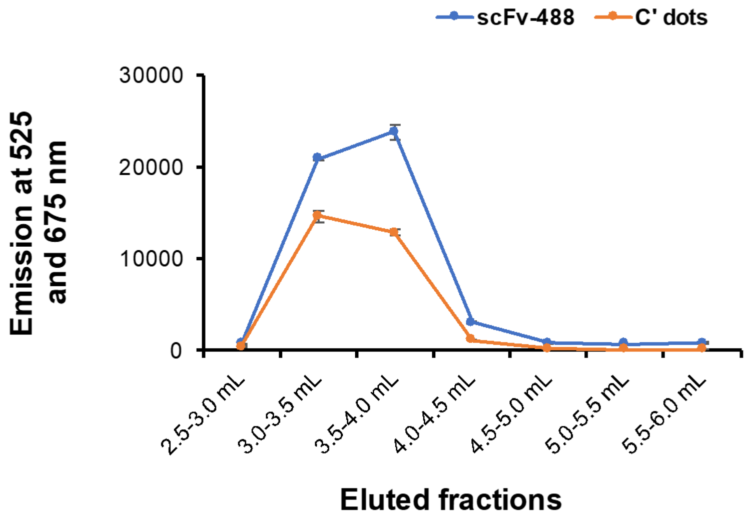

Supplementary Figure 17. PD-10 elution profile of PEG-NH_2-Cy5-C' dot is similar to that of the free scFv-488 fragment, precluding separation of free scFv from DFO-scFv-PEG-Cy5-C' dots using a PD-10 column.

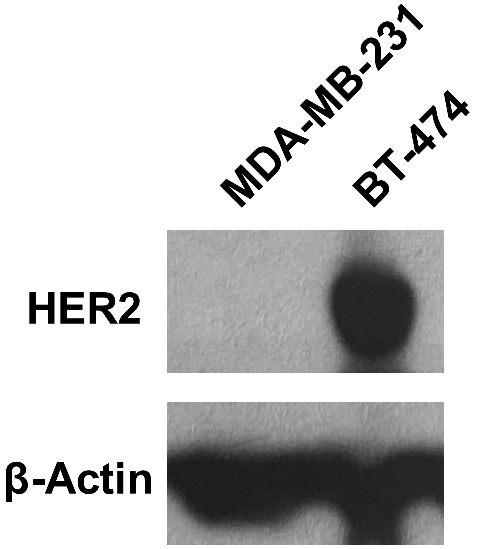

Supplementary Figure 18. Western blot study showing the differences in HER2 expression levels between MDA-MB-231 (HER2 negative) and BT-474 (HER2 positive) cell lines.

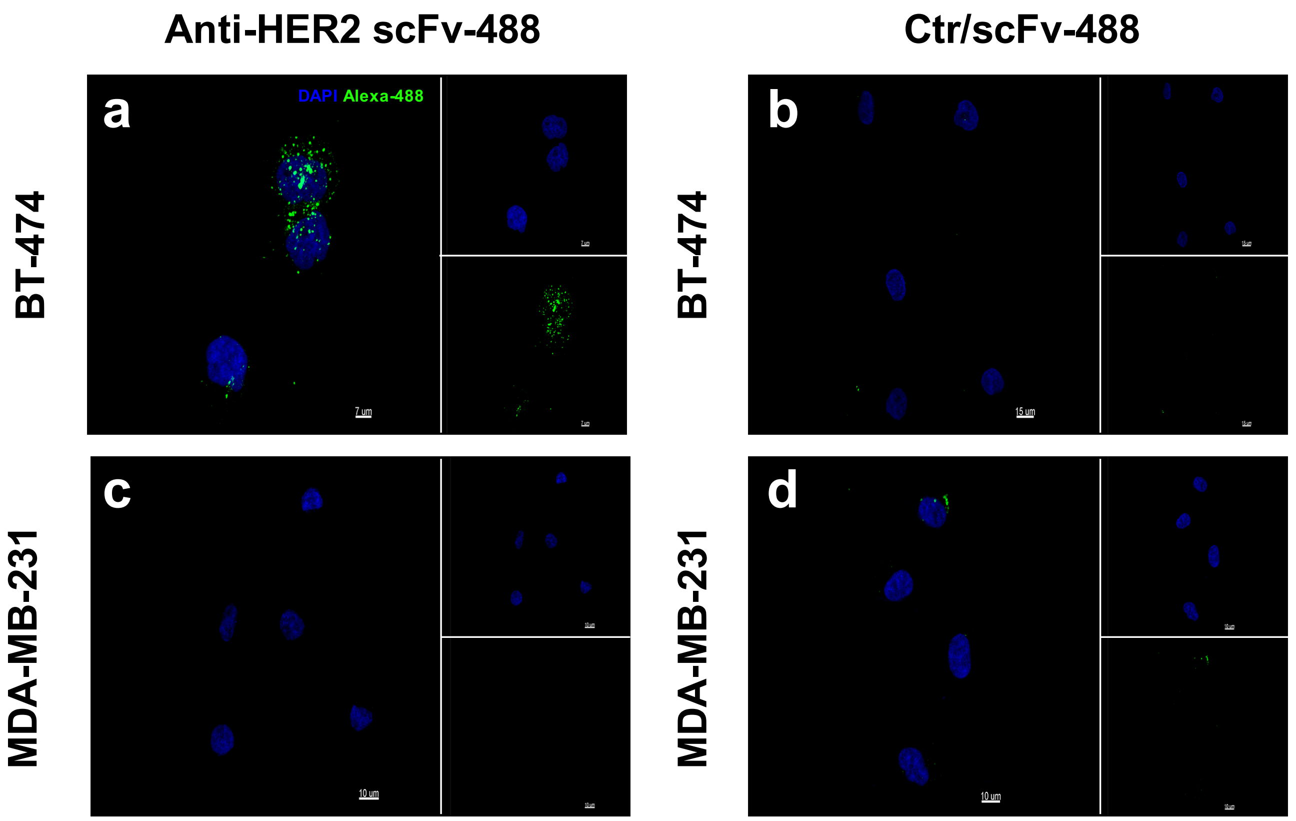

Supplementary Figure 19. Confocal microscopy images of anti-HER2 scFv-488 (100 nM) in (**a**) BT-474 (scale bar: 7 μm) and (**c**) MDA-MB-231 (scale bar: 10 μm), and Ctr/scFv-488 (100 nM) in (**b**) BT-474 (scale bar: 15 μm) and (**d**) MDA-MB-231 (scale bar: 10 μm). Blue and green colors represent DAPI and Alexa-488, respectively. Insets in **a-d** show the corresponding DAPI and Alexa channels. The scale bars in these insets are the same as the corresponding main image.

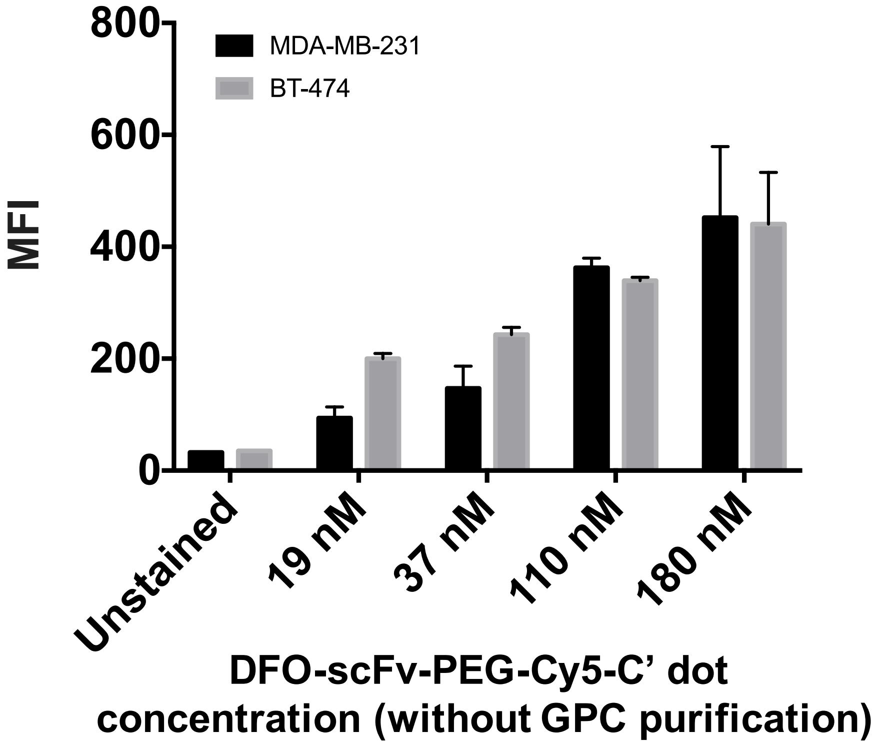

Supplementary Figure 20. *In vitro* anti-HER2 cell targeting of DFO-scFv-PEG-Cy5-C’ dot immunoconjugates without GPC purification. No significant targeting efficiency difference was observed between the HER2 positive and negative cell lines due to the competitive binding of free non-purified scFv to HER2 receptors with DFO-scFv-PEG-Cy5-C’ dots. Without GPC-purification, we estimated that, in the case of incubating 110 nM of DFO-scFv-PEG-Cy5-C’ dots with BT-474, there will be about 300 nM of free scFv-azide present to compete with HER2 receptor binding, resulting in the blocking of DFO-scFv-PEG-Cy5-C’ dot binding to receptors.

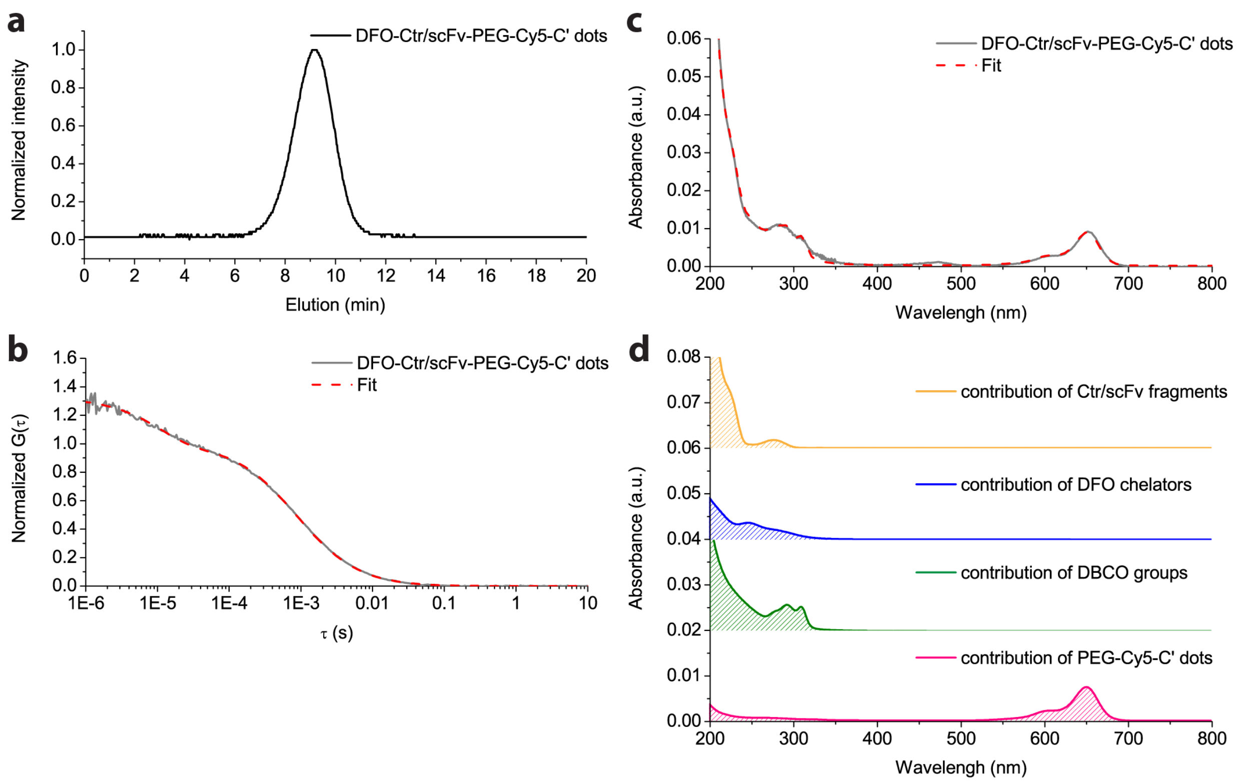

Supplementary Figure 21. Characterization of purified DFO-Ctr/scFv-PEG-Cy5-C' dots. (**a**) GPC elugram of GPC-purified sample. (**b**) FCS correlation curve with fit. (**c**) UV-Vis absorbance spectrum with fit. (**d**) Deconvolution of UV-Vis spectrum into individual contributions from PEG-Cy5-C' dot (red), DBCO (green), DFO (blue), and Ctr/scFv (orange) for quantitative ligand number determination.

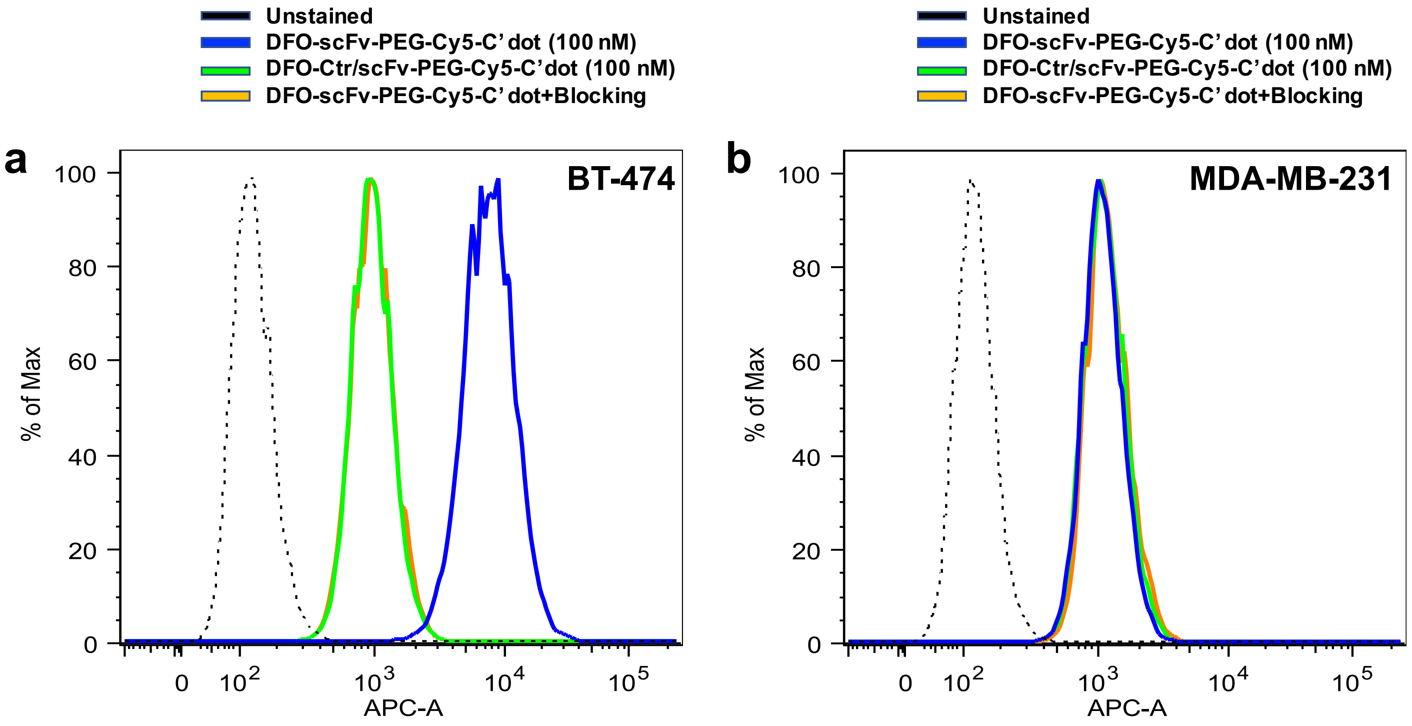

Supplementary Figure 22. *In vitro* flow cytometry studies of scFv-conjugated C' dots in (**a**) BT-474 cell line (HER2 positive) and (**b**) MDA-MB-231 cell line (HER2 negative). *Black bar*: unstained group. *Blue bar*: targeted group, DFO-scFv-PEG-Cy5-C' dot (100 nM). *Green bar*: non-targeted group, DFO-Ctr/scFv-PEG-Cy5-C' dots (100 nM). *Orange bar*: blocking group, DFO-scFv-PEG-Cy5-C' dots (100 nM) + free scFv fragment (20-fold).

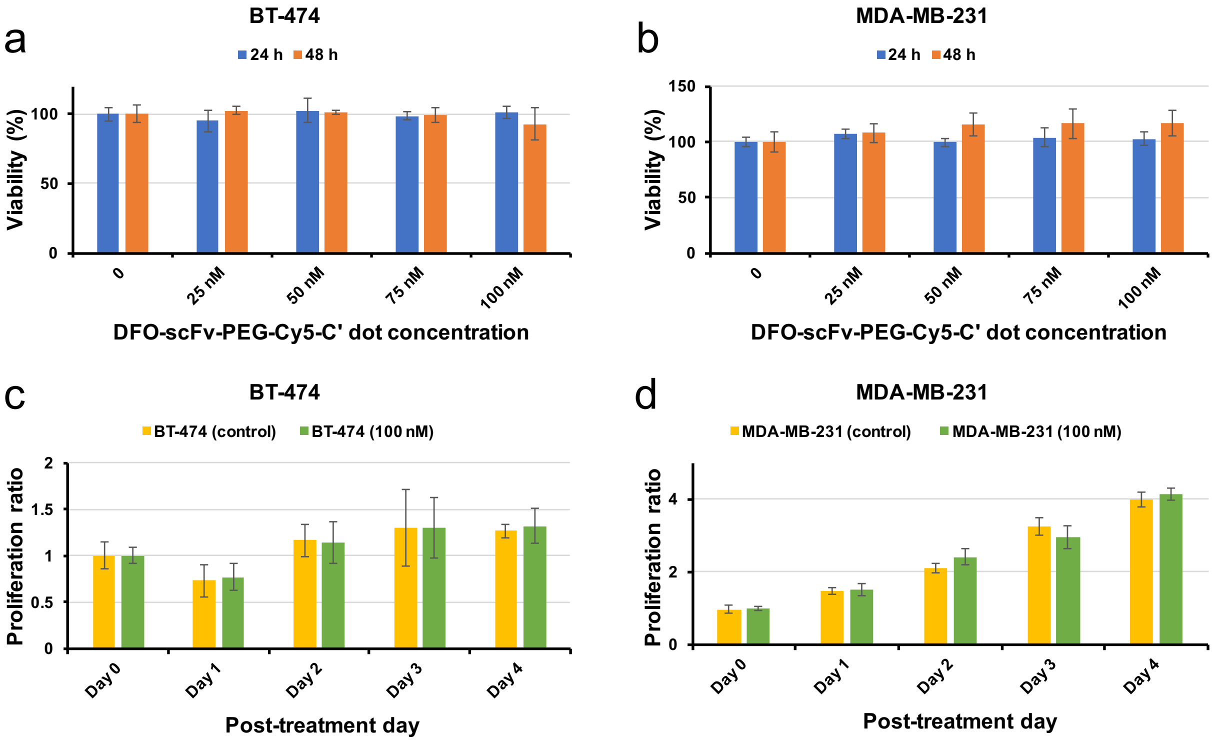

Supplementary Figure 23. ***In vitro* cell viability and proliferation of DFO-scFv-PEG-Cy5-C' dots**. Cell viability of DFO-scFv-PEG-Cy5-C' dots in (**a**) BT-474 and (**b**) MDA-MB-231 cell lines at 24 and 48 h post-incubation times. Particle concentrations range from zero to 100 nM. Cell proliferation of DFO-scFv-PEG-Cy5-C' dots in (**c**) BT-474 and (**d**) MDA-MB-231 cell lines over a 4-day incubation period. Particle concentrations were 100 nM in both cases.

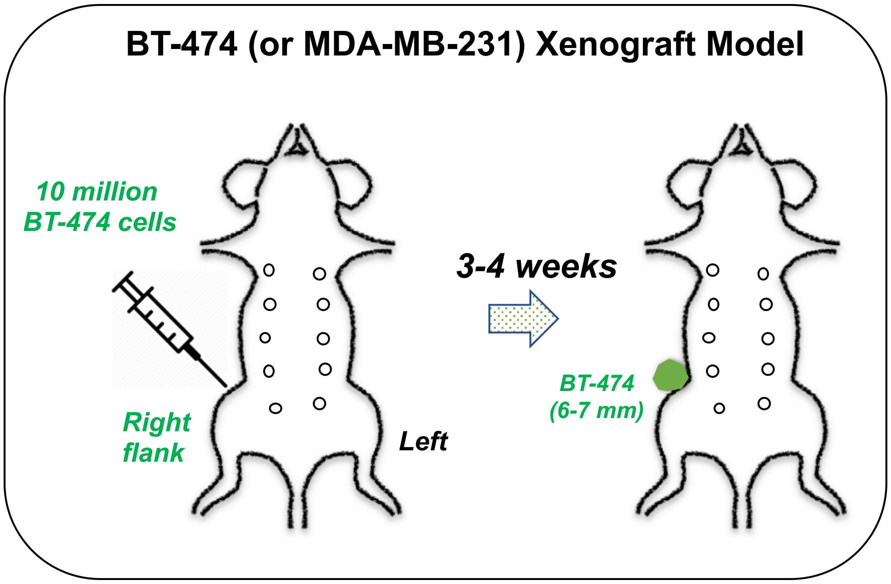

Supplementary Figure 24. Schematic illustration showing the creation of BT-474 or MDA-MB-231 xenografted breast cancer models.

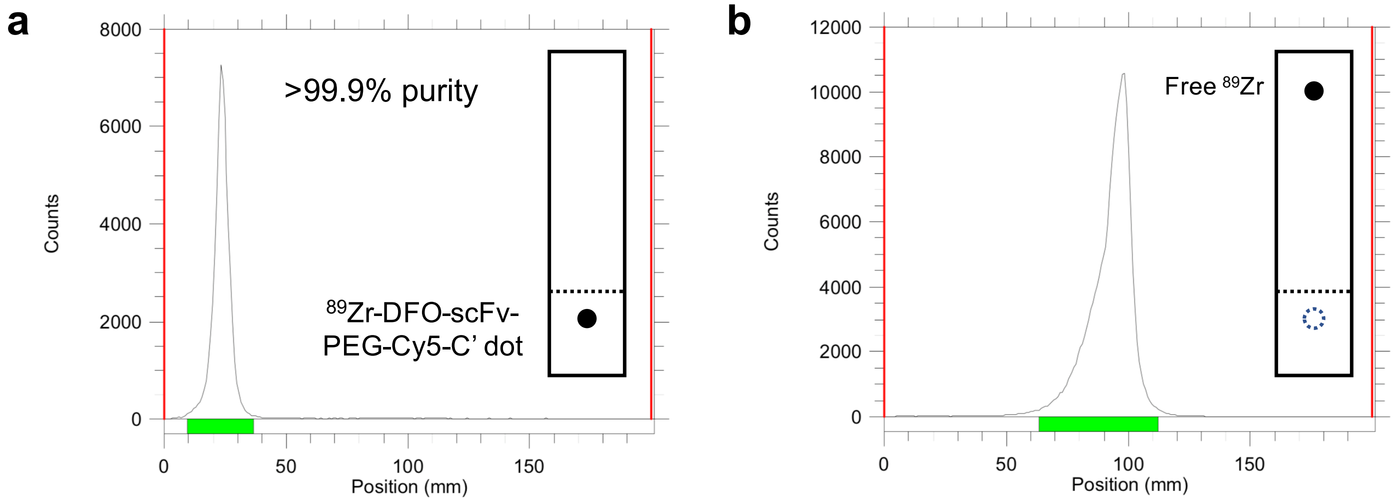

Supplementary Figure 25. Radio-TLC analysis of (**a**) PD-10 purified ^{89}Zr-DFO-scFv-PEG-Cy5-C' dots and (**b**) free ^{89}Zr (mobile phase: EDTA).

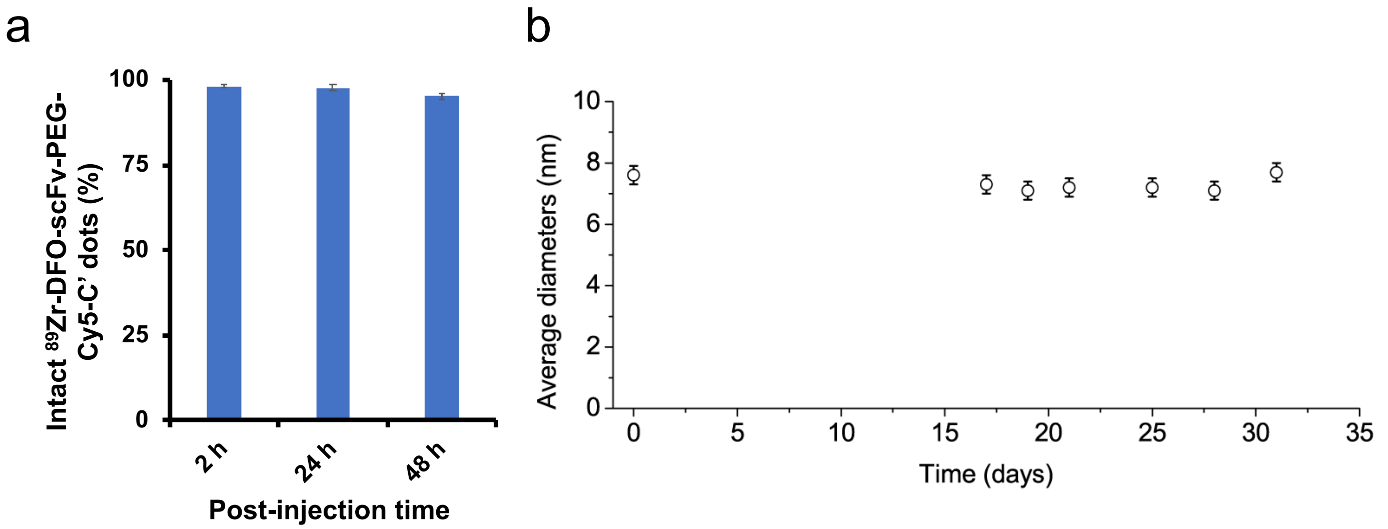

Supplementary Figure 26. (**a**) *In vivo* radiostability of ^{89}Zr-DFO-scFv-PEG-Cy5-C' dots. Non-tumor-bearing nude mice were intravenously injected with ~200 μCi (~7.4 MBq) of ^{89}Zr-DFO-scFv-PEG-Cy5-C' dots. Whole blood specimens were collected at 2, 24 and 48 h post-injection, and the plasma fraction (which contained >98% of the ^{89}Zr-DFO-scFv-PEG-Cy5-C' dots) was separated from the whole blood by centrifugation (8000 rpm for 10 min), and used to test radiopurity with Radio-TLC. (**b**) *Ex vivo* stability of non-radiolabeled DFO-scFv-PEG-Cy5-C' dots in PBS stored at 4 °C and monitored at room temperature by FCS for over a month. Average hydrodynamic diameters did not show any substantial change suggesting good particle stability over this time period.

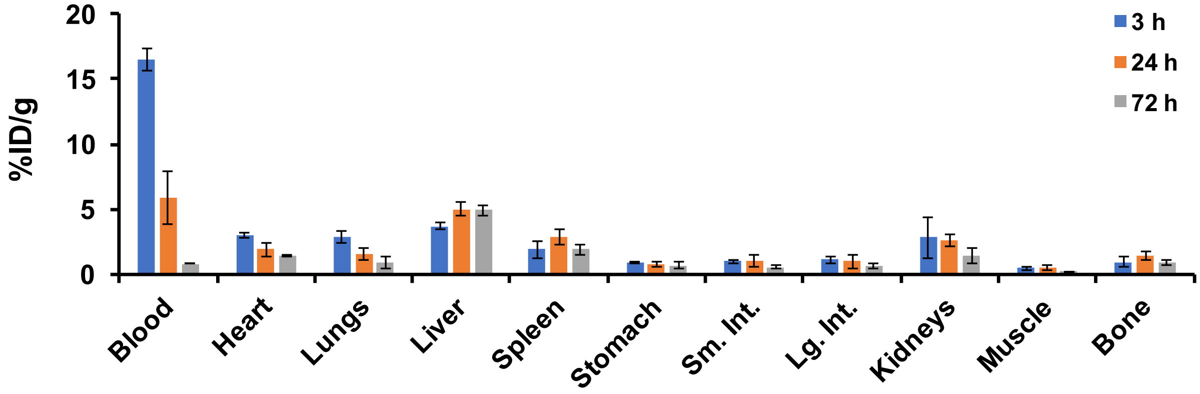

Supplementary Figure 27. Biodistribution study of ^{89}Zr-DFO-scFv-PEG-Cy5-C' dots (1.3 scFv per C' dot) at 3, 24 and 72 hours in healthy nude mice (n=3).

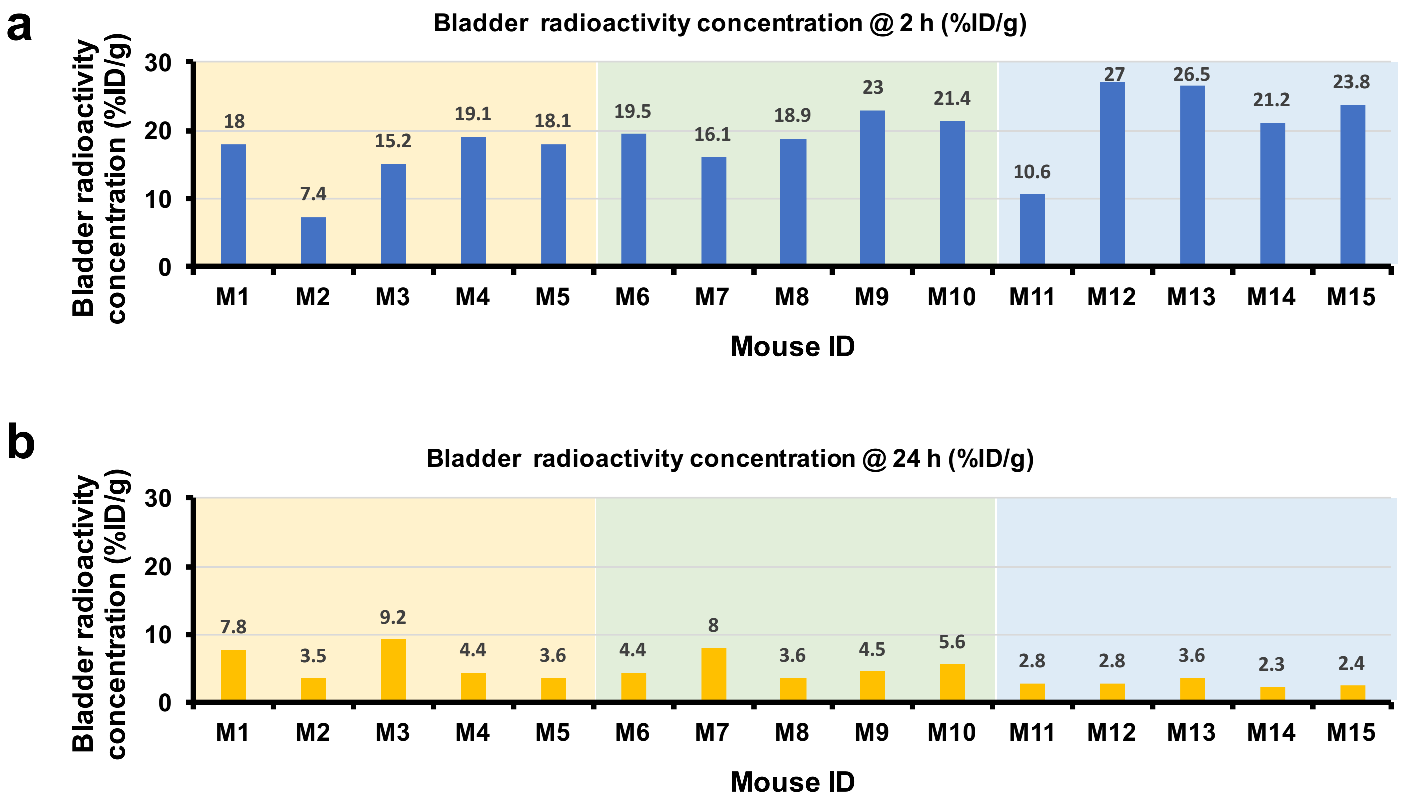

Supplementary Figure 28. Variations in murine bladder radioactivity-concentrations (%ID/g) for all 15 i.v.-injected tumor-bearing mice at 2 and 24 h post-injection. Cohorts were assigned as follows: M1 to M5: BT-474 tumor-bearing mice injected with ^{89}Zr-DFO-scFv-PEG-Cy5-C' dots (*targeted*); M6 to M10: BT-474 tumor-bearing mice injected with ^{89}Zr-DFO-Ctr/scFv-PEG-Cy5-C' dots (*non-targeted*); M10 to M15: MDA-MB-231 tumor-bearing mice injected with ^{89}Zr-DFO-scFv-PEG-Cy5-C' dots (*targeted*).

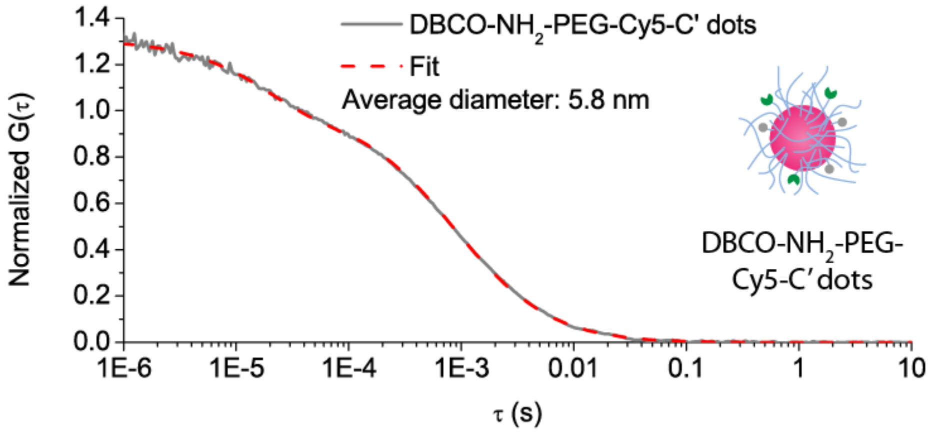

Supplementary Figure 29. FCS correlation curve with a resulting fit to the data indicating 5.8 nm sized DBCO-NH_2-PEG-Cy5-C' dots. Inset shows a schematic of a DBCO-NH_2-PEG-Cy5-C' dot.

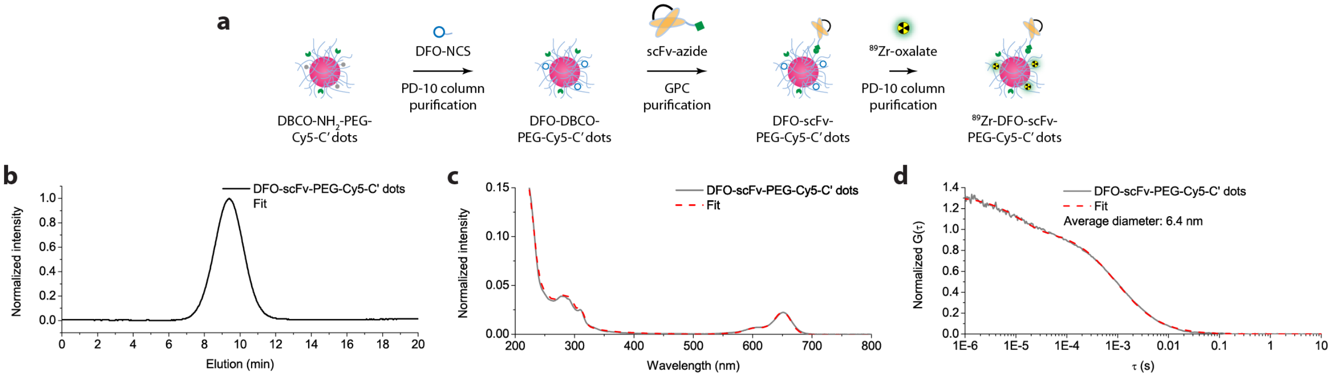

Supplementary Figure 30. **Synthesis and characterization of GPC-purified 6.4 nm sized DFO-scFv-PEG-Cy5-C' dots.** (**a**) Schematic illustration showing synthesis of 6.4 nm DFO-scFv-PEG-Cy5-C' dots starting from the base particle, DBCO-PEG-Cy5-C' dots. First, 5.8 nm DBCO-PEG-NH_2-Cy5-C' dots were synthesized using a modified post-PEGylation surface modification by insertion (PPSMI) technology (**Fig. S30**).[2] Particles were then functionalized with DFO chelator by reacting with DFO-NCS (pH 8-9) to form DFO-DBCO-PEG-Cy5-C' dots. Subsequently, DFO-DBCO-PEG-Cy5-C' dots were mixed with pre-synthesized anti-HER2 scFv-azide in PBS, and reacted overnight, to form DFO-scFv-PEG-Cy5-C' dots. Unreacted scFv fragment was removed via GPC purification. For ^{89}Zr radiolabeling, DFO-scFv-PEG-Cy5-C' dots were mixed with ^{89}Zr-oxalate at pH 7-8 (37 ºC) to form ^{89}Zr-DFO-scFv-PEG-Cy5-C' dots. Free ^{89}Zr was removed using a PD-10 column. The final ^{89}Zr-DFO-scFv-PEG-Cy5-C' dot product was i.v. injected into BT-474 xenografted or orthotopic breast tumor-bearing mice to assess tumor-targeted uptake and PK by serial PET imaging. (**b**) GPC elugram after GPC purification, (**c**) UV-Vis absorbance spectrum with fit of the DFO-scFv-PEG-Cy5-C' dots. (**d**) FCS correlation curve with fit of 6.4 nm sized DFO-scFv-PEG-Cy5-C' dots. Number of scFv per C' dot was estimated to be 1.5, similar to that of 7.3 nm DFO-scFv-PEG-Cy5-C' dots with 1.4 scFv per C' dot.

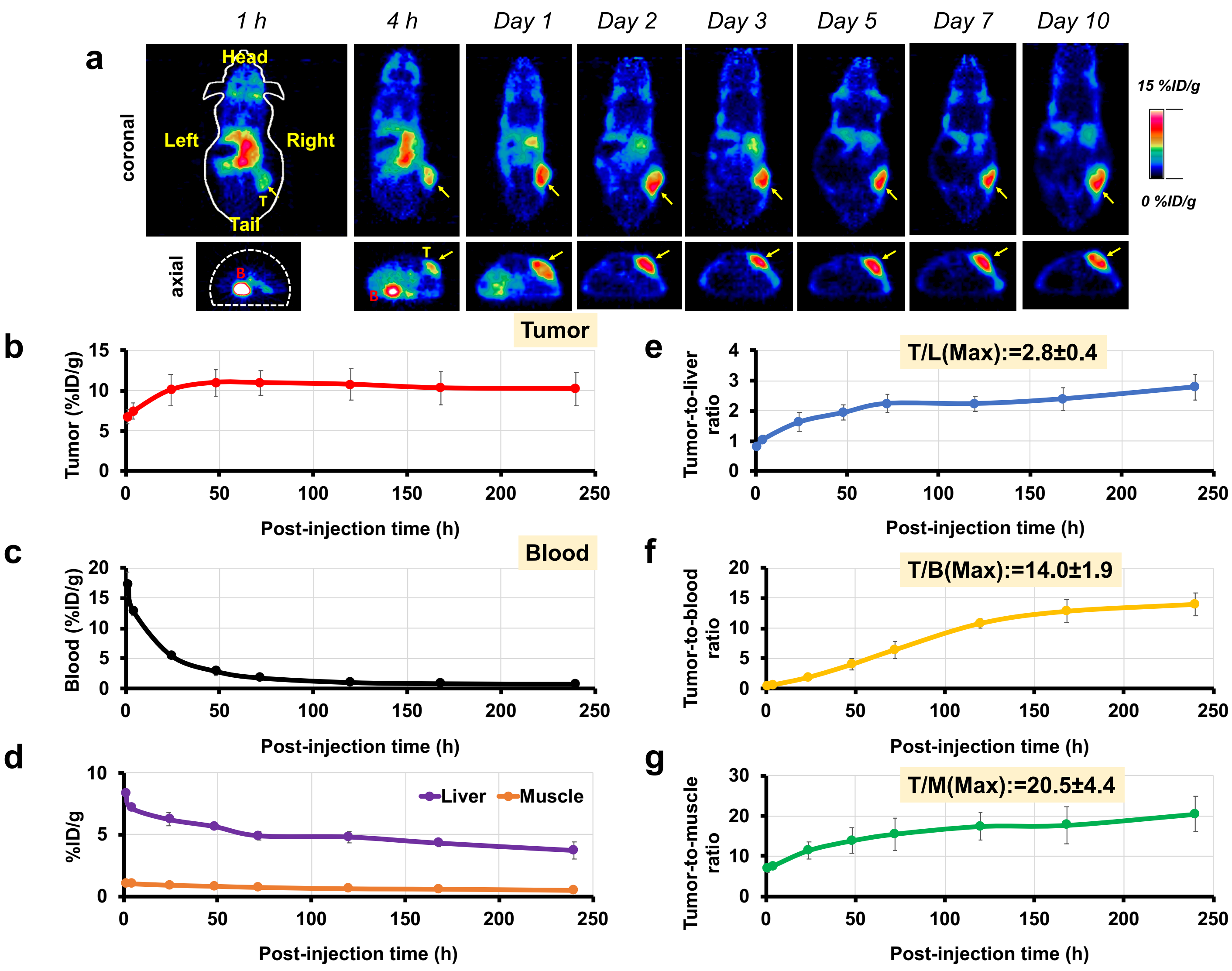

Supplementary Figure 31. **In vivo HER2-targeting in BT-474 breast cancer xenograft model over a 10-day period.** (**a**) Serial coronal and axial tomographic PET images of mice i.v. injected with ^{89}Zr-DFO-scFv-PEG-Cy5-C' dots (HD: 6.4 nm) at 1, 4, 24, 48, 72, 120, 168, and 240 h post-injection (n=3). (**b-d**) Time-activity curves showing major organ uptake of ^{89}Zr-DFO-scFv-PEG-Cy5-C' dots (HD: 6.4 nm) over 10-days. (**e**) Tumor-to-liver, (**f**) tumor-to-blood and (**g**) tumor-to-muscle ratios over a 10-day period.

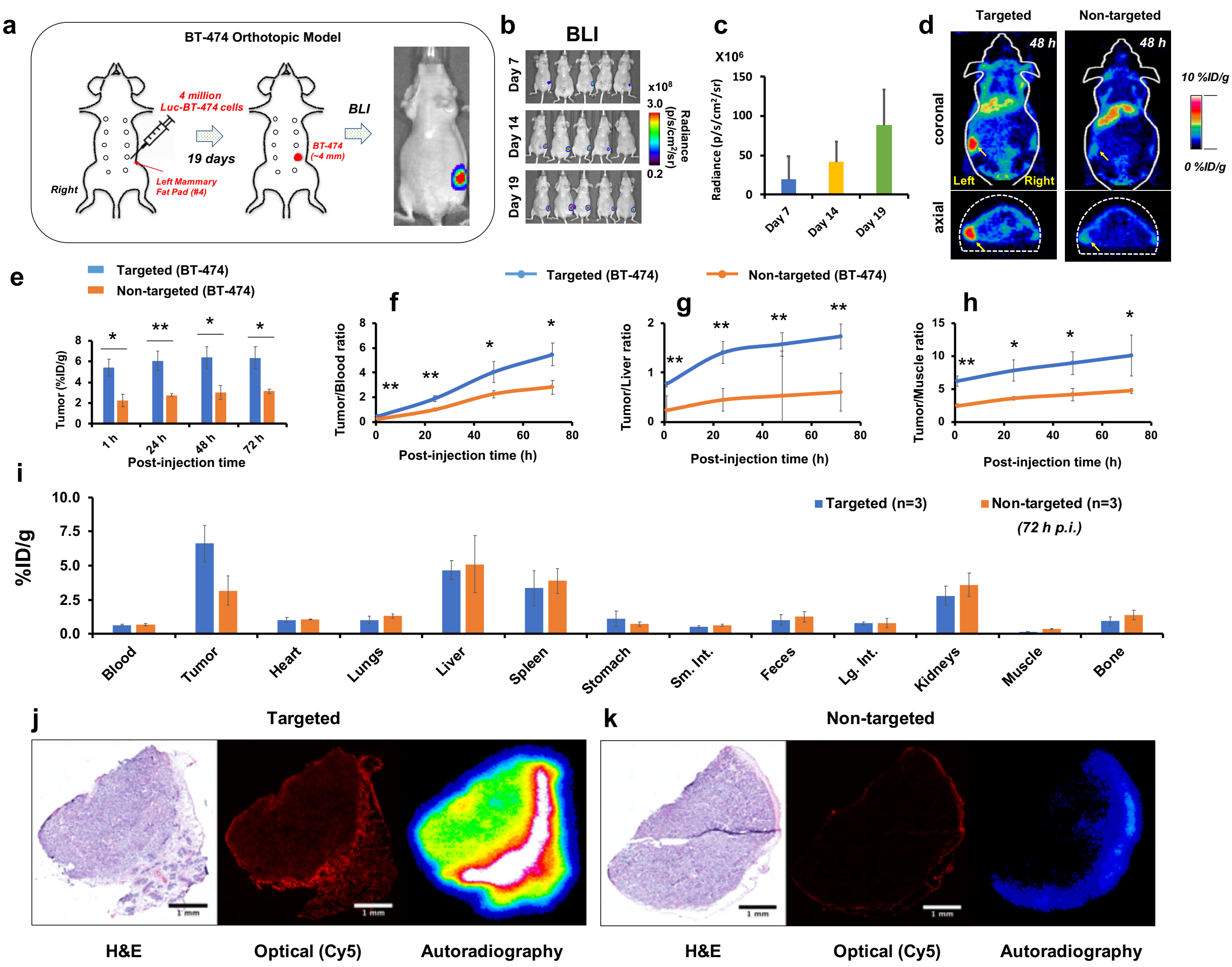

Supplementary Figure 32. In vivo HER2-targeting, *ex vivo* biodistribution and histological studies of ^{89}Zr-DFO-scFv-PEG-Cy5-C' dots (HD: 6.4 nm) in an orthotopic BT-474 breast cancer model. (**a**) Schematic showing generation of the orthotopic BT-474 breast cancer model following transfection and implantation of about 4 million *Luc*+ BT-474 cells into the left mammary fat pad (#4). Tumor growth was monitored by bioluminescence imaging (BLI) using the IVIS Spectrum small animal imaging system, shown in (**b**). (**c**) Quantitative BLI analysis of mice on days 7, 14 and 19 post-implantation. (**d**) Representative coronal and axial tomographic PET images of mice from targeted and non-targeted groups 48h post-injection. Targeted group (n=3 mice): ^{89}Zr-DFO-scFv-PEG-Cy5-C' dots (HD: 6.4 nm). Non-targeted group (n=3 mice): ^{89}Zr-DFO-Ctr/scFv-PEG-Cy5-C' dots (HD: 6.4 nm). Tumors marked with yellow arrows. Comparisons of (**e**) tumor uptake, (**f**) tumor-to-blood ratios, (**g**) tumor-to-liver ratios, (**h**) tumor-to-muscle ratios between the targeted and non-targeted groups. (**i**) Comparison of biodistribution profiles between targeted and non-targeted groups at 72 h post-injection (n=3). (**j-k**) Ex vivo histological study. From left to right: H&E staining, Cy5 widefield fluorescence microscopy, and autoradiograpy of tumor tissue specimens harvested at 72 hours p.i. from (**j**) targeted and (**k**) non-targeted groups. Scale bar: 1 mm. (*P<0.05, **P<0.005)

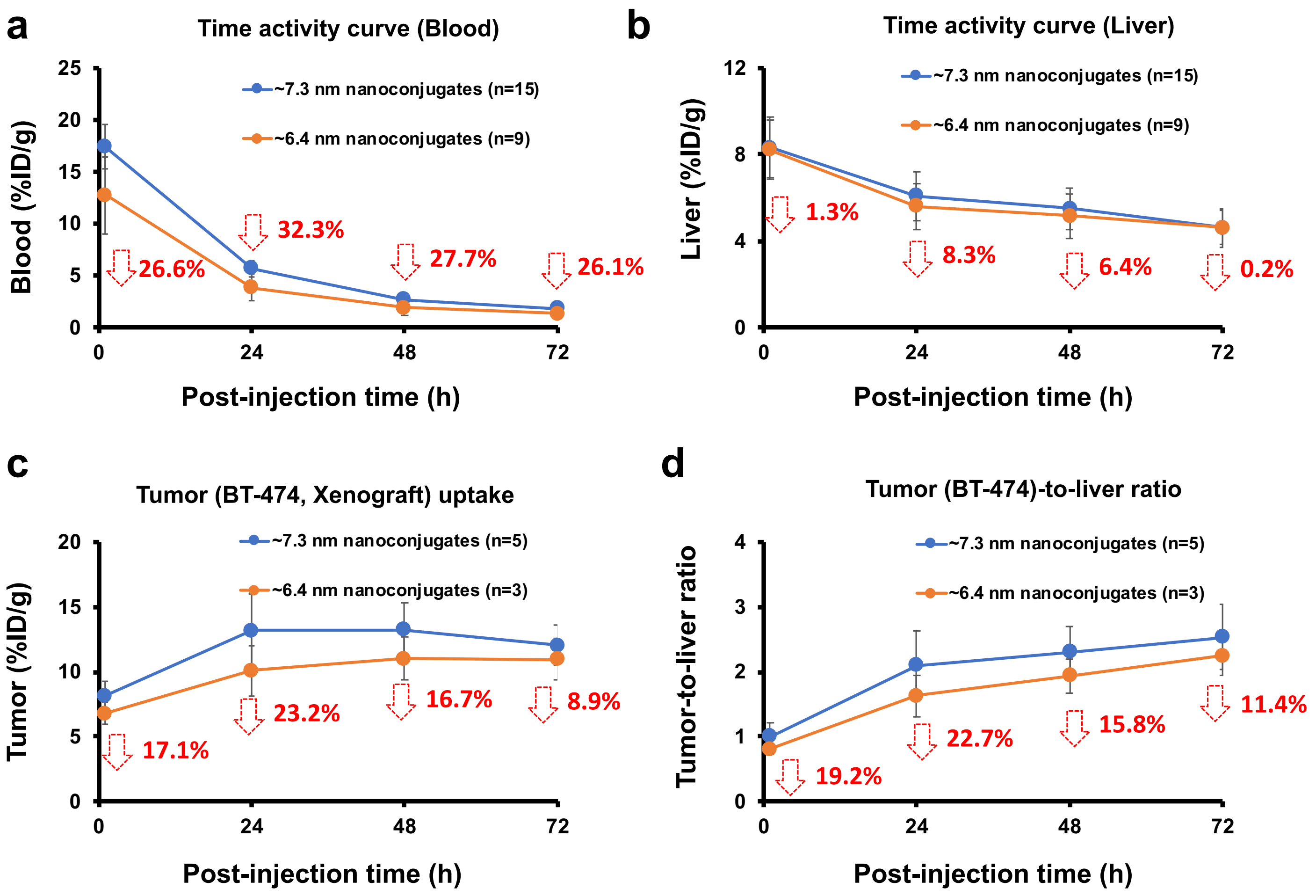

Supplementary Figure 33. Comparison of (**a**) blood, (**b**) liver, and (**c**) tumor uptake as well as (**d**) tumor-to-liver ratios between the 6.4 and 7.3 nm diameter ^{89}Zr-DFO-scFv-PEG-Cy5-C' dots.

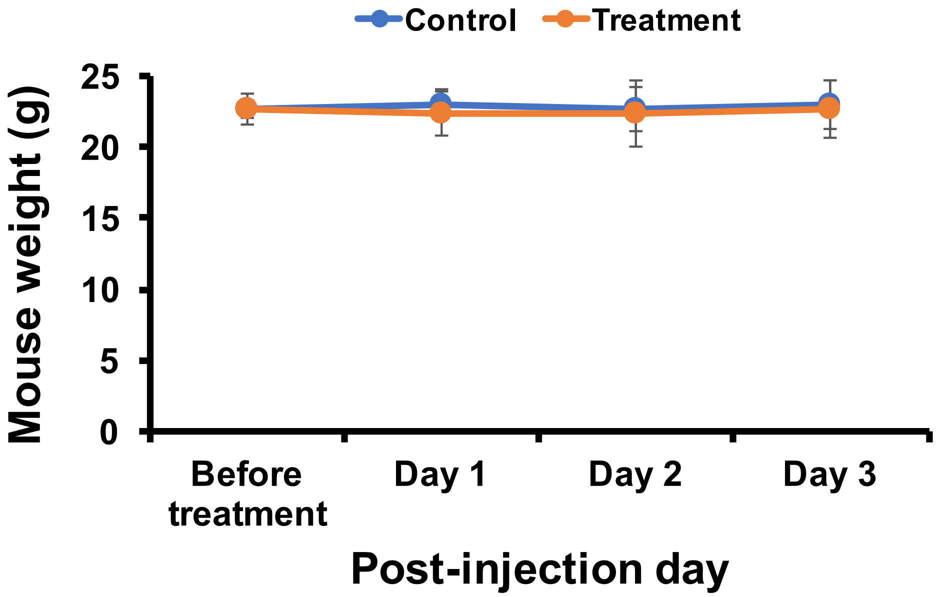

Supplementary Figure 34. Weight change chart of healthy nude female mice injected with saline vehicle (**Control**, n=3) or DFO-scFv-PEG-Cy5-C' dots (**Treatment**, 2 nmols per mouse, n=3). Note: dosing of 2 nmols per mouse was more than **5-fold** that injected during the PET imaging studies.

Section 4. Supplementary Tables

Supplementary Table 1. Calculated equilibrium dissociation constant, $K_D = k_d/k_a$, and on (k_a) and off (k_d) affinity constants for wild-type, HC44, N-term and PEGylated variants. Introduction of nnAA at HC44 and N-term sites alone did not alter affinity of the scFv fragment for the HER2 extracellular domain, while conjugation of a 20 kDa PEG chain reduced overall K_D of molecules, likely via steric hindrance of attached PEG chains.

Sample	KD (M)	KD Error	Ka (1/Ms)	ka Error	kd (1/s)	kd Error
WT	5.18E-09	3.63E-11	3.01E+04	1.70E+02	1.56E-04	6.49E-07
HC44	6.49E-09	4.59E-11	3.34E+04	2.00E+02	2.17E-04	8.10E-07
HC44-PEG	1.30E-08	1.23E-10	4.37E+04	3.70E+02	5.65E-04	2.39E-06
N-term	2.64E-09	1.26E-11	6.99E+04	2.23E+02	1.84E-04	6.60E-07
N-term-PEG	7.29E-09	3.67E-11	4.87E+04	2.03E+02	3.55E-04	1.00E-06

Supplementary Table 2. Characterization summary of aminated C' dots and C' dots functionalized with DFO and antibody fragments.

	Hydrodynamic diameter	By UV-Vis deconvolution				By accessiblity	
		# of Cy5 per C' dot	*# of DBCO per C' dot*	*# of DFO per C' dot*	*# of fragments per C' dot*	*# of DBCO per C' dot*	*# of fragments per C' dot*
**PEG-NH_2-Cy5-C' dots**	6.6	2.2	-	-	-	-	-
**DFO-scFv-PEG-Cy5-C' dots**	7.3	1.9	22	6.6	1.4	7	1.3
**DFO-Ctr/scFv-PEG-Cy5-C' dots**	7.2	2.0	22	7.1	1.6	7	1.3

Supplementary Table 3. Radiation dosimetry of ^{89}Zr-DFO-scFv-PEG-Cy5-C' dots in a 70-kg standard man estimated by the OLINDA dosimetry program.

Tissue	Absorbed Dose (mSv/MBq)
**Adrenals**	0.2622
**Brain**	0.2238
**Breasts**	0.1862
**Gallbladder Wall**	0.3081
**Lower Large Intestine Wall**	0.273
**Small Intestine**	0.303
**Stomach Wall**	0.244
**Upper Large Intestine Wall**	0.278
**Heart Wall**	0.2543
**Kidneys**	0.254
**Liver**	0.4108
**Lungs**	0.1830
**Muscle**	0.1743
**Ovaries**	0.2646
**Pancreas**	0.281
**Red Marrow**	0.2086
**Bone**	0.3297
**Skin**	0.1476
**Spleen**	0.226
**Testes**	0.2105
**Thymus**	0.2165
**Thyroid**	0.2149
**Urinary Bladder Wall**	0.314
**Uterus**	0.281
**Total Body**	0.2097
**Effective Dose**	0.235

Supplementary Table 4. Summary of representative nanoparticle tracers and their respective tumor/liver uptake values and tumor-to-liver (T/L) ratios in small animal models as a function of hydrodynamic diameter, morphology, surface chemistry, and tumor type.[12, 15, 17, 18, 21-37] ^{89}Zr-DFO-scFv-PEG-Cy5-C' dot probe has a T/L ratio >2 as a consequence of successfully controlling surface ligand densities and HD size, thereby ensuring both active targeting and renal clearance.

Nanoparticles	Hydrodynamic diameter (nm)	Targeting ligands	Molecular weight (kDa)	# targeting ligand	Tumor model	Tumor uptake (ave. & max.) (%ID/g)	Liver uptake (%ID/g)	Tumor-to-liver ratio	Renal clearance	Total clearance rate	Quantification method	Reference
[^{198}Au]Au-GSH (a)	~2	---	---	---	---	---	~5	---	Yes	~50 %ID after 2 days	Nuclear imaging	[18]
^{64}Cu-NOTA-Au-GSH (b)	~2	---	---	---	---	---	~0.5	---	Yes	~75 %ID after 1 day	Nuclear imaging	[21]
^{99m}Tc-QDs-GPI (c)	~5	GPI	0.3	5 to 10	---	---	~10	---	Yes	~90 %ID after 1 day	Nuclear imaging	[17]
^{99m}Tc-QDs-cRGD (d)	~5	cRGD peptides	0.6	5 to 10	---	---	~10	---	Yes	~90 %ID after 1 day	Nuclear imaging	[17]
[^{64}Cu]CuS-PVP (e)	5.6	---	---	---	4T1	0.2	4.2	0.05	Yes	~95 %ID after 1 day	Nuclear imaging	[22]
^{124}I-cRGDY-PEG-Cy5-C dot (f)	6.8	cRGDY	0.6	6 to 7	M21	1.8	4.5	0.40	Yes	~97 %ID after 1 week	Nuclear imaging	[15]
^{124}I-cRGDY-PEG-Cy5-C' dot (g)	6 to 7	cRGDY	0.6	18	M21	3.8	6.3	0.60	Yes	>98 %ID after 3 days	Nuclear imaging	[12]
^{89}Zr-DFO-cRGDY-PEG-Cy5-C' dot (h)	6 to 7	cRGDY	0.6	18	M21	12.0	5.3	2.26	Yes	>70 %ID after 3 days	Nuclear imaging	[23]
^{89}Zr-DFO-αMSH-PEG-Cy5-C' dot (i)	6 to 7	αMSH	1.7	8	B16F10	5.5	3.2	1.72	Yes	>70 %ID after 3 days	Nuclear imaging	[24]
**^{89}Zr-DFO-scFv-PEG-Cy5-C' dot (j)**	**7.3**	**scFv fragment**	**25**	**1.4**	**BT-474**	**13.2**	**5.8**	**2.28**	**Yes**	**~70 %ID after 3 days**	**Nuclear imaging**	**This work**
^{111}In-IONP-Trastuzumab (k)	16 (g)	Trastuzumab	150	---	SKBR3	12.9	11.5	1.12	No	---	Nuclear imaging	[25]
^{64}Cu-DOTA-QD-VEGF$_{121}$ (l)	23	VEGF	14	---	U87MG	4.2	45	0.09	No	---	Nuclear imaging	[26]
^{64}Cu-NOTA-Au-IONP-Affibody (m)	24	Affibody	7	---	A431	4.6	22	0.21	No	---	Nuclear imaging	[27]
^{89}Zr-DFO-PPAA-AuNP-cetuximab (n)	31	Cetuximab	150	5 to 10	A431	3.3	13.4	0.25	No	---	Nuclear imaging	[28]
^{64}Cu-DOTA-IONP-RGD (o)	45	cRGD peptides	0.6	35	U87MG	10.1	22.6	0.45	No	---	Nuclear imaging	[29]
[^{198}Au]Au nanosphere (p)	70	---	---	---	EMT6	23.2	34.9	0.66	No	---	Nuclear imaging	[30]
^{111}In-vinorelbine-liposomes (q)	102	---	---	---	C26	25.3	46.1	0.55	No	---	Nuclear imaging	[31]
^{64}Cu-NOTA-MSN-VEGF$_{121}$ (r)	129	VEGF$_{121}$	14	---	U87MG	7.8	17	0.46	No	---	Nuclear imaging	[32]
^{64}Cu-NOTA-CuS@MSN-TRC105 (s)	155	TRC105	150	---	4T1	6	17.1	0.35	No	---	Nuclear imaging	[33]
^{64}Cu-NOTA-MSN-CW800-TRC105(Fab) (t)	175	TRC105(Fab)	47.5	---	4T1	5.4	20	0.27	No	---	Nuclear imaging	[34]
^{64}Cu-NOTA-HMSN-ZW800-TRC105 (u)	194	TRC105	150	---	4T1	9.9	24	0.41	No	---	Nuclear imaging	[35]
^{111}In-Gelatin hydrogel (v)	329	---	---	---	MDA-MB-435	13.9	~20	0.70	No	---	Nuclear imaging	[36]
^{64}Cu-NOTA-SWNT-PEG$_{5400}$-RGD (w)	diameter: 1-5, length: 100-300	cRGD peptides	0.6	~44	U87MG	~12.5	~20	0.63	No	~30 %ID after 1 day	Nuclear imaging	[37]

(a) [^{198}Au]Au-GSH: glutathione (GSH)-coated luminescent gold (Au) nanoparticles intrinsically labeled with ^{198}Au ($t_{1/2}$=2.7 d).[18] Nanoparticles have not been used for in vivo tumor targeting.

(b) ^{64}Cu-NOTA-Au-GSH: GSH-coated Au nanoparticles labeled with ^{64}Cu ($t_{1/2}$=12.6 h) via a NOTA chelator (or 1,4,7-triazacyclononane-1,4,7-trisacetic acid).[21] This nanoparticle has not been used for in vivo tumor targeting.

(c) ^{99m}Tc-QDs-GPI are GPI (a prostate-specific membrane antigen targeted small molecule)-coated quantum dots (QDs) labeled with ^{99m}Tc ($t_{1/2}$=6 h).[17] This nanoparticle has been used for in vivo tumor targeting, however, no quantitative tumor uptake data was reported.

(d) ^{99m}Tc-QDs-cRGD is cRGD (an integrin $\alpha_v\beta_3$ targeted peptide)-coated quantum dots (QDs) labeled with ^{99m}Tc ($t_{1/2}$=6 h).[17] This nanoparticle has been used for in vivo tumor targeting, however, no quantitative tumor uptake data was reported.

(e) [^{64}Cu]CuS-PVP are PVP (polyvinylpyrrolidone)-protected ultrasmall copper sulfide (CuS) nanodots intrinsically labeled with ^{64}Cu ($t_{1/2}$=12.6 h).[22]

[(f)] ^{124}I-cRGDY-PEG-Cy5-C dots are first generation renally-clearable Cornell dots (or C dots) functionalized with cRGDY, Cy5 dye, PEG, and ^{124}I-labeled for melanoma tumor imaging.[15]

[(g)] ^{124}I-cRGDY-PEG-Cy5-C' dot is the new generation of renally-clearable Cornell prime dots (or C' dot) functionalized with cRGDY, Cy5 dye, PEG, and ^{124}I-labeled for melanoma tumor imaging.[12]

[(h)] ^{89}Zr-DFO-cRGDY-PEG-Cy5-C' dots are renally clearable C' dots functionalized with cRGDY, Cy5 dye, PEG, and labeled with ^{89}Zr via the DFO chelating moiety (or deferoxamine) for melanoma tumor imaging.[23]

[(i)] ^{89}Zr-DFO-αMSH-PEG-Cy5-C' dots are renally-clearable C' dots functionalized with αMSH (13-amino acid peptide, alpha melanocyte stimulating hormone), Cy5 dye, PEG, and labeled with ^{89}Zr via a DFO chelator for melanoma tumor imaging.[24]

[(j)] ^{89}Zr-DFO-scFv-PEG-Cy5-C' dots is an anti-HER2 single chain antibody fragment conjugated C' dot labeled with ^{89}Zr ($t_{1/2}$=78.4 h) via a DFO chelator.

[(k)] ^{111}In-IONP-Trastuzumabis is an indium-111 (^{111}In, $t_{1/2}$=2.8 d) labeled multifunctional superparamagnetic iron oxide nanoparticle (IONP).[25] The 16 nm size is based on TEM, not DLS, of the final antibody-conjugated nanoparticle.

[(l)] ^{64}Cu-DOTA-QD-VEGF$_{121}$ is vascular endothelial growth factor 121 (VEGF$_{121}$) conjugated QD labeled with ^{64}Cu ($t_{1/2}$=12.6 h) via a DOTA chelator (or 1,4,7,10-Tetraazacyclododecane-1,4,7,10-tetraacetic acid).[26]

[(m)] ^{64}Cu-NOTA-Au-IONP-Affibody is Affibody-conjugated gold-iron oxide hetero-nanostructures labeled with ^{64}Cu ($t_{1/2}$=12.6 h) via the chelator of NOTA.[27]

[(n)] ^{89}Zr-DFO-PPAA-AuNP-cetuximab is a cetuximab-conjugated Au nanoparticle labeled with ^{89}Zr ($t_{1/2}$=78.4 h) via a DFO chelator (or deferoxamine).[28] The gold nanoparticles were surface modified with PPAA (or plasma-polymerized allylamine).

[(o)] ^{64}Cu-DOTA-INOP-RGD is an RGD peptide-conjugated INOP labeled with ^{64}Cu ($t_{1/2}$=12.6 h) via a DOTA chelator. [29]

[(p)] [^{198}Au]Au nanosphere is an intrinsically ^{198}Au ($t_{1/2}$=2.7 d) labeled Au nanosphere.[30]

[(q)] ^{111}In-vinorelbine-liposomes is an indium-111 (^{111}In, $t_{1/2}$=2.8 d) labeled vinorelbine-encapsulated liposome.[31] Vinorelbine (NVB), sold under the brand name Navelbine, among others, is a chemotherapy agent used to treat many tumor types, including breast cancer and non-small cell lung cancer. It is given by venous injection or administered orally (Source: *https://en.wikipedia.org/wiki/Vinorelbine*).

[(r)] ^{64}Cu-NOTA-MSN-VEGF$_{121}$ is VEGF$_{121}$ conjugated mesoporous silica nanoparticles (MSN) labeled with ^{64}Cu ($t_{1/2}$=12.6 h) via a NOTA (or 1,4,7-triazacyclononane-1,4,7-trisacetic acid) chelator.[32]

[(s)] ^{64}Cu-NOTA-CuS@MSN-TRC105 is TRC105 (a human/murine chimeric IgG1 monoclonal antibody, which binds to human and murine CD105 on tumor neovasculature) conjugated MSN-coated CuS nanoparticles (CuS@MSN) labeled with ^{64}Cu ($t_{1/2}$=12.6 h) via a NOTA chelator.[33]

[(t)] ^{64}Cu-NOTA-MSN-CW800-TRC105(Fab) is TRC105(Fab) antibody fragment conjugated MSN labeled with ^{64}Cu ($t_{1/2}$=12.6 h) via the chelator of NOTA. The nanoparticles were also surface tagged with near-infrared organic dyes, CW800 (*Ex: 774 nm; Em: 789 nm*).[34]

[(u)] ^{64}Cu-NOTA-HMSN-ZW800-TRC105 is TRC105 antibody-conjugated hollow MSN (HMSN) labeled with ^{64}Cu ($t_{1/2}$=12.6 h) via the chelator of NOTA. The nanoparticles were also surface tagged with near-infrared organic dyes, ZW800 (*Ex: 745 nm; Em: 800 nm*).[35]

[(v)] ^{111}In-Gelatin hydrogel is an indium-111 (^{111}In, $t_{1/2}$=2.8 d) labeled gelatin hydrogel nanoparticle.[36]

[(w)] ^{64}Cu-NOTA-SWNT-PEG$_{5400}$-RGD is RGD peptides conjugated single-walled carbon nanotubes (SWNT) labeled with ^{64}Cu ($t_{1/2}$=12.6 h) via a NOTA chelator.[37] The nanoparticles were surface-modified with PEG$_{5400}$ to improve in vivo stability and blood circulation half-life.

Supplementary Table 5. Complete metabolic profiles of female mice injected with either saline vehicle (**Control group**, n=3) versus DFO-scFv-PEG-Cy5-C' dots (**Treatment group**, 2 nmols/mouse, n=3)

	**Sample ID**	**#1 Saline**	**#2 Saline**	**#3 Saline**	**#1 DFO-scFv-PEG-Cy5-C' dots**	**#2 DFO-scFv-PEG-Cy5-C' dots**	**#3 DFO-scFv-PEG-Cy5-C' dots**
	**Sex**	**F**	**F**	**F**	**F**	**F**	**F**
**Renal**	**BUN (mg/dL)**	26	29	28	28	26	19
	**CREA (mg/dL)**	0.21	0.17	0.23	0.18	0.18	0.17
	**BUN/CREA ratio**	123.8	170.6	121.7	155.6	144.4	111.8
**Hepatic function**	**ALP (U/L)**	107	173	150	148	156	98
	**ALT (U/L)**	36	37	66	35	58	28
	**AST (U/L)**	80	181	105	227	95	87
	**GGT (U/L)**	0.0	0.0	0.0	0.0	0.0	0.0
	**TBIL (mg/dL)**	0.2	0.2	0.2	0.2	0.2	0.2
	**DBIL (mg/dL)**	0.0	0.0	0.0	0.0	0.0	0.0
	**IBIL (mg/dL)**	0.2	0.2	0.2	0.2	0.2	0.2
	**TP (g/dL)**	5.3	5.4	5.7	5.1	5.2	5.1
	**ALB (g/dL)**	3.2	3.4	3.3	3.1	3.2	3.1
	**GLOB (g/dL)**	2.1	2.0	2.4	2.0	2.0	2.0
	**A/G ratio**	1.5	1.7	1.4	1.6	1.6	1.6
**Metabolic profile**	**P (mg/dL)**	11.0	8.3	9.0	10.2	9.8	8.6
	**Ca (mg/dL)**	9.9	9.6	10.4	9.9	9.6	9.3
	**GLU (mg/dL)**	198	187	238	241	215	246
	**CHOL (mg/dL)**	102	107	135	102	93	103
	**TRIG (mg/dL)**	132	146	249	168	140	82
	**CK (U/L)**	100	723	157	1029	43	252
	**TCO2 (mEq/L)**	28	25	24	28	27	28
	**Na (mEq/L)**	153	154	153	152	152	151
	**K (mEq/L)**	8.2	6.4	8.2	8.5	8.8	7.6
	**Cl (mEq/L)**	111	113	110	107	109	109
	**Na/K ratio**	19	24	19	18	17	20
	**Anion Gap**	22	22	27	26	25	22

BUN *: Blood urea nitrogen;* ***CREA*** *: Creatinine;* ***ALP*** *: Alkaline phosphatas;* ***ALT*** *: Alanine transaminase;* ***AST*** *: Aspartate transaminase;* ***GGT*** *: Gamma-Glutamyl Transferase;* ***TBIL*** *: Total bilirubin;* ***DBIL*** *: Direct bilirubin;* ***IBIL*** *: Indirect bilirubin;* ***TP*** *: Total protein;* ***ALB*** *: Albumin;* ***GLOB*** *: Globulin;* ***A/G*** *: Albumin/Globulin;* ***P*** *: Phosphor;* ***Ca*** *: Calcium;* ***GLU*** *: Glucose;* ***CHOL*** *: Cholesterol;* ***TRIG*** *: Triglycerides;* ***CK*** *: Creatine Kinase;* ***TCO2*** *: Total amount of carbon dioxid.*

Supplementary Table 6. Complete blood counts of mice injected with saline vehicle (**Control group**, n=3) versus DFO-scFv-PEG-Cy5-C' dots (**Treatment group**, 2 nmols/mouse, n=3).

Test Name	Range	#1 Saline	#2 Saline	#3 Saline	#1 DFO-scFv-PEG-Cy5-C'dots	#2 DFO-scFv-PEG-Cy5-C'dots	#3 DFO-scFv-PEG-Cy5-C'dots
**RBC (M/uL)**	6.82-10.53	8.76	8.63	9.43	9.49	9.54	8.36
**HGB (g/dL)**	10.9-15.9	15.2	14.4	15.6	16.3	15.8	14.9
**HCT (%)**	34.9-59.6	47.8	46.0	49.6	51.8	50.1	47.6
**MCV (fL)**	44.5-71.9	54.6	53.3	52.6	54.6	52.5	56.9
**MCH (pg)**	11.5-18.0	17.4	16.7	16.5	17.2	16.6	17.8
**MCHC (g/dL)**	23.2-34.5	31.8	31.3	31.5	31.5	31.5	31.3
**RDW-SD (fL)**	--	26.9	27.2	26.4	27.3	28.1	28.3
**RDW-CV (%)**	15.0-22.7	19.5	19.7	20.7	20.4	21.3	19.0
**RET# (K/uL)**	294-444	383.7	413.4	428.1	400.5	365.4	520.8
**RET (%)**	2.56-4.56	4.38	4.79	4.54	4.22	3.83	6.23
**PLT (K/uL)**	376-2206	887.0	425.0	760.0	313.0	744.0	644.0
**PDW (fL)**	--	7.0	6.6	7.1	7.1	6.9	6.6
**MPV (fL)**	4.2-5.9	6.2	6.4	6.3	6.6	6.3	6.2
**Morphology**		3+ polychromasia.	A manual differential was not performed due to low WBC count (<2.00 K/uL).	A manual differential was not performed due to low WBC count (<2.00 K/uL).	3+ polychromasia.	2+ polychromasia.	4+ polychromasia.

***RBC**: Red Blood Cell Count; **HGB**: Hemoglobin; **HCT**: Hematocrit; **MCV**: Mean corpuscular volume; **MCH**: Mean Corpuscular Hemoglobin; **MCHC**: Mean corpuscular hemoglobin concentration; **RDW**: Red blood cell distribution width; **RET**: Reticulocyte; **PLT**: Platelets; **PDW**: Platelet Distribution Width; **MPV**: Mean platelet volume*

***Disclaimer**: Reference ranges are based on published data sourced from Charles River and IDEXX laboratories, and are strain-specific for mice whenever possible. Published values do not necessarily correlate with in-house values, due to variability in testing equipment, background strain/substrain, sex, and age differences. Because of this variability, it is recommended that values be compared to control animals whenever possible.*

Supplementary Table 7. Complete automated differential blood counts of mice injected with saline vehicle (**Control group**, n=3) versus DFO-scFv-PEG-Cy5-C' dots (**Treatment group**, 2 nmols/mouse, n=3).

Test Name	Reference	*#1 Saline*	*#2 Saline*	*#3 Saline*	*#1 DFO-scFv-PEG-Cy5-C'dots*	*#2 DFO-scFv-PEG-Cy5-C'dots*	*#3 DFO-scFv-PEG-Cy5-C'dots*
**WBC# (K/uL)**	*1.42-10.25*	*2.75*	*0.76*	*1.31*	*2.04*	*5.77*	*3.46*
**NEUT# (K/uL)**	*0.43-3.58*	*0.80*	*0.26*	*0.40*	*0.45*	*1.21*	*1.02*
**LYMPH# (K/uL)**	*0.49-6.87*	*1.73*	*0.44*	*0.66*	*1.47*	*4.12*	*2.22*
**MONO# (K/uL)**	*0.03-0.65*	*0.11*	*0.05*	*0.16*	*0.09*	*0.34*	*0.13*
**EO# (K/uL)**	*0.01-0.53*	*0.09*	*0.01*	*0.08*	*0.03*	*0.09*	*0.08*
**BASO# (K/uL)**	*0.00-0.21*	*0.02*	*0.00*	*0.01*	*0.00*	*0.01*	*0.01*
**NEUT (%)**	*13.96-71.49*	*29.1*	*34.2*	*30.5*	*22.0*	*20.9*	*29.4*
**LYMPH (%)**	*23.63-79.28*	*62.9*	*57.9*	*50.4*	*72.1*	*71.4*	*64.2*
**MONO (%)**	*0.86-12.15*	*4.0*	*6.6*	*12.2*	*4.4*	*5.9*	*3.8*
**EO (%)**	*0.29-7.18*	*3.3*	*1.3*	*6.1*	*1.5*	*1.6*	*2.3*
**BASO (%)**	*0.00-2.25*	*0.7*	*0.0*	*0.8*	*0.0*	*0.2*	*0.3*

WBC: *White blood cell;* ***NEUT***: *Neutrophils;* ***LYMPH***: *Lymphocytes;* ***MONO***: *Mononucleosis;* ***EO***: *Eosinophil;* ***BASO***: *Basophils*

Disclaimer: *Reference ranges are based on published data sourced from Charles River and IDEXX laboratories, and are strain-specific for mice whenever possible. Published values do not necessarily correlate with in-house values, due to variability in testing equipment, background strain/substrain, sex, and age differences. Because of this variability, it is recommended that values be compared to control animals whenever possible.*

Supplementary Table 8. Complete manual differential blood counts of mice injected with saline vehicle (**Control group**, n=3) versus DFO-scFv-PEG-Cy5-C' dots (**Treatment group**, 2 nmols/mouse, n=3).

Test Name	Reference	*#1 Saline*	*#2 Saline*	*#3 Saline*	*#1 DFO-scFv-PEG-Cy5-C'dots*	*#2 DFO-scFv-PEG-Cy5-C'dots*	*#3 DFO-scFv-PEG-Cy5-C'dots*
**Neut# (K/uL)**	*0.43-3.58*	*0.58*	*0.00*	*0.00*	*0.47*	*1.62*	*1.07*
**Band# (K/uL)**	---	*0.00*	*0.00*	*0.00*	*0.00*	*0.00*	*0.00*
**LYMPH# (K/uL)**	*0.49-6.87*	*1.87*	*0.00*	*0.00*	*1.51*	*3.87*	*2.08*
**MONO# (K/uL)**	*0.03-0.65*	*0.00*	*0.00*	*0.00*	*0.00*	*0.00*	*0.00*
**EO# (K/uL)**	*0.01-0.53*	*0.30*	*0.00*	*0.00*	*0.00*	*0.06*	*0.18*
**NEUT (%)**	*0.43-3.58*	*21*	---	---	*23*	*28*	*31*
**LYMPH (%)**	*23.63-79.28*	*68*	---	---	*74*	*67*	*60*
**MONO (%)**	*0.86-12.15*	*6*	---	---	*3*	*3*	*6*
**EO (%)**	*0.29-7.18*	*5*	---	---	---	*2*	*3*
**Morphology**		WBC morphology is within normal limits.	A manual differential was not performed due to low WBC count (<2.00 K/uL).	A manual differential was not performed due to low WBC count (<2.00 K/uL).	WBC morphology is within normal limits.	WBC morphology is within normal limits.	WBC morphology is within normal limits.

NEUT*: Neutrophils;* ***LYMPH****: Lymphocytes;* ***MONO****: Mononucleosis;* ***EO****: Eosinophil*

Disclaimer*: Reference ranges are based on published data sourced from Charles River and IDEXX laboratories, and are strain-specific for mice whenever possible. Published values do not necessarily correlate with in-house values, due to variability in testing equipment, background strain/substrain, sex, and age differences. Because of this variability, it is recommended that values be compared to control animals whenever possible.*

Supplementary Table 9. Single-dose toxicity testing in non-tumor-bearing mice 72 h after systemic administration of saline vehicle (**Control group**, n=3) versus DFO-scFv-PEG-Cy5-C' dots (**Treatment group**, 2 nmols/mouse, n=3) indicates no tissue-specific pathological effects

Animal ID	***Anatomic Pathology***		
	Final Diagnosis(es)	***Gross Finding(s)***	***Microscopic Finding(s)***
#1 Saline	• *Liver: Minimal extramedullary hematopoiesis, multifocal.*<br>• *Mesenteric lymph node: Follicular lymphoid hyperplasia.*<br>• *Spleen: Follicular lymphoid hyperplasia.*<br>• *Skin (trunk, head): Mild acanthosis and hyperkeratosis, multifocal.*	• *Received a live adult female hairless mouse.*<br>• *Nutritional condition is normal (3/5 score)*<br>• *Body weight is 20.07 g.*<br>• *No gross lesions are observed.*	***Slides examined include the following tissues. All tissues are normal unless otherwise described.***<br>• *Heart, lungs.*<br>• *Kidneys, liver, gallbladder.*<br>• *Stomach, duodenum, jejunum, ileum, cecum, colon, salivary glands, mesenteric lymph nodes, submandibular lymph nodes.*<br>• *Uterus, cervix, vagina, urinary bladder.*<br>• *Adrenal, ovaries, oviducts, spleen, pancreas.*<br>• *Skin (trunk), thyroid, esophagus, trachea.*<br>• *Hind limb (femur, tibia, stifle joint, skeletal muscle, peripheral nerves), vertebral column with spinal cord, sternum, bone*<br>• *marrow in all bones listed.*<br>• *Head, coronal sections (including brain, eye, ears, pituitary, nasal and oral cavities, teeth, soft tissues, skin).*<br>• *Liver: Minimal extramedullary hematopoiesis, multifocal. Mesenteric lymph node: Follicular lymphoid hyperplasia.*<br>• *Spleen: Follicular lymphoid hyperplasia.*<br>• *Skin (trunk, head): Mild acanthosis and hyperkeratosis, multifocal.*
#2 Saline	• *Skin (trunk): Mild acanthosis and hyperkeratosis, multifocal.*<br>• *Mild dermatitis with furunculosis, pyogranulomatous, multifocal.*	• *Received a live adult female hairless mouse.*<br>• *Nutritional condition is normal (3/5 score)*<br>• *Body weight is 22.62 g.*<br>• *No gross lesions are observed.*	***Slides examined include the following tissues. All tissues are normal unless otherwise described.***<br>• *Heart, lungs, thymus, mediastinal lymph node.*<br>• *Kidneys, liver.*<br>• *Stomach, duodenum, jejunum, ileum, cecum, colon, salivary glands, mesenteric lymph nodes, submandibular lymph nodes.*<br>• *Uterus, cervix, vagina, urinary bladder.*<br>• *Adrenal, ovaries, oviducts, spleen, pancreas.*<br>• *Skin (trunk), mammary gland, thyroid, esophagus, trachea.*<br>• *Hind limb (femur, tibia, stifle joint, skeletal muscle, peripheral nerves), vertebral column with spinal cord, sternum, bone marrow in all bones listed.*<br>• *Head, coronal sections (including brain, eye, ears, pituitary, nasal and oral cavities, teeth, soft tissues, skin).*<br>• *Skin (trunk): Mild acanthosis and hyperkeratosis, multifocal. Mild dermatitis with furunculosis, pyogranulomatous, multifocal.*

#3 Saline	• *Mesenteric lymph node: Follicular lymphoid hyperplasia.*<br>• *Submandibular lymph node: Follicular lymphoid hyperplasia. - Spleen: Follicular lymphoid hyperplasia.*<br>• *Skin (trunk): Mild acanthosis and hyperkeratosis, multifocal.*	• *Received a live adult female hairless mouse.*<br>• *Nutritional condition is normal (3/5 score)*<br>• *bodB weight is 22.70 g.*<br>• *No gross lesions are observed.*	***Slides examined include the following tissues. All tissues are normal unless otherwise described.***<br>• *Heart, lungs, thymus, mediastinal lymph node.*<br>• *Kidneys, liver, gallbladder.*<br>• *Stomach, duodenum, jejunum, ileum, cecum, colon, salivary glands, mesenteric lymph nodes, submandibular lymph nodes.*<br>• *Uterus, cervix, vagina, urinary bladder.*<br>• *Adrenal, ovaries, oviducts, spleen, pancreas.*<br>• *Skin (trunk), mammary gland, thyroid, esophagus, trachea.*<br>• *Hind limb (femur, tibia, stifle joint, skeletal muscle, peripheral nerves), vertebral column with spinal cord, sternum, bone marrow in all bones listed.*<br>• *Head, coronal sections (including brain, eye, ears, pituitary, nasal and oral cavities, teeth, soft tissues, skin).*<br>• *Mesenteric lymph node: Follicular lymphoid hyperplasia. Submandibular lymph node: Follicular lymphoid hyperplasia. Spleen: Follicular lymphoid hyperplasia.*<br>• *Skin (trunk): Mild acanthosis and hyperkeratosis, multifocal.*
#1 DFO-scFv-PEG-Cy5-C'dots	• *Liver: Minimal extramedullary hematopoiesis, multifocal.*<br>• *Skin (trunk): Mild acanthosis and hyperkeratosis, multifocal.*<br>• *Mild dermatitis, pyogranulomatous, multifocal.*	• *Received a live adult female hairless mouse.*<br>• *Nutritional condition is normal (3/5 score)*<br>• *Body weight is 19.87 g.*<br>• *No gross lesions are observed.*	***Slides examined include the following tissues. All tissues are normal unless otherwise described.***<br>• *Heart, lungs, thymus.*<br>• *Kidneys, liver, gallbladder.*<br>• *Stomach, duodenum, jejunum, ileum, cecum, colon, salivary glands, submandibular lymph nodes.*<br>• *Uterus, cervix, vagina, urinary bladder.*<br>• *Adrenal, ovaries, oviducts, spleen, pancreas.*<br>• *Skin (trunk), mammary gland, thyroid, esophagus, trachea.*<br>• *Hind limb (femur, tibia, stifle joint, skeletal muscle, peripheral nerves), vertebral column with spinal cord, sternum, bone marrow in all bones listed.*<br>• *Head, coronal sections (including brain, eye, ears, pituitary, nasal and oral cavities, teeth, soft tissues, skin).*<br>• *Liver: Minimal extramedullary hematopoiesis, multifocal.*<br>• *Skin (trunk): Mild acanthosis and hyperkeratosis, multifocal. Mild dermatitis, pyogranulomatous, multifocal.*

#2 DFO-scFv-PEG-Cy5-C'dots	• *Skin (trunk, head): Mild acanthosis and hyperkeratosis, multifocal.*	• *Received a live adult female hairless mouse.*<br>• *Nutritional condition is normal (3/5 score)*<br>• *Body weight is 20.30 g.*<br>• *No gross lesions are observed.*	***Slides examined include the following tissues. All tissues are normal unless otherwise described.***<br>• *Heart, lungs.*<br>• *Kidneys, liver, gallbladder.*<br>• *Stomach, duodenum, jejunum, ileum, cecum, colon, salivary glands, mesenteric lymph nodes, submandibular lymph nodes.*<br>• *Uterus, cervix, vagina, urinary bladder.*<br>• *Adrenal, ovaries, oviducts, spleen, pancreas.*<br>• *Skin (trunk), mammary gland, thyroid, parathyroid, esophagus, trachea.*<br>• *Hind limb (femur, tibia, stifle joint, skeletal muscle, peripheral nerves), vertebral column with spinal cord, sternum, bone marrow in all bones listed.*<br>• *Head, coronal sections (including brain, eye, ears, pituitary, nasal and oral cavities, teeth, soft tissues, skin).*<br>• *Skin (trunk, head): Mild acanthosis and hyperkeratosis, multifocal.*
#3 DFO-scFv-PEG-Cy5-C'dots	• *Liver: Minimal extramedullary hematopoiesis, multifocal.*<br>• *Spleen: Follicular lymphoid hyperplasia.*<br>• *Skin (trunk, head): Mild acanthosis and hyperkeratosis, multifocal.*	• *Received a live adult female hairless mouse.*<br>• *Nutritional condition is normal (3/5 score)*<br>• *Body weight is 23.35 g.*<br>• *No gross lesions are observed.*	***Slides examined include the following tissues. All tissues are normal unless otherwise described.***<br>• *Heart, lungs, thymus.*<br>• *Kidneys, liver, gallbladder.*<br>• *Stomach, duodenum, jejunum, ileum, cecum, colon, salivary glands, mesenteric lymph nodes, submandibular lymph nodes.*<br>• *Uterus, cervix, vagina, urinary bladder.*<br>• *Adrenal, ovaries, oviducts, spleen, pancreas.*<br>• *Skin (trunk), mammary gland, esophagus, trachea.*<br>• *Hind limb (femur, tibia, stifle joint, skeletal muscle, peripheral nerves), vertebral column with spinal cord, sternum, bone marrow in all bones listed.*<br>• *Head, coronal sections (including brain, eye, ears, pituitary, nasal and oral cavities, teeth, soft tissues, skin).*<br>• *Liver: Minimal extramedullary hematopoiesis, multifocal.*<br>• *Spleen: Follicular lymphoid hyperplasia.*<br>• *Skin (trunk, head): Mild acanthosis and hyperkeratosis, multifocal.*

Supplementary Table 10. Clinical trial summary indicating the status of anti-HER2 PET imaging probes for breast cancer management in human subjects (recruiting / completed) [a,b]

	ClinicalTrials.gov Identifier	Official Title	Status	Probe name	Purpose	Location
1	NCT00605397	Pilot Trial of PET Imaging With Cu-64 Labeled Trastuzumab in HER2+ Metastatic Breast Cancer	Phase I, Completed	64Cu-Labeled Trastuzumab	Evaluation of Cu-64 Labeled Trastuzumab as a PET agent for detecting metastatic breast cancer	Memorial Sloan Kettering Cancer Center, New York, United States
2	NCT02827877	Use of 64Cu-DOTA-Trastuzumab PET Imaging and Molecular Markers for Prediction of Response to Trastuzumab and Pertuzumab-Based Neoadjuvant Therapy	Phase I, Recruiting	64Cu-DOTA-Trastuzumab	Evaluate ability of Cu64-DOTA-trastuzumab PET to predict treatment response following administration of trastuzumab and pertuzumab prior to surgery in HER2+ breast cancer patients	City of Hope Medical Center, Duarte, California, United States
3	NCT01858116	An Exploratory Study to Evaluate [68Ga]ABY-025 for PET Imaging of HER2 Expression in Subjects With Metastatic Breast Cancer	Phase I&II, Complated	[68Ga]ABY-025	Assess [68Ga]ABY-025 as a PET imaging agent for detecting HER2-positive versus HER2-negative breast cancer	Uppsala University Hospital, Uppsala, Sweden
4	NCT02065609	Assessment of HER2 Receptors in Breast Carcinoma by Positron Emission Tomography (PET) Using 89Zr-Trastuzumab	Early Phase I, Recruiting	89Zr-Trastuzumab	Evaluation of 89Zr labeled trastuzumab as a PET agent for detection of HER2+ breast cancer.	Washington University School of Medicine @ Barnes-Jewish Hospital, Saint Louis, Missouri, United States
5	NCT01081600	Imaging the Effect of HSP90 Inhibitor AUY922 on HER2 Expression by Means of 89Zr-Trastuzumab PET	Phase I, Completed	89Zr-Trastuzumab	Visualize HER2 expression in breast cancer patients before and after HSP90 target inhibition using 89Zr-trastuzumab PET	University Medical Center Groningen, Groningen, Netherlands
6	NCT01957332	Towards Patient Tailored Cancer Treatment Supported by Molecular Imaging IMPACT: IMaging PAtients for Cancer Drug selecTion - Metastatic Breast Cancer	Phase I&II, Recruiting	89Zr-Trastuzumab	Assessment of estrogen receptor and/or HER2 receptor status via PET imaging	VU University Medical Center, Amsterdam, Netherlands
7	NCT01832051	HER2-PET as a Diagnostic Tool in Breast Cancer Patients With a Clinical Dilemma	Phase II, Completed	89Zr-Trastuzumab	Whole body visualization and quantification of HER2 expression using 89Zr-Trastuzumab PET imaging	University Medical Center Groningen, Groningen, Netherlands
8	NCT01420146	Pilot Imaging Study With 89Zr-Trastuzumab in HER2-positive Metastatic Breast Cancer Patients : Correlation With FDG-PET/CT and Anatomopathological Results	Phase I, Completed	89Zr-Trastuzumab	Evaluation of the diagnostic potential of HER2 imaging using 89Zr-Trastuzumab imaging	Jules Bordet Institut, Brussels, Belgium
9	NCT01565200	Phase II Prospective Imaging Study Evaluating the Utility of Pre-treatment Zr89-Trastuzumab PET/CT and Early FDG-PET/CT Responses to Identify Patients With Advanced HER2+ BC Unlikely to Benefit From a Novel antiHER2 Therapy: TDM1	Phase II, Recruiting	89Zr-Trastuzumab	Identify patients unlikely to benefit fromTDM-1 therapy via 89Zr-Trastuzumab PET imaging	Universitair Ziekenhuis Antwerpen, Antwerpen, Edegem, Belgium

a: Trials with a status of "*Active, not recruiting*". ^{64}Cu-labeled Trastuzumab (NCT01093612, NCT02226276). ^{89}Zr-Trastuzumab (NCT02286843). ^{18}F-FDG (NCT01937117).

b: Trials with a status of "*Unknown*". [^{68}GA]ABY-025 (NCT02095210).

Supplementary References

1. Chen, F. et al. "Target-or-Clear" Zirconium-89 Labeled Silica Nanoparticles for Enhanced Cancer-Directed Uptake in Melanoma: A Comparison of Radiolabeling Strategies. *Chem. Mater.*, DOI: 10.1021/acs.chemmater.1027b02567 (2017).
2. Ma, K. & Wiesner, U. Modular and Orthogonal Post-PEGylation Surface Modifications by Insertion Enabling Penta-Functional Ultrasmall Organic-Silica Hybrid Nanoparticles. *Chem Mater* **29**, 6840-6855 (2017).
3. Larson, D.R. et al. Silica Nanoparticle Architecture Determines Radiative Properties of Encapsulated Fluorophores. *Chemistry of Materials* **20**, 2677-2684 (2008).
4. Holland, J.P., Sheh, Y. & Lewis, J.S. Standardized methods for the production of high specific-activity zirconium-89. *Nucl Med Biol* **36**, 729-739 (2009).
5. Stabin, M.G., Sparks, R.B. & Crowe, E. OLINDA/EXM: the second-generation personal computer software for internal dose assessment in nuclear medicine. *J Nucl Med* **46**, 1023-1027 (2005).
6. Zhang, H. et al. Dual-Modality Imaging of Prostate Cancer with a Fluorescent and Radiogallium-Labeled Gastrin-Releasing Peptide Receptor Antagonist. *J Nucl Med* **58**, 29-35 (2017).
7. Blanco, E., Shen, H. & Ferrari, M. Principles of nanoparticle design for overcoming biological barriers to drug delivery. *Nat Biotech* **33**, 941-951 (2015).
8. Ma, K., Zhang, D., Cong, Y. & Wiesner, U. Elucidating the Mechanism of Silica Nanoparticle PEGylation Processes Using Fluorescence Correlation Spectroscopies. *Chem Mater* **28**, 1537-1545 (2016).
9. Phillips, E. et al. Clinical translation of an ultrasmall inorganic optical-PET imaging nanoparticle probe. *Sci Transl Med* **6**, 260ra149 (2014).
10. Burns, A.A. et al. Fluorescent Silica Nanoparticles with Efficient Urinary Excretion for Nanomedicine. *Nano Lett* **9**, 442-448 (2009).
11. Ma, K. et al. Control of Ultrasmall Sub-10 nm Ligand-Functionalized Fluorescent Core-Shell Silica Nanoparticle Growth in Water. *Chem Mater* **27**, 4119-4133 (2015).
12. Chen, F. et al. Cancer-Targeting Ultrasmall Silica Nanoparticles for Clinical Translation: Physicochemical Structure and Biological Property Correlations. *Chem Mater* **29**, 8766-8779 (2017).
13. Abou, D.S., Ku, T. & Smith-Jones, P.M. In vivo biodistribution and accumulation of 89Zr in mice. *Nucl Med Biol* **38**, 675-681 (2011).
14. Chen, F. et al. Cancer-Targeting Ultrasmall Silica Nanoparticles for Clinical Translation: Physicochemical Structure and Biological Property Correlations. *Chem. Mater.* (2017).
15. Benezra, M. et al. Multimodal silica nanoparticles are effective cancer-targeted probes in a model of human melanoma. *J Clin Invest* **121**, 2768-2780 (2011).
16. Zhang, Y.N., Poon, W., Tavares, A.J., McGilvray, I.D. & Chan, W.C. Nanoparticle-liver interactions: Cellular uptake and hepatobiliary elimination. *J Control Release* **240**, 332-348 (2016).
17. Choi, H.S. et al. Design considerations for tumour-targeted nanoparticles. *Nat Nanotechnol* **5**, 42-47 (2010).
18. Zhou, C. et al. Near-infrared emitting radioactive gold nanoparticles with molecular pharmacokinetics. *Angew Chem Int Ed Engl* **51**, 10118-10122 (2012).
19. Laforest, R. et al. [89Zr]Trastuzumab: Evaluation of Radiation Dosimetry, Safety, and Optimal Imaging Parameters in Women with HER2-Positive Breast Cancer. *Mol Imaging Biol* **18**, 952-959 (2016).
20. Choi, H.S. et al. Renal Clearance of Nanoparticles. *Nat Biotechnol* **25**, 1165-1170 (2007).
21. Chen, F. et al. Dynamic Positron Emission Tomography Imaging of Renal Clearable Gold Nanoparticles. *Small* **12**, 2775-2782 (2016).
22. Zhou, M. et al. CuS Nanodots with Ultrahigh Efficient Renal Clearance for Positron Emission Tomography Imaging and Image-Guided Photothermal Therapy. *ACS Nano* **9**, 7085-7096 (2015).

23. Chen, F. et al. Target-or-Clear Zirconium-89 Labeled Silica Nanoparticles for Enhanced Cancer-Directed Uptake in Melanoma: A Comparison of Radiolabeling Strategies. *Chem Mater* **29**, 8269-8281 (2017).
24. Chen, F. et al. Melanocortin-1 Receptor-Targeting Ultrasmall Silica Nanoparticles for Dual-Modality Human Melanoma Imaging. *ACS Appl Mater Interfaces* **10**, 4379-4393 (2018).
25. Zolata, H., Abbasi Davani, F. & Afarideh, H. Synthesis, characterization and theranostic evaluation of Indium-111 labeled multifunctional superparamagnetic iron oxide nanoparticles. *Nucl Med Biol* **42**, 164-170 (2015).
26. Chen, K., Li, Z.B., Wang, H., Cai, W. & Chen, X. Dual-modality optical and positron emission tomography imaging of vascular endothelial growth factor receptor on tumor vasculature using quantum dots. *Eur J Nucl Med Mol Imaging* **35**, 2235-2244 (2008).
27. Yang, M. et al. Affibody modified and radiolabeled gold-iron oxide hetero-nanostructures for tumor PET, optical and MR imaging. *Biomaterials* **34**, 2796-2806 (2013).
28. Karmani, L. et al. Antibody-functionalized nanoparticles for imaging cancer: influence of conjugation to gold nanoparticles on the biodistribution of 89Zr-labeled cetuximab in mice. *Contrast Media Mol Imaging* **8**, 402-408 (2013).
29. Lee, H.Y. et al. PET/MRI dual-modality tumor imaging using arginine-glycine-aspartic (RGD)-conjugated radiolabeled iron oxide nanoparticles. *J Nucl Med* **49**, 1371-1379 (2008).
30. Black, K.C. et al. Radioactive 198Au-doped nanostructures with different shapes for in vivo analyses of their biodistribution, tumor uptake, and intratumoral distribution. *ACS Nano* **8**, 4385-4394 (2014).
31. Lin, Y.Y. et al. Pharmacokinetics and dosimetry of (111)In/(188)Re-labeled PEGylated liposomal drugs in two colon carcinoma-bearing mouse models. *Cancer Biother Radiopharm* **26**, 373-380 (2011).
32. Goel, S. et al. VEGF121-Conjugated Mesoporous Silica Nanoparticle: A Tumor Targeted Drug Delivery System. *ACS Applied Materials & Interfaces* **6**, 21677-21685 (2014).
33. Chen, F. et al. In Vivo Tumor Vasculature Targeting of CuS@MSN Based Theranostic Nanomedicine. *ACS Nano* **9**, 3926-3934 (2015).
34. Chen, F. et al. In vivo tumor vasculature targeted PET/NIRF imaging with TRC105(Fab)-conjugated, dual-labeled mesoporous silica nanoparticles. *Mol Pharm* **11**, 4007-4014 (2014).
35. Chen, F. et al. Engineering of hollow mesoporous silica nanoparticles for remarkably enhanced tumor active targeting efficacy. *Sci Rep* **4**, 5080 (2014).
36. Kommareddy, S. & Amiji, M. Biodistribution and pharmacokinetic analysis of long-circulating thiolated gelatin nanoparticles following systemic administration in breast cancer-bearing mice. *J Pharm Sci* **96**, 397-407 (2007).
37. Liu, Z. et al. In vivo biodistribution and highly efficient tumour targeting of carbon nanotubes in mice. *Nat Nanotechnol* **2**, 47-52 (2007).
