# Supplementary material for: Ultrasmall targeted nanoparticles with engineered antibody fragments for imaging detection of HER2-overexpressing breast cancer
Source: Nat Commun. 2018 Oct 8;9:4141. doi: 10.1038/s41467-018-06271-5 (PMC6175906; doi:10.1038/s41467-018-06271-5)
Supplement: Supplementary file 4 — Description of Additional Supplementary Files [file 41467_2018_6271_MOESM4_ESM.docx]

**Description of Additional Supplementary Files**

File Name: Supplementary Movie 1

Description: Representative PET/CT images of BT-474 tumor-bearing mice injected with 89Zr-DFO-scFv-PEG-Cy5-C’ dots at 48 hours p.i.
