# Supplementary material for: Ultrasmall targeted nanoparticles with engineered antibody fragments for imaging detection of HER2-overexpressing breast cancer
Source: Nat Commun. 2018 Oct 8;9:4141. doi: 10.1038/s41467-018-06271-5 (PMC6175906; doi:10.1038/s41467-018-06271-5)
Supplement: Supplementary file 2 — Peer Review File [file 41467_2018_6271_MOESM2_ESM.pdf]

Editorial Note: this manuscript has been previously reviewed at another journal that is not operating a transparent peer review scheme.
